# Mid‐life leukocyte telomere length and dementia risk: An observational and mendelian randomization study of 435,046 UK Biobank participants

**DOI:** 10.1111/acel.13808

**Published:** 2023-05-30

**Authors:** Rui Liu, Meiruo Xiang, Luke C. Pilling, David Melzer, Lihong Wang, Kevin J. Manning, David C. Steffens, Jack Bowden, Richard H. Fortinsky, George A. Kuchel, Taeho G. Rhee, Breno S. Diniz, Chia‐Ling Kuo

**Affiliations:** ^1^ Department of Health Sciences Sacred Heart University Fairfield Connecticut USA; ^2^ Connecticut Convergence Institute for Translation in Regenerative Engineering University of Connecticut Health Farmington Connecticut USA; ^3^ Epidemiology and Public Health Group, College of Medicine and Health University of Exeter Exeter UK; ^4^ Department of Psychiatry University of Connecticut Health Farmington Connecticut USA; ^5^ Exeter Diabetes Group (ExCEED), College of Medicine and Health University of Exeter Exeter UK; ^6^ UConn Center on Aging University of Connecticut Health Farmington Connecticut USA; ^7^ Department of Public Health Sciences University of Connecticut Health Farmington Connecticut USA

**Keywords:** Alzheimer's disease, brain magnetic resonance imaging, cognition, hallmarks of biological aging, vascular dementia

## Abstract

Telomere attrition is one of biological aging hallmarks and may be intervened to target multiple aging‐related diseases, including Alzheimer's disease and Alzheimer's disease related dementias (AD/ADRD). The objective of this study was to assess associations of leukocyte telomere length (TL) with AD/ADRD and early markers of AD/ADRD, including cognitive performance and brain magnetic resonance imaging (MRI) phenotypes. Data from European‐ancestry participants in the UK Biobank (*n* = 435,046) were used to evaluate whether mid‐life leukocyte TL is associated with incident AD/ADRD over a mean follow‐up of 12.2 years. In a subsample without AD/ADRD and with brain imaging data (*n* = 43,390), we associated TL with brain MRI phenotypes related to AD or vascular dementia pathology. Longer TL was associated with a lower risk of incident AD/ADRD (adjusted Hazard Ratio [aHR] per SD = 0.93, 95% CI 0.90–0.96, *p* = 3.37 × 10^−7^). Longer TL also was associated with better cognitive performance in specific cognitive domains, larger hippocampus volume, lower total volume of white matter hyperintensities, and higher fractional anisotropy and lower mean diffusivity in the fornix. In conclusion, longer TL is inversely associated with AD/ADRD, cognitive impairment, and brain structural lesions toward the development of AD/ADRD. However, the relationships between genetically determined TL and the outcomes above were not statistically significant based on the results from Mendelian randomization analysis results. Our findings add to the literature of prioritizing risk for AD/ADRD. The causality needs to be ascertained in mechanistic studies.

## Graphical abstract

Longer leukocyte telomere length was associated with reduced risks of AD/ADRD, cognitive impairment, and brain structural lesions.

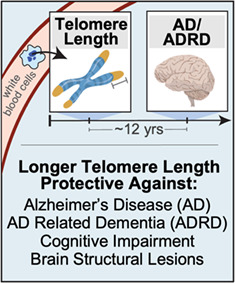

**Abbreviations**
AD/ADRD: Alzheimer's disease (AD) and Alzheimer's disease related dementias
aHR: adjusted hazard ratio
APOE: ApolIpoprotein E
BMI: body mass index
FDR: false discovery rate
GWAS: genome‐wide association study
ICD: International Classification of Diseases
IDP: imaging derived phenotype
IVW: inverse‐variance weighting
MR: Mendelian randomization
MR‐RAPS: Mendelian randomization ‐ robust adjusted profile score
NOME: no measurement error
OR: odds ratio
PC: principal component
SD: standard deviation
SNP: single nucleotide polymorphism
*T*/*S* ratio: ratio comparing the amount of the telomere amplification product (*T*) to that of a single‐copy gene (*S*)
TL: telomere length
UKB: UK Biobank
WMH: white matter hyperintensities

## 1 INTRODUCTION

Telomeres are repetitive sequences at the end of chromosomes, where they protect DNA from damage and preserving genome stability (Blackburn et al., 2015). In somatic cells, telomeres shorten with each cell division and are replenished by the telomerase enzyme activity (Collins & Mitchell, 2002). Critically short telomere length (TL) signals cells to stop replicating and can trigger cellular senescence changes (Bernardes de Jesus & Blasco, 2013). A significant consequence of cellular senescence is the change in the cellular secretome and a shift towards a pro‐inflammatory state (i.e., the Senescence‐Associated Secretory Phenotype, SASP) that can exert deleterious effects in different tissues and organs, including the brain (Ovadya & Krizhanovsky, 2014). Shorter leukocyte TL is associated with increased mortality risks (Wang et al., 2018) and cardiovascular disease (Haycock et al. 2014). In contrast, longer leukocyte TL may increase risks of certain cancers, including glioma, ovarian, and lung cancer (Zhang et al., 2017).

Previous studies suggest that TL may also play an important role in the development of neurodegeneration and neurodegenerative disorders (Levstek et al., 2020). Alzheimer's disease (AD) is the most prevalent neurodegenerative disease associated with aging. Previous conflicting associations between TL and AD (Forero et al., 2016; Scarabino et al., 2017) may be explained by small samples, pathology of other neurodegenerative disorders in AD patients, and controls including possible preclinical AD cases. Due to shared pathological features, AD, frontotemporal, Lewy body, vascular, and mixed dementia are classified as AD and AD‐related dementias (AD/ADRD) for research purposes (Corriveau et al., 2017). Cognitive decline and brain changes occur many years before AD diagnosis (Jack & Holtzman, 2013). Recent meta‐analyses reported that longer TL was associated with better general cognition (Zhan et al., 2018) and performance in several cognitive domains (Gampawar et al., 2020; Hägg et al., 2017). Few population‐based studies have explored TL in relation to brain MRI imaging features (Gampawar et al., 2020; King et al., 2014; Suchy‐Dicey et al., 2018). A recent meta‐analysis showed that longer leukocyte TL is associated with whole brain and hippocampus volumes but not with white matter hyperintensities (WMH) (Gampawar et al., 2022). In contrast, critically short TL has been associated with a more rapid cognitive decline and conversion from mild cognitive impairment to AD in a two‐year follow‐up study of older adults (Koh et al., 2020).

We leveraged data from a large prospective cohort in the UK Biobank (UKB) to not only estimate associations between TL and risk of incident AD/ADRD, but to also assess associations with early markers of AD/ADRD, including cognitive performance and brain imaging derived phenotypes (IDPs) (Bordes et al., 2022; Wu et al., 2019) in participants who were dementia‐free at the time of multi‐modality imaging assessment. Our primary analysis was the analysis using the observational data. While we included a number of covariates in the models for adjustment, there is a possibility that the results may be biased by unmeasured confounding. As a means of avoiding confounding, we conducted the genetics‐based Mendelian randomization (MR) analysis (secondary analysis) to estimate the associations of genetically determined TL with the outcomes. Although the effect sizes are not comparable, evidence for the observational associations is strengthened with the genetic evidence support.

## 2 MATERIALS AND METHODS

### 2.1 UK biobank

UKB is a volunteer community cohort recruiting over 500,000 volunteers aged 40–69 years between 2006 and 2010 (Bycroft et al., 2018; Sudlow et al., 2015). At recruitment, participants completed an extensive questionnaire and provided biological samples for genetic and other future assays. Since 2014, UKB re‐invited participants to undergo a multimodal imaging assessment of the brain, heart, and body. During the visit, baseline and additional cognitive tests were administered online.

### 2.2 Inclusion and exclusion criteria

**FIGURE 1:**
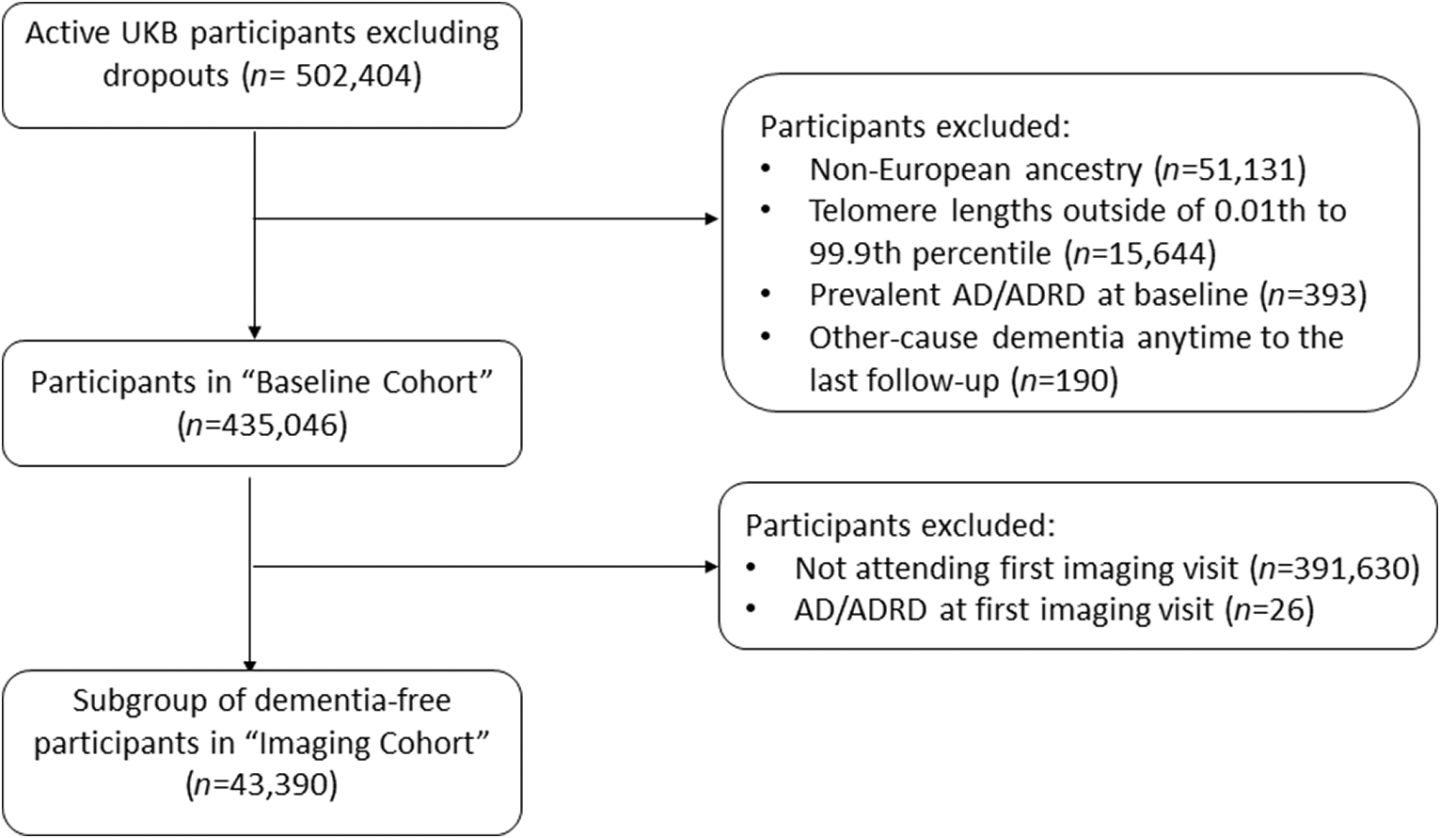
Flowchart of study participant selection.

Data were from active UKB participants. We excluded from our analysis participants with (1) non‐European ancestry (*n* = 51,131) to avoid confounding from ancestry differences; (2) extreme TL <0.01st or > 99.9th percentile (*n* = 15,644); (3) diagnosed with AD/ADRD prior to or at baseline (*n* = 393); and (4) diagnosed with other cause dementia at any time before the last follow‐up (*n* = 190) (Figure 1). The baseline cohort (*N* = 435,046) was used to assess associations of TL with incident AD/ADRD and baseline cognitive performance. A subsample who attended the first imaging visit between 2014 and 2019 and were free of AD/ADRD, termed “imaging cohort,” was used to test for associations of TL with IDPs and performance of cognitive tests first implemented at the first imaging visit. For specific analyses, participants with any missing outcome or covariates were further excluded.

### 2.3 Data

#### 2.3.1 Telomere length measurement

Relative mean TL was measured from peripheral blood leukocytes in T/S ratio using a multiplex quantitative polymerase chain reaction technique by comparing the amount of the telomere amplification product (T) to that of a single‐copy gene (S) (Codd et al., 2022). TL was adjusted for the influence of technical parameters and was log‐ and z‐transformed among the included samples before analysis.

#### 2.3.2 Genotype data

UKB participants were genotyped using DNA extracted from the baseline blood samples (Bycroft et al., 2018). Genetic principal components (PCs) to account for ancestry differences were derived within participants of European descent using the genome‐wide genotype data. *APOE* genotypes were determined based on the genotypes of the two single nucleotide polymorphisms (SNPs), rs429358 and rs7412, on chromosome 19. Genetic variants strongly associated with leukocyte TL at the genome‐wide significance level (*p* < 8.31 × 10^−9^) in the UKB GWAS (Codd et al., 2021) were selected as genetic instruments (*n* = 130) (Table S1) to estimate TL‐outcome associations using MR methods. These genetic variants were uncorrelated and robust to pleiotropy, and they were enriched with functional variants near genes underlying telomere and telomerase biology (Codd et al., 2021).

#### 2.3.3 Assessment of outcomes

AD/ADRD was confirmed using the first occurrence data of UKB by mapping multi‐source data to 3‐character International Classification Diseases (ICD) codes (Table S2). A dementia case was first diagnosed with AD/ADRD or other‐cause dementia (ICD‐10: F02) depending on which occurred first. Other‐cause dementias as described previously were excluded from our analyses. AD only and vascular dementia (VD) only were analyzed as subtypes of AD/ADRD.

We selected UKB cognitive tests a priori, previously shown to have moderate to high concurrent validity with well‐validated reference tests and test–retest reliability (specifically, reaction time, numeric memory, symbol digit substitution, trail making part B, and matrix pattern completion) (Fawns‐Ritchie & Deary, 2020). We also included tests mostly correlated with general cognitive ability, that is, fluid intelligence (Fawns‐Ritchie & Deary, 2020), and derived general cognitive ability scores combining five baseline cognitive test scores via the first principal component from Fawns‐Ritchie et al. (Fawns‐Ritchie & Deary, 2020). Data were from baseline or the first imaging visit depending on when the cognitive test was first implemented. Cognitive assessments are detailed in Text S1. Their associated cognitive domains, data field IDs, and weblinks are provided in Table S3.

We utilized the IDPs generated by an image‐processing pipeline developed and run on behalf of UKB (Alfaro‐Almagro et al., 2018). Specifically, we selected IDPs associated with dementia (Bordes et al., 2022; Wu et al., 2019): (1) AD‐signature region volumes from T1‐weighted structural imaging: hippocampus, parahippocampal cortex, entorhinal cortex, inferior parietal lobule, precuneus, and cuneus; (2) total volume of WMH derived from combined T1 and T2‐weighted fluid‐attenuated inversion recovery (FLAIR) structural imaging; and (3) weighted mean fractional anisotropy (FA) and mean diffusivity (MD) of white matter tracts from diffusion‐weighted imaging. Left and right hemisphere measurements for the same tract were averaged before analysis. A description of the selected IDPs is provided in Table S4.

#### 2.3.4 Covariates

Baseline covariates were selected a priori as potential confounders for associations with TL. A previous UKB study reported that older age, male gender, White ethnicity, lower socioeconomic status, and unhealthy lifestyle are associated with shorter TL (Bountziouka et al., 2022). Another study showed that the mean TL was longer in *APOE* e4 carriers than in *APOE* e3e3 carriers (Wikgren et al., 2012), which was confirmed in our current study (results not shown). Lastly, head size was selected for examining the associations between TL and IDPs due to its associations with regional brain volumes.

Baseline covariates included demographics (age, sex, assessment center near residence), socioeconomic status (education, Townsend deprivation index), lifestyle factors (body mass index [BMI], smoking status, alcohol intake frequency, physical activity), *APOE* genotype (e3e4, e4e4, e2e4, e3e4, e4e4, or e1e2, versus e3e3), and top 10 genetic PCs (PC1‐PC10). We included PC1‐PC10 to adjust for ancestry differences in European‐ancestry participants, and assessment centers near residence which could be proxies for unmeasured confounders associated with spatial differences. Townsend deprivation index score was a measure of material deprivation at the postcode level based on the preceding national census data. Higher scores represent greater levels of deprivation. Smoking status and alcohol intake frequency were accessed through a touchscreen questionnaire. Physical activity (low, moderate, or high) was self‐reported and measured following the short International Physical Activity Questionnaire guidelines (Cassidy et al., 2016). Many of these covariates were associated with AD/ADRD and its subtypes (Tables S6–S8), even after adjusting for other covariates (Tables S9–S14).

### 2.4 Statistical methods

The association between TL and time from baseline to first incident AD/ADRD diagnosis was estimated using a Cox proportional hazards model. Previous studies reported a U‐shaped association between TL and risk of AD (Fani et al., 2020) or amnestic mild cognitive impairment (Roberts et al., 2014). To assess this possibility, the non‐linearity of TL association with incident AD/ADRD was examined using a penalized cubic spline function (R “pspline” function with default parameters). Participants who did not develop AD/ADRD but died during follow‐up were censored at date of death, otherwise, at the last follow‐up date (March 31, 2021). The Gönen & Heller's K‐index, a measure of overall predictive accuracy, was calculated for the Cox proportional hazards regression model to represent the probability that the failure time order is consistent with the risk score order for a random pair of subjects. The higher the K index, the greater the discriminative power the model has for the time‐to‐event outcome.

Sensitivity analyses were performed by *APOE* genotype (e3e3, e4 [e3e4 or e4e4], or e2 [e2e3 or e2e2]), for AD and VD, separately. Linear regression models were used to examine the associations of TL with cognitive measures and IDPs. Before modeling, each continuous outcome was transformed by the rank‐based inverse normal transformation to correct for the distributional skewness and to unify the scales. Each of the above models adjusting for all covariates as described in “Covariates” (full model [primary]). Unadjusted results (unadjusted model) and results adjusted for age and sex only (base model) are presented for comparison. IDPs were adjusted for head size additionally regardless of the models.

To estimate the causal effects of TL on AD/ADRD and related outcomes, we applied several MR methods to ensure our results are robust to MR assumptions: (1) inverse‐variance weighting (IVW) method (Burgess et al., 2013) (primary) that meta‐analyzes causal estimates from individual genetic instruments; (2) a weighted median‐based method (Bowden, Fabiola Del Greco, et al., 2016) that assumes that the majority of genetic variants are valid instrumental variables; (3) MR‐Egger (Bowden et al., 2015) that allows us to assess horizontal pleiotropy additionally; (4) robust adjusted profile score (MR‐RAPS) method (Zhao et al., 2020), accounting for any residual weak instrument bias, pleiotropy, and extreme outliers. Both instrument‐TL associations and instrument‐outcome associations from the present study were adjusted for age, sex, genotyping array, and PC1‐PC10, plus head size additionally for IDPs.

Prior to the MR analysis, the instrument‐TL associations (regression coefficients and standard errors) were adjusted for winner's curse using (see the method in Text S2 and R code in Text S3). Before and after winner's curse adjustment, we calculated the mean F‐statistic (Burgess & Thompson, 2011) and percent of variance in TL attributed to the genetic instruments to evaluate the weak instrument bias, and the I^2^‐statistic (Bowden, Davey Smith et al., 2016) to evaluate the no measurement error (NOME) assumption of MR‐Egger.

Observational *p*‐values or MR *p*‐values from the primary IVW method were evaluated at the level of false discovery rate (FDR) less than 5%. All the statistical analyses were performed in R version 3.4.1.

## 3 RESULTS

Participant characteristics at recruitment of the baseline cohort (*n* = 435,046) are presented in Table S5. The mean follow‐up time from baseline to first imaging visit for participants in the imaging cohort (*n* = 43,390) was 8.97 years (SD = 1.75). Participants who subsequently underwent the first imaging visit were healthier than the baseline sample (Table S5), which had also been shown by a previous study (Lyall et al., n.d.).

### 3.1 Observational association analysis

During a mean follow‐up of 12.2 years, we identified 6424 incident AD/ADRD cases (mean age at diagnosis 72.8 years, SD = 6.1): 1225 AD cases (mean age at diagnosis 74.2 years, SD = 5.1) and 602 VD cases (mean age at diagnosis 73.8 years, SD = 5.6). We categorized TL into six groups using the mean and standard deviation of TL: (1) short: (‐Inf, mean‐2SD); (2) moderately short: (mean‐2SD, mean‐SD); (3) slightly short: (mean‐SD, mean); (4) slightly long: (mean, mean + SD); (5) moderately long: (mean + SD, mean + 2SD); and (6) long: (mean + 2SD, Inf). The cumulative incidence of AD/ADRD increased over time and was consistently lower in participants with longer TL compared to those with shorter TL (Figure S1). Additionally, the hazard ratios (HR) comparing each longer TL group to the short TL group showed a decreasing trend: HR = 0.78 (95% CI 0.68–0.90, *p* = 7.2 × 10^−4^) for moderately short versus short, HR = 0.61 (95% CI 0.53–0.70, *p* < 2.2 × 10^−16^) for slightly short versus short, HR = 0.46 (95% CI 0.40–0.52, *p* < 2.2 × 10^−16^) for slightly long versus short, HR = 0.36 (95% CI 0.31–0.42, *p* < 2.2 × 10^−16^) for moderately long versus short, and HR = 0.26 (95% CI 0.20–0.35, *p* < 2.2 × 10^−16^) for long versus short. Compared with normal controls (0.83 ± 0.13), AD/ADRD cases (0.80 ± 0.12, *p* < 2.2 × 10^−16^) had a shorter mean TL and similarly, AD (0.80 ± 0.11, *p* < 2.2 × 10^−16^) and VD (0.79 ± 0.12, *p* < 2.2 × 10^−16^) cases. Regarding other participant characteristics, men were more prevalent in AD/ADRD cases than in normal controls. AD/ADRD cases were older, less educated, more subject to material deprivation, physically less active and had a slightly higher mean BMI. They also were more like to be a smoker, non‐drinker, or carrier of *APOE* e4 alleles (Table S6). Similar associations were found comparing AD or VD to normal controls. However, no statistically significant differences were found between AD cases and normal controls in sex, Townsend deprivation index, BMI, and IPAQ activity group (Tables S7 and S8).

We tested the non‐linearity of TL association with incident AD/ADRD comparing the full model including a penalized cubic spline function and that with a linear term of TL. The result was not statistically significant (*p* = 0.404), and was similar for that with AD only (*p* = 0.056) or VD only (*p* = 0.211), indicating that TL associations with AD/ADRD or the subtypes AD and VD were approximately linear. Next, we present the full model results adjusted for a full set of covariates including a linear term of TL. The full model results were similar to the base model results, suggesting that age and sex are major confounders among the covariates. In contrast, the unadjusted models were more liable to confounding bias and tended to show larger effect sizes, which may be of the same or opposite direction. The effect size of TL is presented as a hazard ratio per SD longer in TL for AD/ADRD and its subtypes, or a mean SD change per SD longer in TL for cognitive and brain MRI outcomes, which is more interpretable than a hazard ratio or man SD change per unit increase in T/S ratio. One SD of TL regardless of the unit corresponds to the absolute length of approximately 650 base pairs in a European adult population (see Supplemental Online Content of (Collaboration TMR et al., 2017) and Table 1 in (Mangino et al., 2012)), which is approximately 26 years of telomere attrition given the telomere shortening rate in the population is about 25 base pairs per year (Aviv & Shay, 2018). To facilitate the effect size comparison, we converted TL associations to the mean/risk changes associated with an *x*‐year increase/decrease of age using the effect size estimates of TL and age (in years) from the full models. The equivalent age effect was calculated by *x* = abs (β_TL_)/abs (β_age_), where abs was the absolute function and β_TL_ and β_age_ were the estimates of regression coefficients associated with TL and age in a fully adjusted Cox or linear regression model. For a time‐to‐event outcome, β_TL_ = ln (HR_TL_) and β_age_ = ln (HR_age_). Similarly, we converted associations with covariates to the equivalent age effects (results not shown in the text but can be found in Tables S9–S82).

**FIGURE 2:**
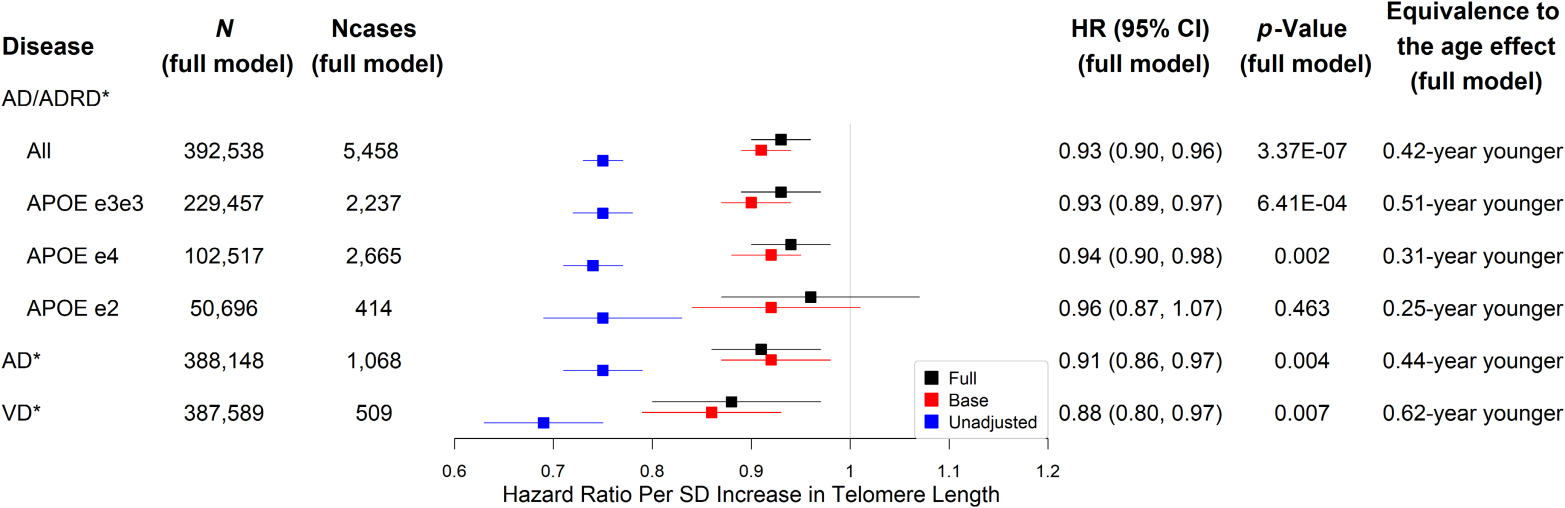
Associations between telomere length and AD/ADRD or related phenotypes. *Significant at the false discovery rate <0.05 level; AD: Alzheimer's disease (AD) or dementia in AD only; VD: vascular dementia; N (full model): sample size; NCases (full model): number of cases; Full: Cox proportional hazards models adjusting for age, sex, education, Townsend deprivation index, BMI, smoking status, alcohol intake frequency, IPAQ physical activity group, *APOE* genotype, PC1‐PC10, and baseline assessment center; Base: Cox proportional hazards models adjusting for age and sex only; Unadjusted: Cox regression models with no adjustment for covariates; *p*‐Value: unadjusted *p*‐value for multiple testing.

Longer leukocyte TL at baseline was associated with a lower risk of incident AD/ADRD (Figure 2). The adjusted hazard ratio (aHR) of AD/ADRD was 0.93 per SD longer in TL (95% CI 0.90–0.96, *p* = 3.37 × 10^−7^) equivalent to the effect of a 0.42‐year younger age (see Figure 2 and Table S9). Similar results were found for different *APOE* genotypes (see Figure 2 and Tables S10–S12), as well as for VD and AD equivalent to the effects of a 0.44‐year younger age and a 0.62‐year younger age, respectively (see Figure 2 and Tables S13, S14). Results for all the models associated with Figure 2, including covariates' associations, are presented in Tables S9–S14. TL alone (HR = 0.75, 95% CI 0.73–0.77) gave the K index 0.58 for AD/ADRD versus 0.59 from *APOE* genotype alone (HR [e3e4 vs. e3e3] = 2.29, 95% CI 2.17–2.42; HR [e4e4 vs. e3e3] = 6.29, 95% CI 5.78–6.85; HR [e2e4 vs. e3e3] = 1.40, 95% CI 1.19–1.64; HR [e2e3 vs. e3e3] = 0.85, 95% CI 0.77–0.93; HR [e2e2 vs. e3e3] = 1.04, 95% CI 0.73–1.49; HR [e1e2 vs. e3e3] not reported due to enormous uncertainty from a low number *n* = 3). Neither TL nor *APOE* genotype was discriminative as age (aHR = 1.19 per year increase) and sex (aHR = 1.34 comparing males to females) (K index = 0.78). The K index associated with age and sex (age‐and‐sex model) minimally increased after including TL (aHR = 0.93, 95% CI 0.90–0.96), *APOE* genotype (aHR [e3e4 vs. e3e3] = 2.38, 95% CI 2.25–2.53; aHR [e4e4 vs. e3e3] = 7.07, 95% CI 6.45–7.74; aHR [e2e4 vs. e3e3] = 1.50, 95% CI 1.27–1.78; aHR [e2e3 vs. e3e3] = 0.82, 95% CI 0.74–0.92; aHR [e2e2 vs. e3e3] = 0.96, 95% CI 0.64–1.45), and other covariates (Table S9) in the full model (K index = 0.80). For the subtypes of AD/ADRD, TL (HR = 0.75, 95% CI 0.71–0.79) and *APOE* genotype (HR [e3e4 vs. e3e3] = 4.00, 95% CI 3.51–4.55; HR [e4e4 vs. e3e3] = 13.33, 95% CI 11.19–15.89; HR [e2e4 vs. e3e3] = 1.60, 95% CI 1.06–2.39; HR [e2e3 vs. e3e3] = 0.70, 95% CI 0.52–0.93; HR [e2e2 vs. e3e3] = 0.75, 95% CI 0.24–2.35) produced the K indexes, 0.58 and 0.64, for AD only, relative to 0.81 from the age‐and‐sex model and 0.83 from the full model (Table S13). Similarly, TL (HR = 0.69, 95% CI 0.63–0.75) and *APOE* genotype (HR [e3e4 vs. e3e3] = 2.09, 95% CI 1.74–2.50; HR [e4e4 vs. e3e3] = 5.42, 95% CI 4.05–7.25; HR [e2e4vs. e3e3] = 1.61, 95% CI 1.00–2.59; HR [e2e3 vs. e3e3] = 0.81, 95% CI 0.59–1.12; HR [e2e2 vs. e3e3] = 1.05, 95% CI 0.34–3.28) produced the K indexes, 0.60 and 0.59, for VD only, relative to 0.80 from the age‐and‐sex model and 0.83 from the full model (Table S14). Overall, while the effect size of TL or *APOE* genotype is strong and remains statistically significant after adjusting for covariates, the associated discriminative power for AD/ADRD is limited and TL, *APOE* genotype, plus other covariates add minimal additional power to that of age and sex combined. The results above imply that the individual risk differences are still largely unexplained after considering TL, *APOE* genotype, demographics, socioeconomic and lifestyle factors.

**FIGURE 3:**
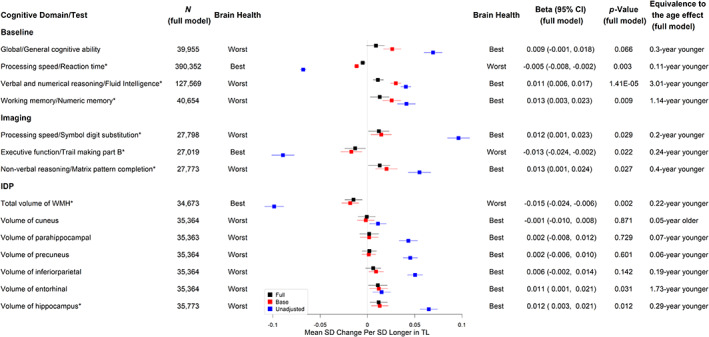
Associations of telomere length with cognitive function and brain imaging derived phenotypes. *Significant at the false discovery rate <0.05 level; N (full model): sample size; Full: linear regression models adjusting for age, sex, education, Townsend deprivation index, BMI, smoking status, alcohol intake frequency, IPAQ physical activity group, *APOE* genotype, PC1‐PC10, and baseline assessment center; Base: linear regression models adjusting for age and sex only; Unadjusted: linear regression models with no adjustment for covariates; Head size was included in each of the models for IDPs; *p*‐Value: unadjusted p‐value for multiple testing; The two ends representing best and worst brain health are labelled.

Longer TL was significantly associated with better cognitive performance at baseline, including faster reaction time (adjusted standardized *β* [a*β*] per SD longer in TL = −0.005, 95% CI ‐0.008 to −0.002, *p* = 0.003, equivalent to the age effect of a 0.11‐year younger age, see Figure 3 and Table S16), higher fluid intelligence (a*β* per SD = 0.011, 95% CI 0.006–0.017, *p* = 1.41 × 10^−5^, equivalent to the age effect of a 3.01‐year younger age, see Figure 3 and Table S17), and higher numeric memory (a*β* per SD = 0.013, 95% CI 0.003–0.023, *p* = 0.009, equivalent of the age effect of a 1.14‐year younger age, see Figure 3 and Table S18). Although not statistically significant at the level of FDR <5%, longer TL was suggestive of higher general cognitive ability (a*β* per SD = 0.009, 95% CI ‐0.001 to 0.018, *p* = 0.066, equivalent to a 0.30‐year younger age, see Figure 3 and Table S15) (Figure 3). For cognitive tests measured at the first imaging visit, each SD increase in TL was associated with better scores on tests of processing speed/executive functioning, including more symbol digit substitution (a*β* per SD = 0.012, 95% CI 0.001–0.023, *p* = 0.029, equivalent to the age effect of a 0.20‐year younger age, see Figure 3 and Table S19) and shorter duration to complete trail making part B (a*β* per SD = −0.013, 95% CI ‐0.024 to −0.002, *p* = 0.022, equivalent to the age effect of a 0.24‐year younger age, see Figure 3 and Table S20). Additionally, each SD increase in TL was associated with better non‐verbal reasoning measured with matrix pattern completion (a*β* per SD = 0.013, 95% CI 0.001–0.024, *p* = 0.027, equivalent to the age effect of a 0.40‐year younger age, see Figure 3 and Table S21).

**FIGURE 4:**
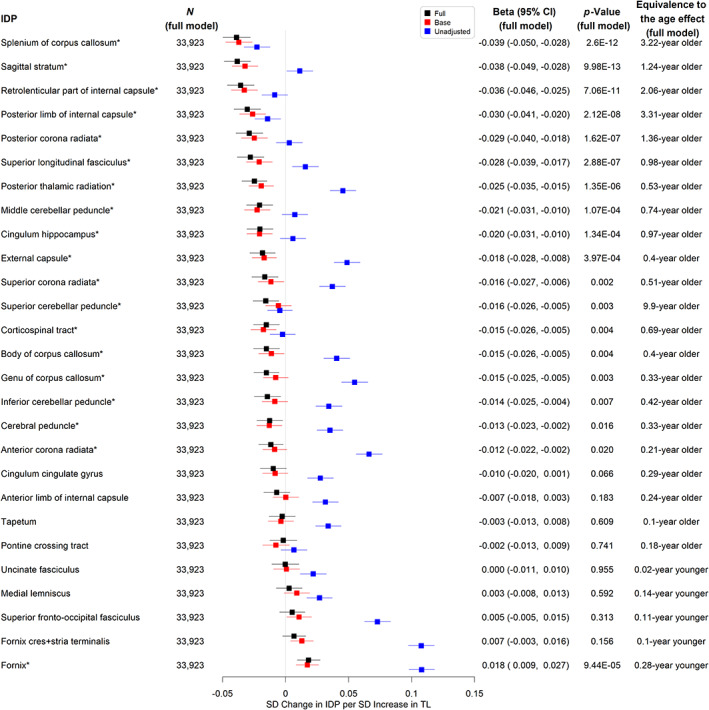
Associations between telomere length and weighted‐mean fractional anisotropy imaging derived phenotypes. *Significant at the false discovery rate <0.05 level; N (full model): sample size; Full: linear regression models adjusting for age, sex, education, Townsend deprivation index, BMI, smoking status, alcohol intake frequency, IPAQ physical activity group, *APOE* genotype, PC1‐PC10, baseline assessment center, and head size; Base: linear regression models adjusting for age, sex, and head size only; Unadjusted: linear regression models adjusting for head size only; *p*‐Value.

**FIGURE 5:**
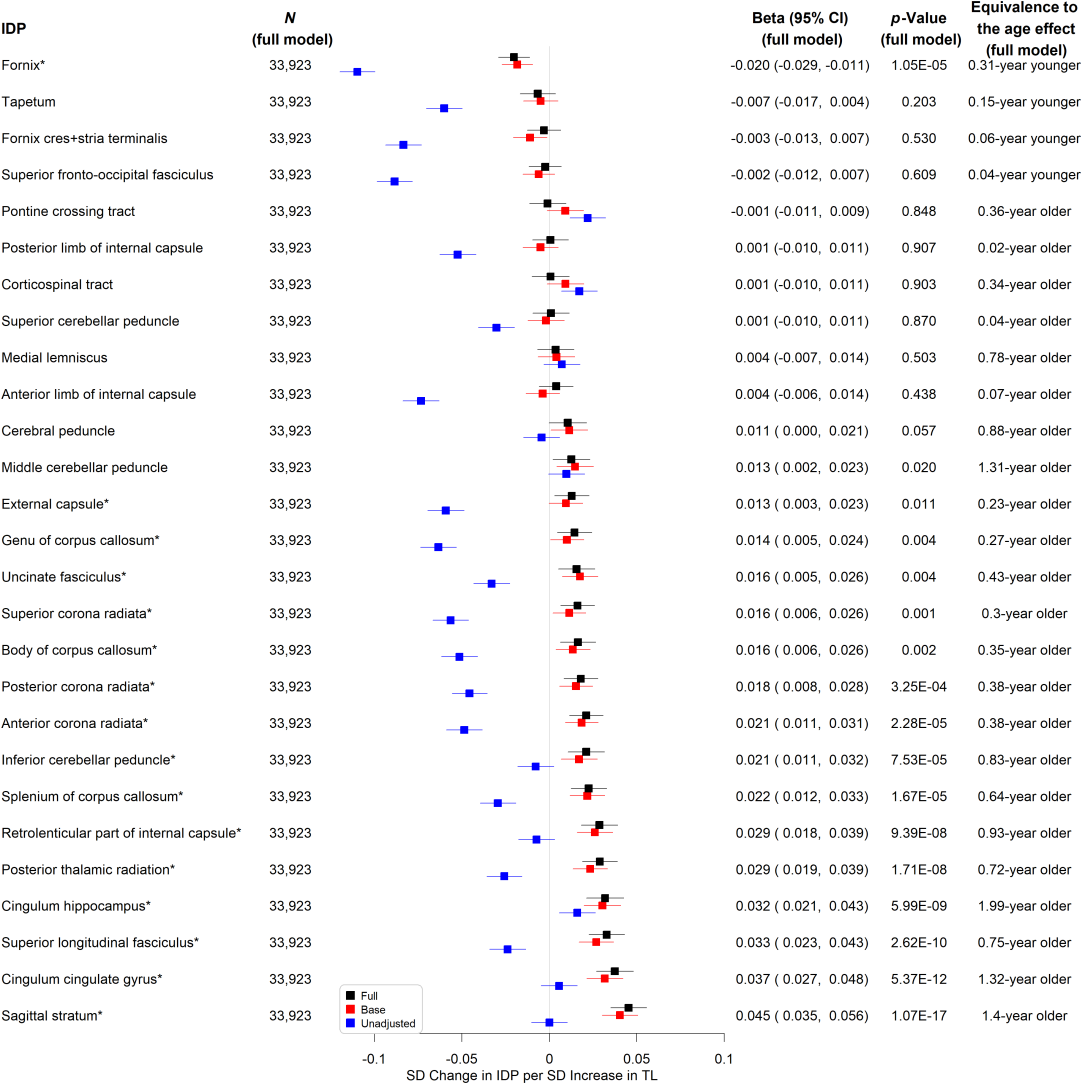
Associations between telomere length and weighted‐mean mean diffusivity imaging derived phenotypes. *Significant at the false discovery rate <0.05 level; N (full model): sample size; Full: linear regression models adjusting for age, sex, education, Townsend deprivation index, BMI, smoking status, alcohol intake frequency, IPAQ physical activity group, *APOE* genotype, PC1‐PC10, baseline assessment center, and head size; Base: linear regression models adjusting for age, sex, and head size only; Unadjusted: linear regression models adjusting for head size only; *p*‐value: unadjusted p‐value for multiple testing.

Longer TL was significantly associated with higher hippocampus volume (a*β* per SD = 0.012, 95% CI 0.003–0.021, *p* = 0.012, equivalent to the age effect of a 0.29‐year younger age, see Figure 3 and Table S28) and lower total WMH volume (a*β* per SD = −0.015, 95% CI ‐0.024 to −0.006, equivalent to the age effect of a 0.22‐year younger age, *p* = 0.002, see Figure 3 and Table S22). There were no significant associations between TL and other AD signature volumes (see Figure 3 and Tables S23–S27). We also evaluated the associations between TL and markers of white matter microstructural damage assessed by diffusion tensor imaging. Longer TL was associated with higher FA (indicating better white matter fiber integrity) and lower MD (indicating more glial cellularity and lower inflammatory burden) in the fornix. Interestingly, most of the other white matter tracts analyzed showed the opposite direction of association between TL and FA (Figure 4 and Tables S29‐S55) or MD (Figure 5 and Tables S56–S82).

### 3.2 Mendelian randomization analysis

A single instrument (rs35671754) was dropped from the analysis after winner's curse correction due to its sign reversing. The corrected effect sizes and standard errors were presented in Table S1. Our instrument set remained strong after winner's curse correction (mean F‐statistic 127.11 [before] versus 120.25 [after]; percent of the variance explained: 3.61% [before] versus 3.42% [after]). There was a low risk of violating the NOME assumption of MR‐Egger, with the I^2^ statistic close to 1 before (97.6%) and after (97.4%) winner's curse adjustment.

Using the IVW MR method, there was no evidence showing that genetically determined TL was associated with AD/ADRD or its subtypes (Figure S2). Genetically determined TL was not associated with any of the cognitive measures (Figure S3), volumetric IDPs of AD signatures, or total volume of WMH (Figure S4). Genetically determined TL was not associated with FA or MD in the fornix. The MR analysis, however, confirmed observational associations of longer TL with lower FA (Figure S5), and higher MD (Figure S6) in several tracts. Different MR methods showed consistent results (Figures S7–S11). MR‐Egger tended to produce a larger effect size than the other methods, which may be inflated by horizontal pleiotropy (indicated by an estimated intercept significantly different from zero; see Figures S12–S82). MR‐RAPS accounted for pleiotropy and the results of IVW and MR‐RAPS were similar across outcomes. All the numerical MR results are presented in Table S83.

## 4 DISCUSSION

Using middle‐aged adults with a mean follow‐up of 12.2 years, we found that longer leukocyte TL was associated with a lower risk of AD/ADRD, and specifically of AD and VD. In the population without dementia diagnoses, longer TL was associated with better cognitive performance. We also found that longer TL was associated with brain MRI features linked to a lower risk of dementia, including higher hippocampus volume, lower total volume of WMH, along with higher FA and lower MD in the fornix.

Our results are consistent with previous observational studies suggesting the associations of longer TL with AD (Forero et al., 2016) and risk factors of vascular dementia (Hinterberger et al., 2017). Longer TL also was associated with a lower risk of AD/ADRD across *APOE* genotypes (e3e3, e2, or e4). In contrast, a Sweden study (Hackenhaar et al., 2021) showed the association between short TL (first tertile) and a higher risk of AD in *APOE* non‐e4 carriers only. To fairly compare the results between studies, we performed an additional analysis of the association between TL in tertiles (short, medium [reference], and long) and incident AD for *APOE* e4 (e3e4 or e4e4) and non‐e4 carriers (e3e3, e2e3, or e3e3). Long TL was associated with a lower risk of incident AD in *APOE* e4 carriers (HR = 0.75, 95% CI 0.61–0.92, *p* = 0.005). We did not observe a statistically significant association between short TL and incident AD in *APOE* e4 carriers. Among *APOE* non‐e4 carriers, short or long TL was not significantly associated with incident AD. Both studies, however, were underpowered for small to moderate effect sizes. Other differences between studies may explain the inconsistent findings such as follow‐up time (25 years (Sweden) versus 12 years (UKB)) and AD diagnosis (clinical assessments versus ICD codes).

We did not find significant genetic associations of TL with AD/ADRD or its subtypes. Previous MR studies (Yu et al., 2021; Zhan et al., 2015) showed that genetically determined longer TL is associated with a lower risk of AD. These studies, however, focused only on AD and used preexisting data from meta‐GWAS that combined results from heterogeneous cohorts for TL and for AD, where the status of AD was analyzed instead of time to the first diagnosis of AD. In contrast, our samples were younger and we used more genetic instruments of TL to increase statistical power. We conducted a post hoc power analysis (https://shiny.cnsgenomics.com/mRnd/) (Brion et al., 2013) assuming an odds ratio (OR) for AD/ADRD associated with TL, which is similar to the HR in this study due to low incidence of AD/ADRD. The sample size of 435,043 including 6424 AD/ADRD cases (see Figure S2) provides 84% power to reject the null hypothesis (OR = 1) at 5% significance level when the actual OR for AD/ADRD is 0.8 per SD longer in TL (expected to be protective based on the observational association) using the genetic instruments of TL that explain 3.42% of variance in TL (see Table S1). However, the power significantly drops to 0.31 when the actual OR is only 0.9. The actual effect size is likely modest, and the power is not sufficient to reject the null hypothesis. As more AD/ADRD cases are diagnosed in UKB, further investigation of the association will be feasible. Future studies may improve the MR experiment by including other potential modifiable exposures in the path of TL to AD/ADRD and exploring the possibility of interactions between TL and covariates on AD/ADRD.

The relationship we observed between TL and alterations in the fornix is commensurate with the study by Staffaroni et al. (Staffaroni et al., 2018) that showed TL attrition over time was associated with decreased fornix FA, increased fornix MD, and greater hippocampal volume loss. The fornix is a white matter bundle in the limbic system that functions as the principal outflow pathway from the hippocampus (Oishi et al., 2014). Microstructural changes in the fornix had been reported as an early predictor of cognitive decline in older adults with normal cognition and an indicator of AD progression (Fletcher et al., 2013). Moreover, reduced FA and increased MD in the fornix have been suggested as promising imaging markers for AD (Oishi et al., 2014). Our findings on associations of longer TL with lower FA and higher MD in several white matter tracts suggest that longer TL may also have deleterious effects on related health outcomes including dementia.

The mechanism linking leukocyte TL with AD/ADRD and related brain MRI markers is unclear. Telomere dysfunction has a major impact on stem cell exhaustion and genomic instability, where the biological changes including reduced neurogenesis and increased mosaic DNA content variation have been lined to AD (Arendt et al., 2015; Cosacak et al., 2020; Lidzbarsky et al., 2018). Additionally, shorter TL may reflect an increased pool of senescent cells, which is associated with SASP (Ovadya & Krizhanovsky, 2014). Neuroinflammation plays an important role in exacerbating amyloid‐β burden and tau hyperphosphorylation that are two core pathologies of AD (Kinney et al., 2018). Pro‐inflammatory cytokines and other SASP factors are associated with brain structural abnormalities, cerebrovascular pathology, cognitive impairment, and elevated risk of AD (Diniz et al., 2010; Wallin et al., 2017). Future studies are necessary to address by which mechanisms TL can have protective or harmful effects.

Several limitations need to be considered when interpreting the results of the current study. *First*, TL was measured in leukocytes in peripheral blood rather than in the brain. However, peripheral blood TL is positively correlated with cerebellum TL, therefore, can be used to reveal the relationship between cerebellum TL and AD (Lukens et al., 2009). *Second*, study participants were relatively young, with many not yet old enough to have developed AD/ADRD. *Third*, our results may not be generalized to non‐European populations. *Fourth*, since TL was only measured at baseline, we could not assess the association between TL attrition over time and the risk of dementia or its related measures. We also did not have data to link baseline TL to the progression towards AD/ADRD using imaging data or cognitive test results. *Fifth*, healthy volunteers are over‐represented in our baseline and imaging cohorts, which could lead to underestimated exposure‐outcome associations but the impact is alleviated by significant heterogeneity of exposures (Fry et al., 2017). *Lastly*, TL is influenced by a complex interaction of genetic, lifestyle, and environmental factors. While MR analysis is robust to confounding and reverse causation, TL not determined by the genetic instruments is not modeled in the MR analysis so the results may not apply to observed TL (Pudas et al., 2021). Overall, we believe that the above limitations would diminish rather than enhance our ability to reject the null hypothesis, thus raising the robustness of our statistically significant findings.

Together with our findings that longer TL is associated with better cognitive performance and lower risks of incident AD, VD, and their related brain markers, this suggests that TL is a robust indicator of neurodegeneration or cognitive impairment toward the development of AD/ADRD. Further research is needed to elucidate the biological mechanisms linking TL and dementia and to understand the health impact of lower FA and higher MD associated with longer TL in several white matter tracts.

## AUTHOR CONTRIBUTIONS

C.L.K., L.C.P, D.M., L.W., D.C.S., and R.L. designed the study; M.X. and C.L.K. performed statistical analyses; J.B. guided Mendelian randomization analyses and developed the method to adjust for the winner's curse; R.L. and C.L.K drafted the initial manuscript, and all the authors reviewed the manuscript.

## CONFLICT OF INTEREST STATEMENT

The authors have no conflicts of interest to disclose.

## Supporting information


FiguresS1‐S82

TableS1

TablesS2‐S8

TablesS9‐S14

TablesS15‐S28

TablesS29‐S55

TablesS56‐S82

TableS83

TextsS1‐S3

## Data Availability

Data used in this research can be requested via an application to UK Biobank.
