# Supplementary material for: Mid‐life leukocyte telomere length and dementia risk: An observational and mendelian randomization study of 435,046 UK Biobank participants
Source: Aging Cell. 2023 May 30;22(7):e13808. doi: 10.1111/acel.13808 (PMC10352557; doi:10.1111/acel.13808)
Supplement: Supplementary file 1 — FiguresS1‐S82 [file ACEL-22-e13808-s002.docx]

**Figures S1-S82**

**Mid-life leukocyte telomere length and dementia risk: an observational and Mendelian randomization study of 435,046 UK Biobank participants**

**Figure S1** | Cumulative incident risk of AD/ADRD over time for different quintile groups of TL

**Figure S2** | Associations between genetically determined telomere length and AD/ADRD using the Inverse Variance Weighted (IVW) method

**Figure S3** | Associations between genetically determined telomere length and cognitive function using the Inverse Variance Weighted (IVW) method

**Figure S4** | Associations between genetically determined telomere length and volumetric IDPs of AD signatures and white matter hyperintensities (WMH) using the Inverse Variance Weighted (IVW) method

**Figure S5** | Associations between genetically determined telomere length and weighted-mean fractional anisotropy IDPs using the Inverse Variance Weighted (IVW) method

**Figure S6** | Associations between genetically determined telomere length and weighted-mean mean diffusivity IDPs using the Inverse Variance Weighted (IVW) method

**Figure S7** | Associations between genetically determined telomere length and AD/ADRD, comparing the primary (IVW: inverse variance weighted) to secondary MR methods (Mendelian randomization robust adjusted profile score (MR-RAPS); weighted median based method; MR-Egger method)

**Figure S8** | Associations between genetically determined telomere length and cognitive function, comparing the primary (IVW: inverse variance weighted) to secondary MR methods (Mendelian randomization robust adjusted profile score (MR-RAPS); weighted median based method; MR-Egger method)

**Figure S9** | Associations between genetically determined telomere length and volumetric IDPs of AD signatures and white matter hyperintensities (WMH), comparing the primary (IVW: inverse variance weighted) to secondary MR methods (Mendelian randomization robust adjusted profile score (MR-RAPS); weighted median based method; MR-Egger method)

**Figure S10** | Associations between genetically determined telomere length and weighted-mean fractional anisotropy IDPs, comparing the primary (IVW: inverse variance weighted) to secondary MR methods (Mendelian randomization robust adjusted profile score (MR-RAPS); weighted median based method; MR-Egger method)

**Figure S11** | Associations between genetically determined telomere length and weighted-mean mean diffusivity IDPs, comparing the primary (IVW: inverse variance weighted) to secondary MR methods (Mendelian randomization robust adjusted profile score (MR-RAPS); weighted median based method; MR-Egger method)

**Figure S12** | SNP-AD/ADRD association plotted against SNP-telomere length association, labelled by the mapped gene, with MR slope estimates shown

**Figure S13** | SNP-AD association plotted against SNP-telomere length association, labelled by the mapped gene, with MR slope estimates shown

**Figure S14** | SNP-vascular dementia association plotted against SNP-telomere length association, labelled by the mapped gene, with MR slope estimates shown

**Figure S15** | SNP-general cognitive ability association plotted against SNP-telomere length association, labelled by the mapped gene, with MR slope estimates shown

**Figure S16** | SNP-reaction time association plotted against SNP-telomere length association, labelled by the mapped gene, with MR slope estimates shown

**Figure S17** | SNP-fluid intelligence association plotted against SNP-telomere length association, labelled by the mapped gene, with MR slope estimates shown

**Figure S18** | SNP-numeric memory association plotted against SNP-telomere length association, labelled by the mapped gene, with MR slope estimates shown

**Figure S19** | SNP-symbol digit substitution association plotted against SNP-telomere length association, labelled by the mapped gene, with MR slope estimates shown

**Figure S20** | SNP-trail making part B association plotted against SNP-telomere length association, labelled by the mapped gene, with MR slope estimates shown

**Figure S21** | SNP-matrix pattern completion association plotted against SNP-telomere length association, labelled by the mapped gene, with MR slope estimates shown

**Figure S22** | SNP-total volume of white matter hyperintensities association plotted against SNP-telomere length association, labelled by the mapped gene, with MR slope estimates shown

**Figure S23** | SNP-cuneus volume association plotted against SNP-telomere length association, labelled by the mapped gene, with MR slope estimates shown

**Figure S24** | SNP-entorhinal volume association plotted against SNP-telomere length association, labelled by the mapped gene, with MR slope estimates shown

**Figure S25** | SNP-inferiorparietal volume association plotted against SNP-telomere length association, labelled by the mapped gene, with MR slope estimates shown

**Figure S26** | SNP-parahippocampal volume association plotted against SNP-telomere length association, labelled by the mapped gene, with MR slope estimates shown

**Figure S27** | SNP-precuneus volume association plotted against SNP-telomere length association, labelled by the mapped gene, with MR slope estimates shown

**Figure S28** | SNP-hippocampus volume association plotted against SNP-telomere length association, labelled by the mapped gene, with MR slope estimates shown

**Figure S29** | SNP-mean FA in anterior corona radiata association plotted against SNP-telomere length association, labelled by the mapped gene, with MR slope estimates shown

**Figure S30** | SNP-mean FA in anterior limb of internal capsule association plotted against SNP-telomere length association, labelled by the mapped gene, with MR slope estimates shown

**Figure S31** | SNP-mean FA in body of corpus callosum association plotted against SNP-telomere length association, labelled by the mapped gene, with MR slope estimates shown

**Figure S32** | SNP-mean FA in cerebral peduncle association plotted against SNP-telomere length association, labelled by the mapped gene, with MR slope estimates shown

**Figure S33** | SNP-mean FA in cingulum cingulate gyrus association plotted against SNP-telomere length association, labelled by the mapped gene, with MR slope estimates shown

**Figure S34** | SNP-mean FA in cingulum hippocampus association plotted against SNP-telomere length association, labelled by the mapped gene, with MR slope estimates shown

**Figure S35** | SNP-mean FA in corticospinal tract association plotted against SNP-telomere length association, labelled by the mapped gene, with MR slope estimates shown

**Figure S36** | SNP-mean FA in external capsule association plotted against SNP-telomere length association, labelled by the mapped gene, with MR slope estimates shown

**Figure S37** | SNP-mean FA in fornix association plotted against SNP-telomere length association, labelled by the mapped gene, with MR slope estimates shown

**Figure S38** | SNP-mean FA in fornix cres+stria terminalis association plotted against SNP-telomere length association, labelled by the mapped gene, with MR slope estimates shown

**Figure S39** | SNP-mean FA in genu of corpus callosum association plotted against SNP-telomere length association, labelled by the mapped gene, with MR slope estimates shown

**Figure S40** | SNP-mean FA in inferior cerebellar peduncle association plotted against SNP-telomere length association, labelled by the mapped gene, with MR slope estimates shown

**Figure S41** | SNP-mean FA in medial lemniscus association plotted against SNP-telomere length association, labelled by the mapped gene, with MR slope estimates shown

**Figure S42** | SNP-mean FA in middle cerebellar peduncle association plotted against SNP-telomere length association, labelled by the mapped gene, with MR slope estimates shown

**Figure S43** | SNP-mean FA in pontine crossing tract association plotted against SNP-telomere length association, labelled by the mapped gene, with MR slope estimates shown

**Figure S44** | SNP-mean FA in posterior corona radiata association plotted against SNP-telomere length association, labelled by the mapped gene, with MR slope estimates shown

**Figure S45** | SNP-mean FA in posterior limb of internal capsule association plotted against SNP-telomere length association, labelled by the mapped gene, with MR slope estimates shown

**Figure S46** | SNP-mean FA in posterior thalamic radiation association plotted against SNP-telomere length association, labelled by the mapped gene, with MR slope estimates shown

**Figure S47** | SNP-mean FA in retrolenticular part of internal capsule association plotted against SNP-telomere length association, labelled by the mapped gene, with MR slope estimates shown

**Figure S48** | SNP-mean FA in sagittal stratum association plotted against SNP-telomere length association, labelled by the mapped gene, with MR slope estimates shown

**Figure S49** | SNP-mean FA in splenium of corpus callosum association plotted against SNP-telomere length association, labelled by the mapped gene, with MR slope estimates shown

**Figure S50** | SNP-mean FA in superior cerebellar peduncle association plotted against SNP-telomere length association, labelled by the mapped gene, with MR slope estimates shown

**Figure S51** | SNP-mean FA in superior corona radiata association plotted against SNP-telomere length association, labelled by the mapped gene, with MR slope estimates shown

**Figure S52** | SNP-mean FA in superior fronto-occipital fasciculus association plotted against SNP-telomere length association, labelled by the mapped gene, with MR slope estimates shown

**Figure S53** | SNP-mean FA in superior longitudinal fasciculus association plotted against SNP-telomere length association, labelled by the mapped gene, with MR slope estimates shown

**Figure S54** | SNP-mean FA in tapetum association plotted against SNP-telomere length association, labelled by the mapped gene, with MR slope estimates shown

**Figure S55** | SNP-mean FA in uncinate fasciculus association plotted against SNP-telomere length association, labelled by the mapped gene, with MR slope estimates shown

**Figure S56** | SNP-mean MD in anterior corona radiata association plotted against SNP-telomere length association, labelled by the mapped gene, with MR slope estimates shown

**Figure S57** | SNP-mean MD in anterior limb of internal capsule association plotted against SNP-telomere length association, labelled by the mapped gene, with MR slope estimates shown

**Figure S58** | SNP-mean MD in body of corpus callosum association plotted against SNP-telomere length association, labelled by the mapped gene, with MR slope estimates shown

**Figure S59** | SNP-mean MD in cerebral peduncle association plotted against SNP-telomere length association, labelled by the mapped gene, with MR slope estimates shown

**Figure S60** | SNP-mean MD in cingulum cingulate gyrus association plotted against SNP-telomere length association, labelled by the mapped gene, with MR slope estimates shown

**Figure S61** | SNP-mean MD in cingulum hippocampus association plotted against SNP-telomere length association, labelled by the mapped gene, with MR slope estimates shown

**Figure S62** | SNP-mean MD in corticospinal tract association plotted against SNP-telomere length association, labelled by the mapped gene, with MR slope estimates shown

**Figure S63** | SNP-mean MD in external capsule association plotted against SNP-telomere length association, labelled by the mapped gene, with MR slope estimates shown

**Figure S64** | SNP-mean MD in fornix association plotted against SNP-telomere length association, labelled by the mapped gene, with MR slope estimates shown

**Figure S65** | SNP-mean MD in fornix cres+stria terminalis association plotted against SNP-telomere length association, labelled by the mapped gene, with MR slope estimates shown

**Figure S66** | SNP-mean MD in genu of corpus callosum association plotted against SNP-telomere length association, labelled by the mapped gene, with MR slope estimates shown

**Figure S67** | SNP-mean MD in inferior cerebellar peduncle association plotted against SNP-telomere length association, labelled by the mapped gene, with MR slope estimates shown

**Figure S68** | SNP-mean MD in medial lemniscus association plotted against SNP-telomere length association, labelled by the mapped gene, with MR slope estimates shown

**Figure S69** | SNP-mean MD in middle cerebellar peduncle association plotted against SNP-telomere length association, labelled by the mapped gene, with MR slope estimates shown

**Figure S70** | SNP-mean MD in pontine crossing tract association plotted against SNP-telomere length association, labelled by the mapped gene, with MR slope estimates shown

**Figure S71** | SNP-mean MD in posterior corona radiata association plotted against SNP-telomere length association, labelled by the mapped gene, with MR slope estimates shown

**Figure S72** | SNP-mean MD in posterior limb of internal capsule association plotted against SNP-telomere length association, labelled by the mapped gene, with MR slope estimates shown

**Figure S73** | SNP-mean MD in posterior thalamic radiation association plotted against SNP-telomere length association, labelled by the mapped gene, with MR slope estimates shown

**Figure S74** | SNP-mean MD in retrolenticular part of internal capsule association plotted against SNP-telomere length association, labelled by the mapped gene, with MR slope estimates shown

**Figure S75** | SNP-mean MD in sagittal stratum association plotted against SNP-telomere length association, labelled by the mapped gene, with MR slope estimates shown

**Figure S76** | SNP-mean MD in splenium of corpus callosum association plotted against SNP-telomere length association, labelled by the mapped gene, with MR slope estimates shown

**Figure S77** | SNP-mean MD in superior cerebellar peduncle association plotted against SNP-telomere length association, labelled by the mapped gene, with MR slope estimates shown

**Figure S78** | SNP-mean MD in superior corona radiata association plotted against SNP-telomere length association, labelled by the mapped gene, with MR slope estimates shown

**Figure S79** | SNP-mean MD in superior fronto-occipital fasciculus association plotted against SNP-telomere length association, labelled by the mapped gene, with MR slope estimates shown

**Figure S80** | SNP-mean MD in superior longitudinal fasciculus association plotted against SNP-telomere length association, labelled by the mapped gene, with MR slope estimates shown

**Figure S81** | SNP-mean MD in tapetum association plotted against SNP-telomere length association, labelled by the mapped gene, with MR slope estimates shown

**Figure S82** | SNP-mean MD in uncinate fasciculus association plotted against SNP-telomere length association, labelled by the mapped gene, with MR slope estimates shown


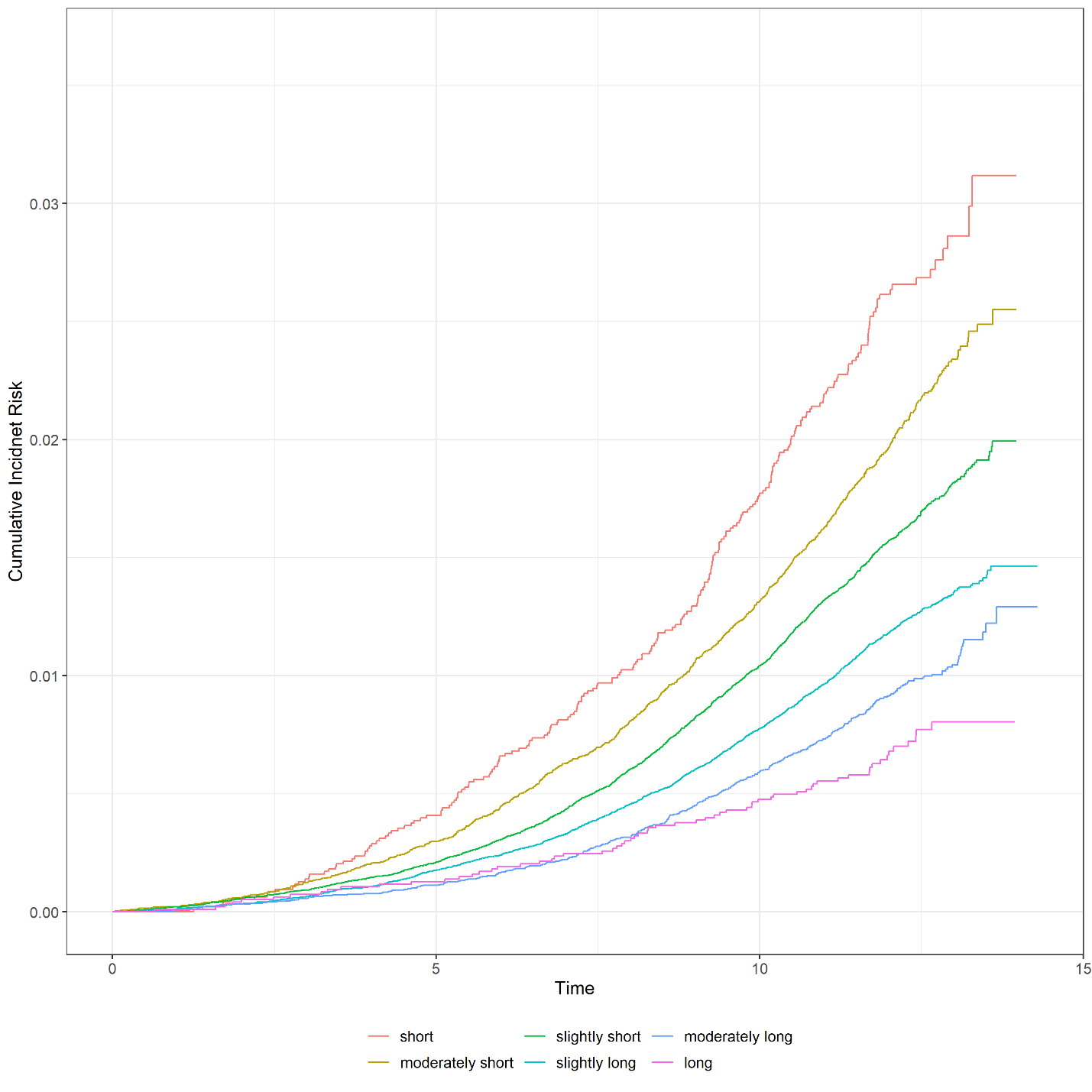


**Figure S1. Cumulative incident risk of AD/ADRD over time for the TL groups from the shortest to the longest based on the mean and standard deviation (SD) of TL: 1) short: (-Inf, mean-2SD]; 2) moderately short: (mean-2SD, mean-SD]; 3) slightly short: (mean-SD, mean]; 4) slightly long: (mean, mean+SD]; 5) moderately long: (mean+SD, mean+2SD]; and 6) long: (mean+2SD, Inf]**


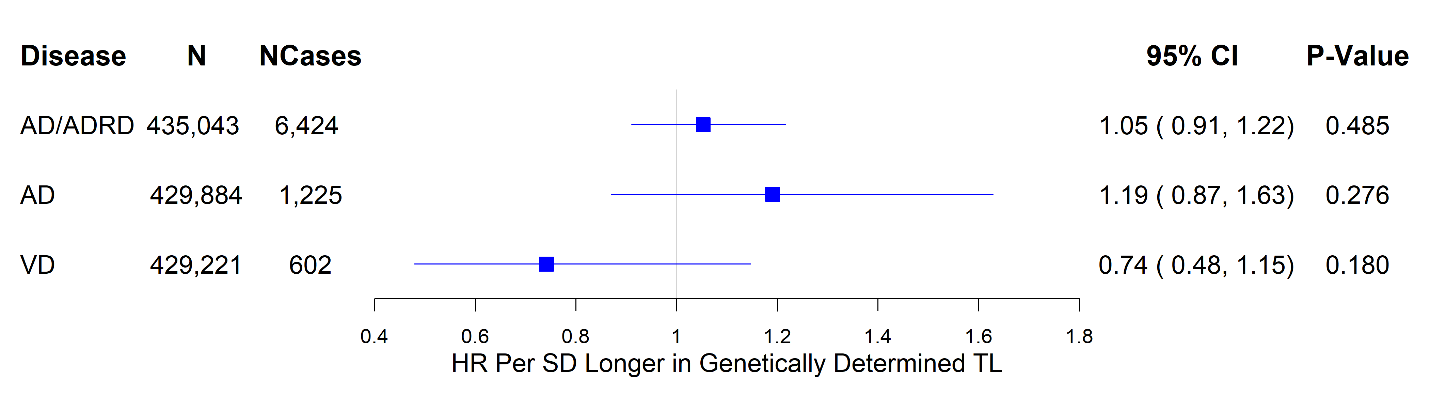


**Figure S2.** Associations between genetically determined telomere length and AD/ADRD using the Inverse Variance Weighted (IVW) method

1. *Significant at the false discovery rate < 0.05 level; AD: Alzheimer’s disease or dementia in Alzheimer’s disease; VD: vascular dementia.


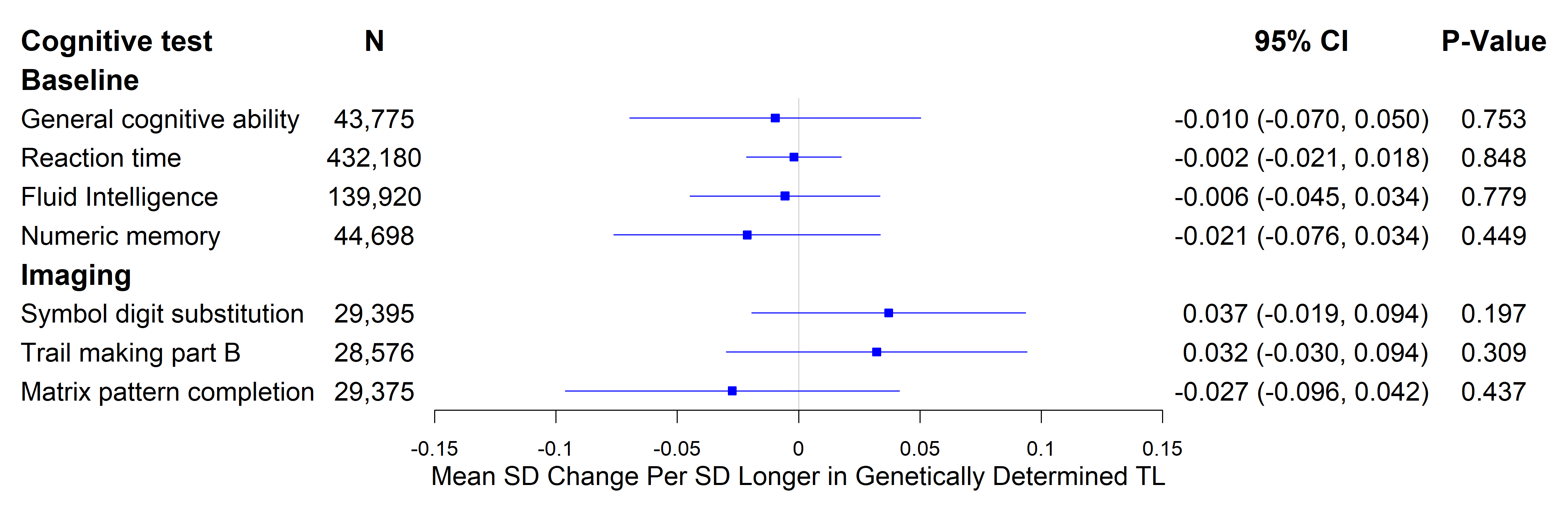


**Figure S3.** Associations between genetically determined telomere length and cognitive function using the Inverse Variance Weighted (IVW) method

1. *Significant at the false discovery rate < 0.05 level.


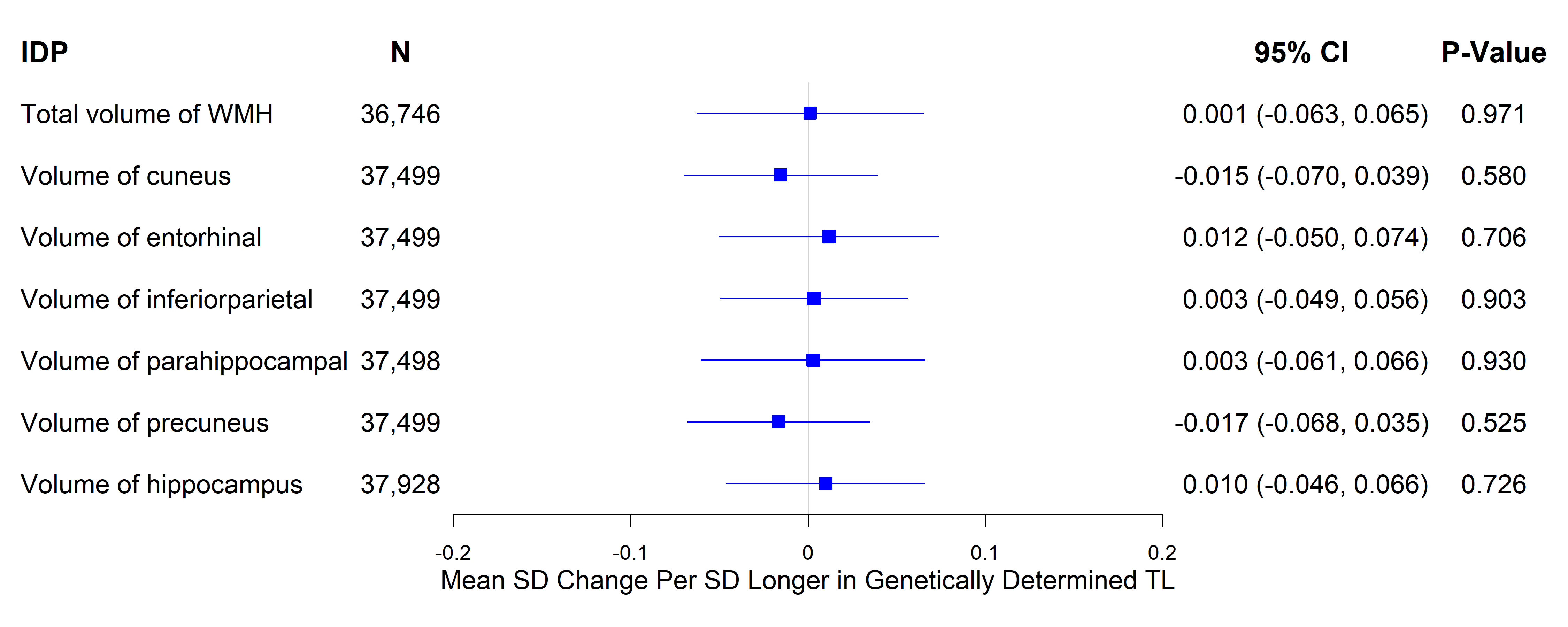


**Figure S4.** Associations between genetically determined telomere length and volumetric IDPs of AD signatures and white matter hyperintensities (WMH) using the Inverse Variance Weighted (IVW) method

1. *Significant at the false discovery rate < 0.05 level.

**
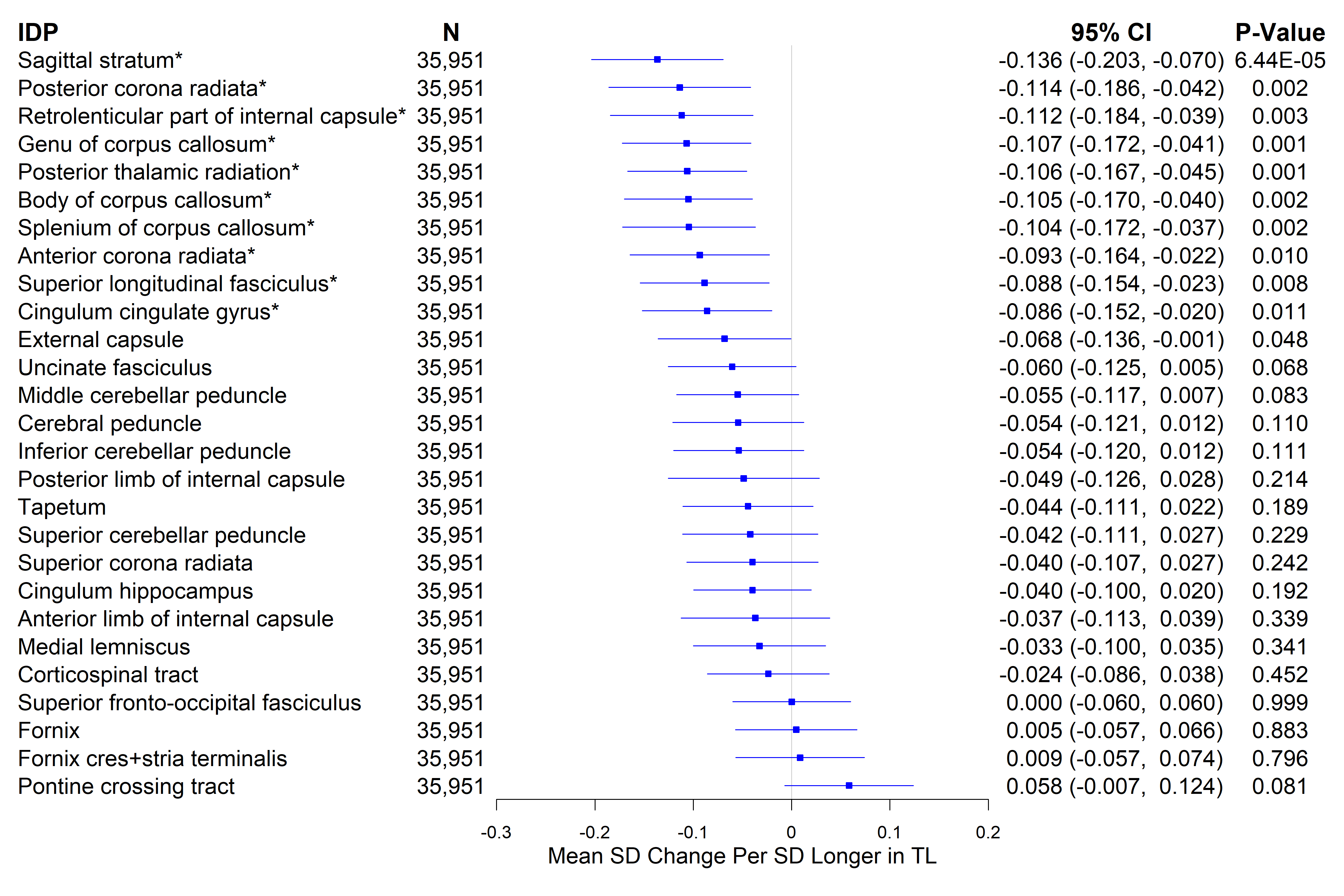
**

**Figure S5.** Associations between genetically determined telomere length and weighted-mean fractional anisotropy IDPs using the Inverse Variance Weighted (IVW) method

1. *Significant at the false discovery rate < 0.05 level.

**
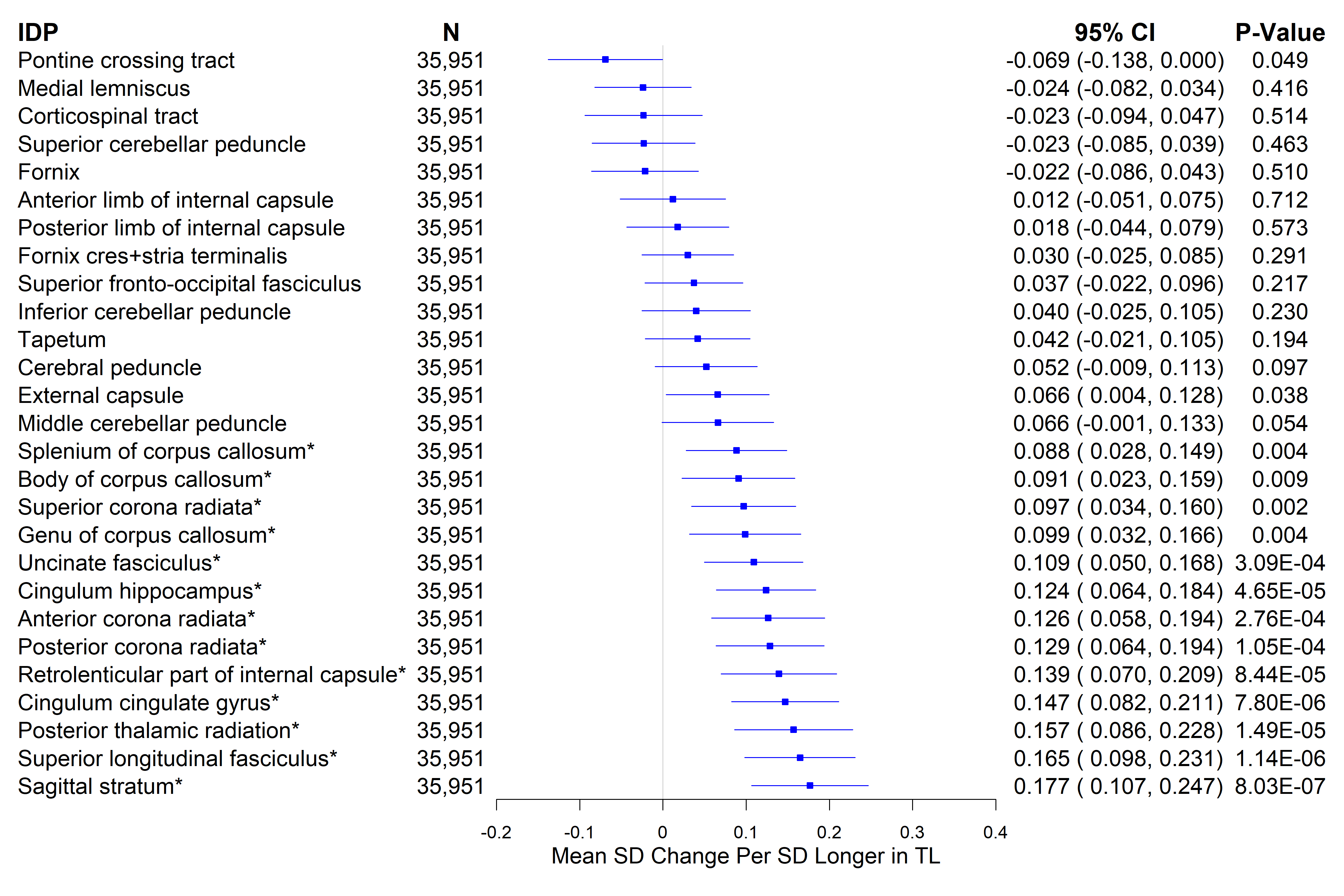
**

**Figure S6.** Associations between genetically determined telomere length and weighted-mean mean diffusivity IDPs using the Inverse Variance Weighted (IVW) method

1. *Significant at the false discovery rate < 0.05 level.

**
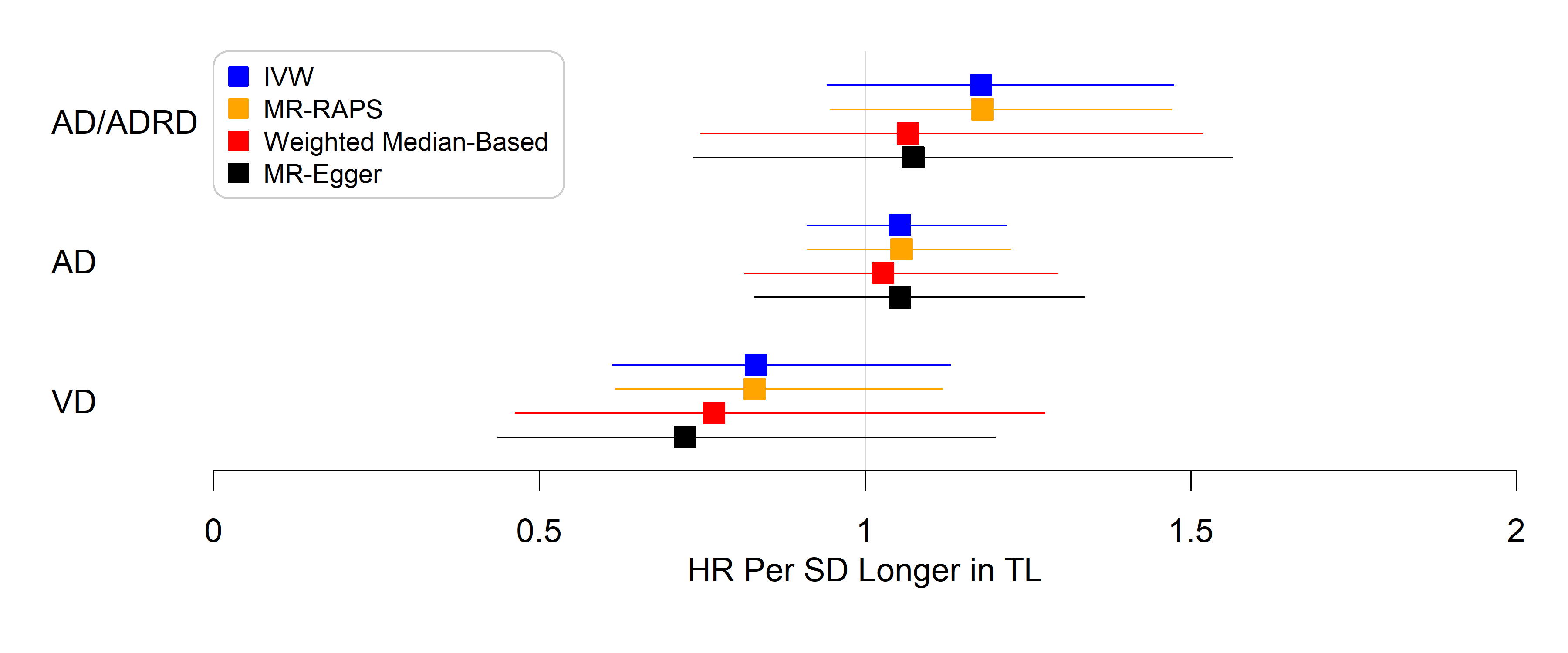
**

**Figure S7.** Associations between genetically determined telomere length and AD/ADRD, comparing the primary (IVW: inverse variance weighted) to secondary MR methods (Mendelian randomization robust adjusted profile score (MR-RAPS); weighted median based method; MR-Egger method)

1. *Significant at the false discovery rate < 0.05 level using the IVW method; AD: Alzheimer’s disease or dementia in Alzheimer’s disease; VD: vascular dementia.

**
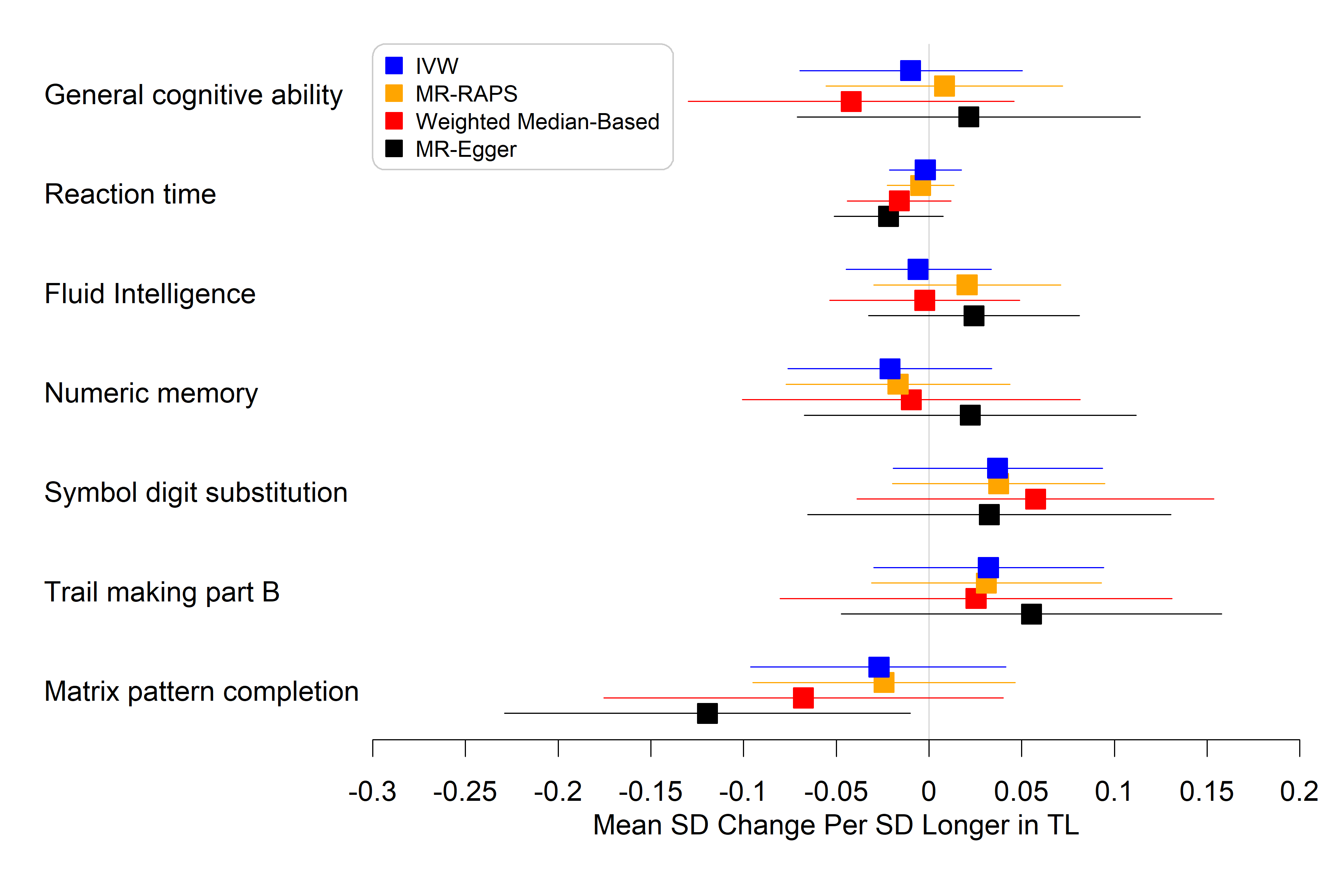
**

**Figure S8.** Associations between genetically determined telomere length and cognitive function, comparing the primary (IVW: inverse variance weighted) to secondary MR methods (Mendelian randomization robust adjusted profile score (MR-RAPS); weighted median based method; MR-Egger method)

1. *Significant at the false discovery rate < 0.05 level using the IVW method.


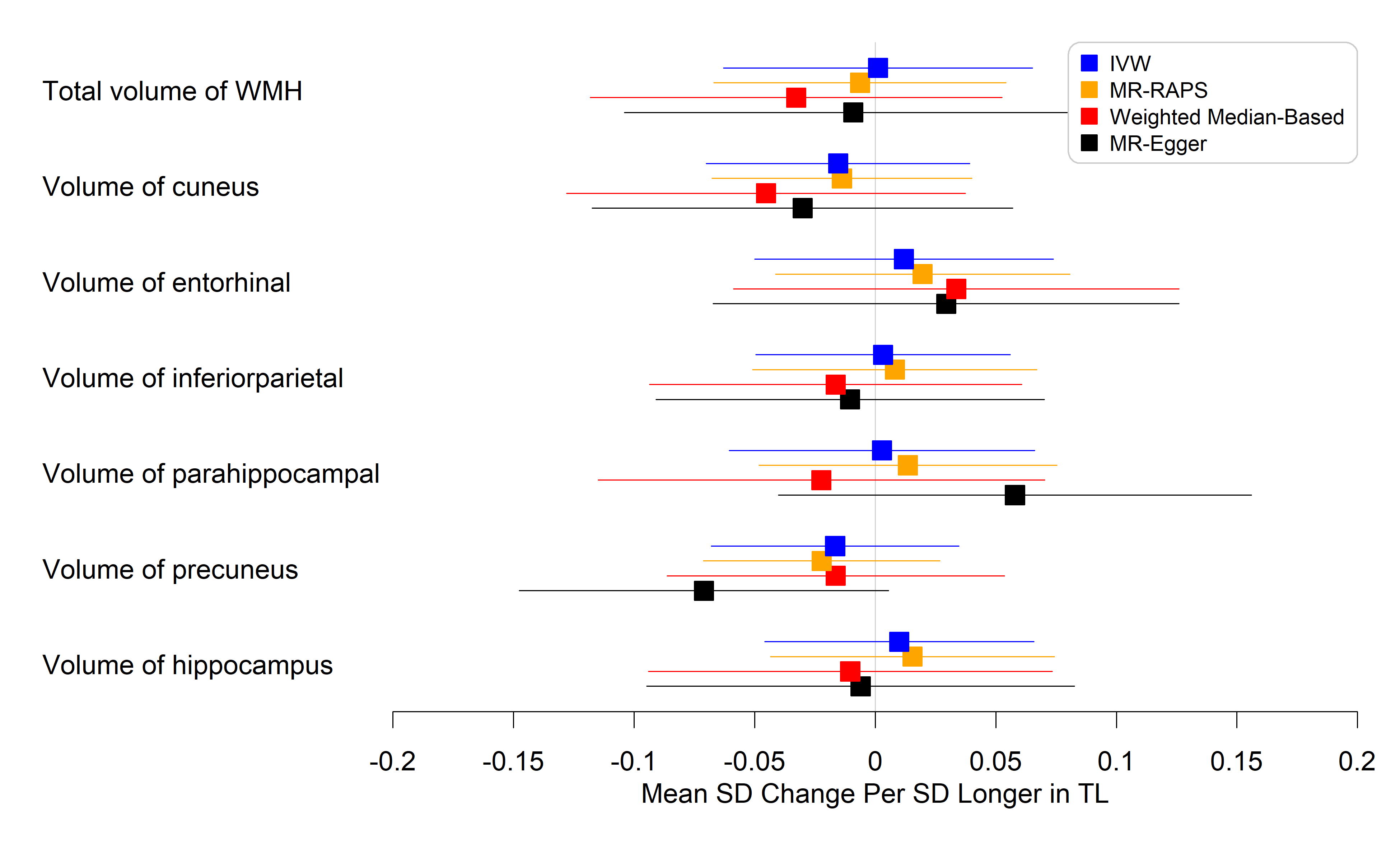


**Figure S9.** Associations between genetically determined telomere length and volumetric IDPs of AD signatures and white matter hyperintensities (WMH), comparing the primary (IVW: inverse variance weighted) to secondary MR methods (Mendelian randomization robust adjusted profile score (MR-RAPS); weighted median based method; MR-Egger method)

1. *Significant at the false discovery rate < 0.05 level using the IVW method.

**
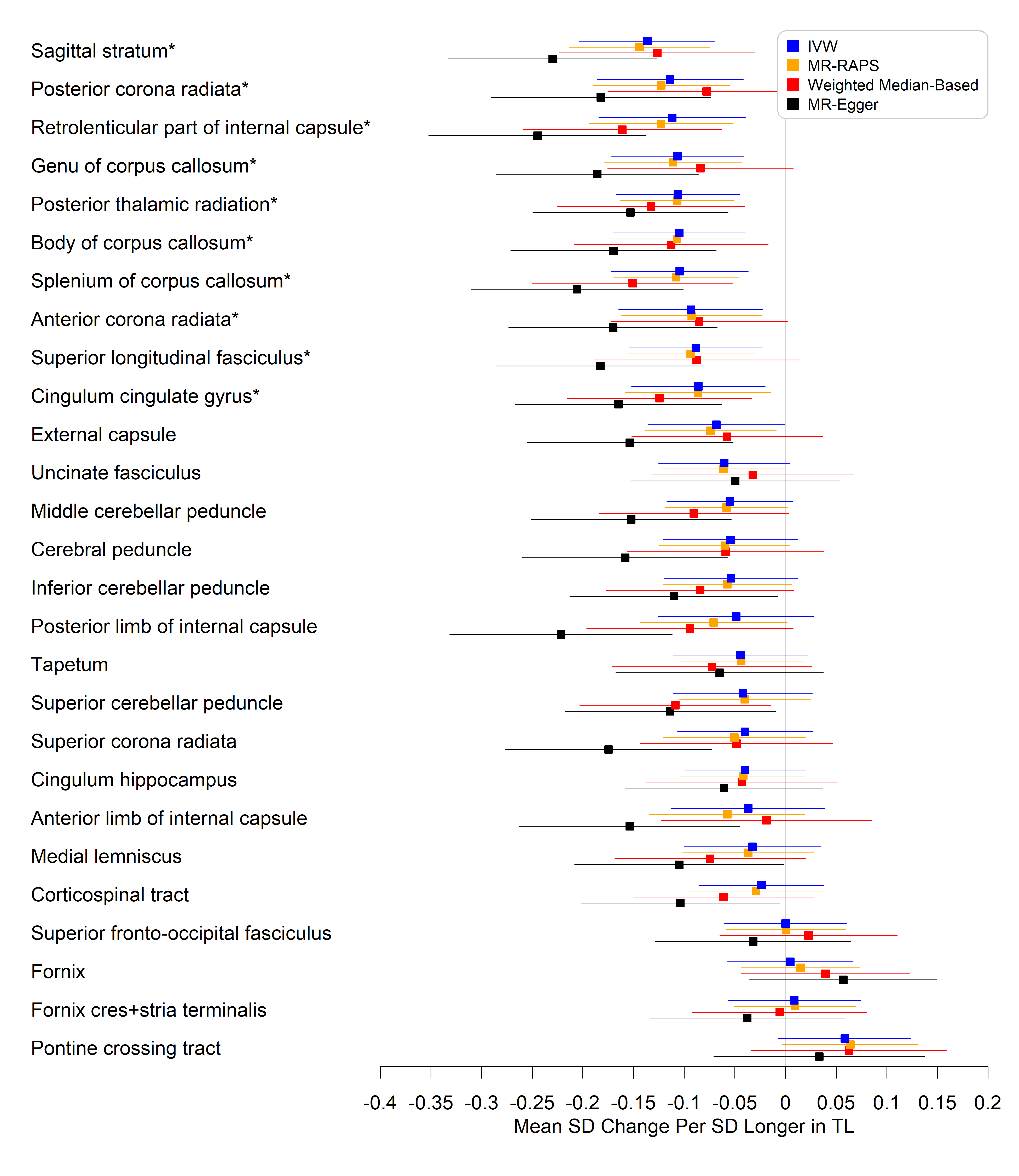
**

**Figure S10.** Associations between genetically determined telomere length and weighted-mean fractional anisotropy IDPs, comparing the primary (IVW: inverse variance weighted) to secondary MR methods (Mendelian randomization robust adjusted profile score (MR-RAPS); weighted median based method; MR-Egger method)

1. *Significant at the false discovery rate < 0.05 level using the IVW method.

**
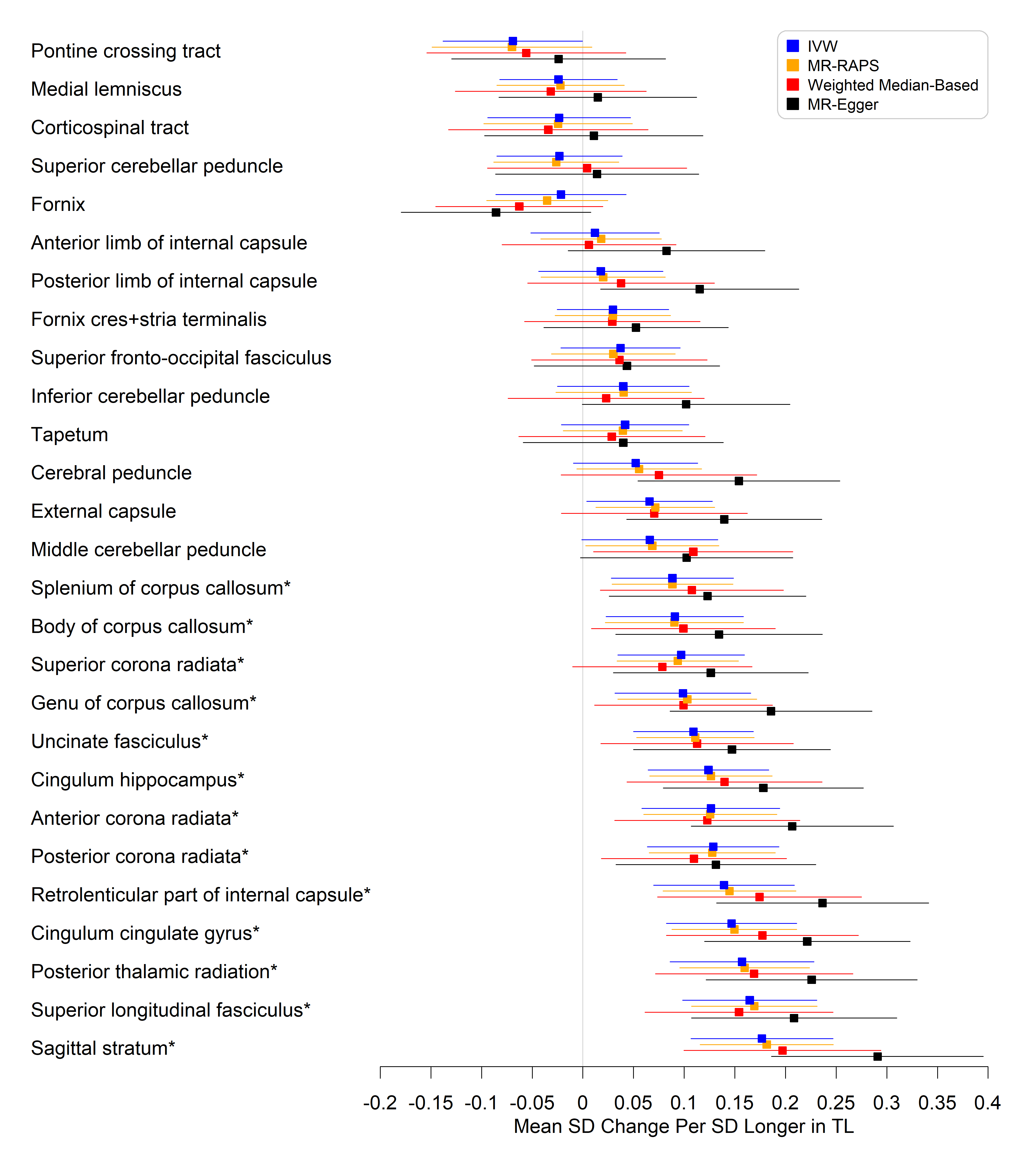
**

**Figure S11.** Associations between genetically determined telomere length and weighted-mean mean diffusivity IDPs, comparing the primary (IVW: inverse variance weighted) to secondary MR methods (Mendelian randomization robust adjusted profile score (MR-RAPS); weighted median based method; MR-Egger method)

1. *Significant at the false discovery rate < 0.05 level using the IVW method.

**
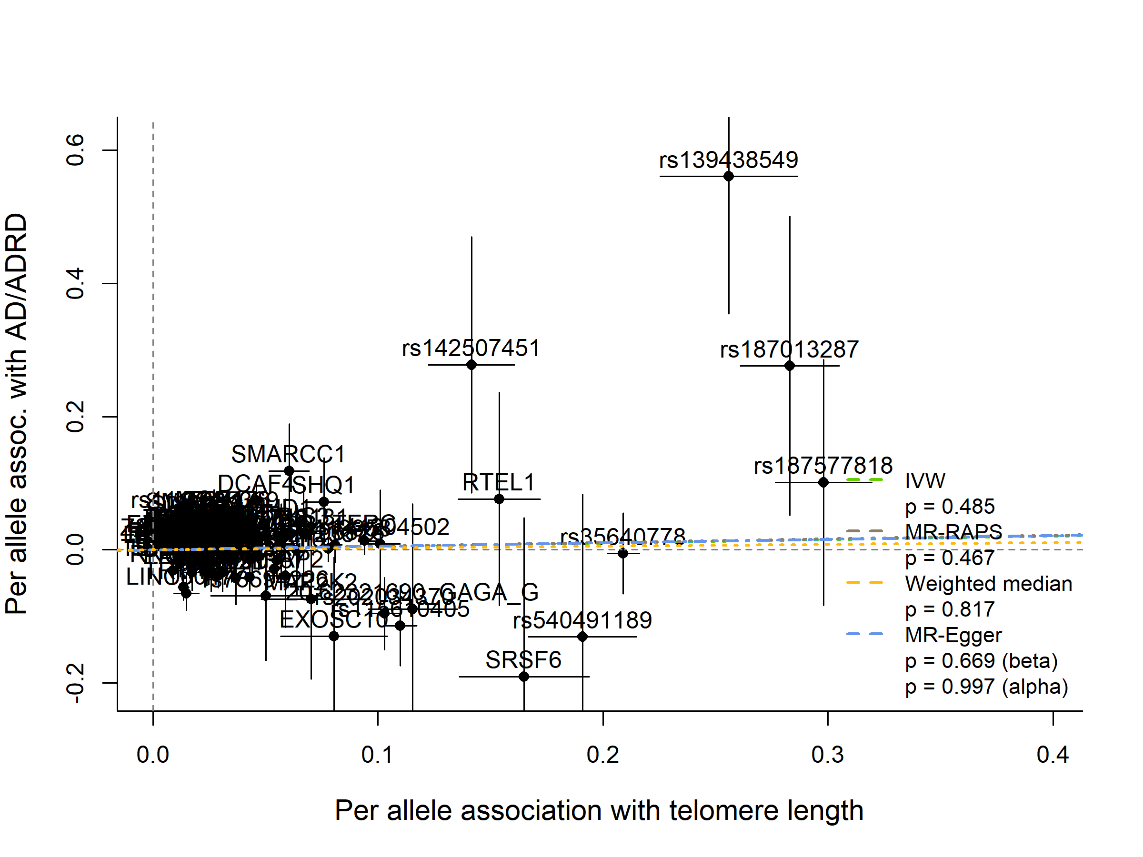
**

**Figure S12.** SNP-AD/ADRD association plotted against SNP-telomere length association, labelled by the mapped gene, with MR slope estimates shown


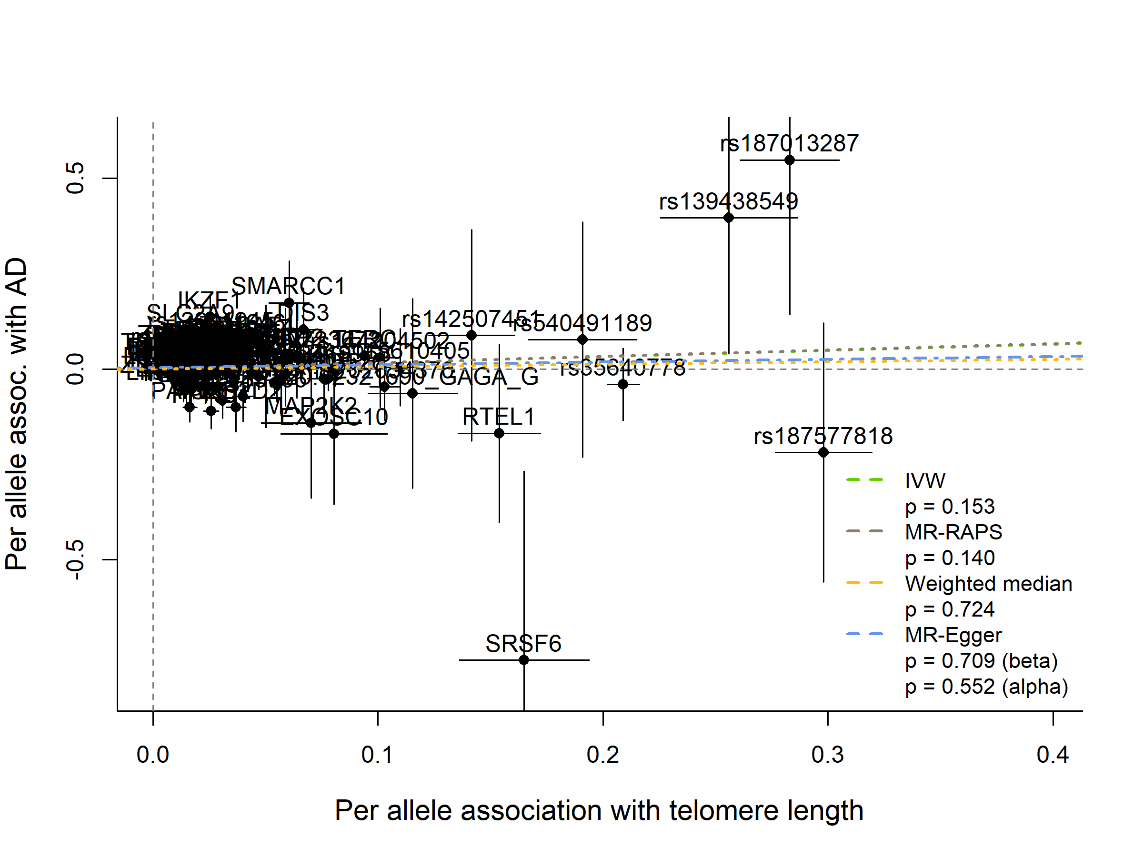


**Figure S13.** SNP-AD association plotted against SNP-telomere length association, labelled by the mapped gene, with MR slope estimates shown


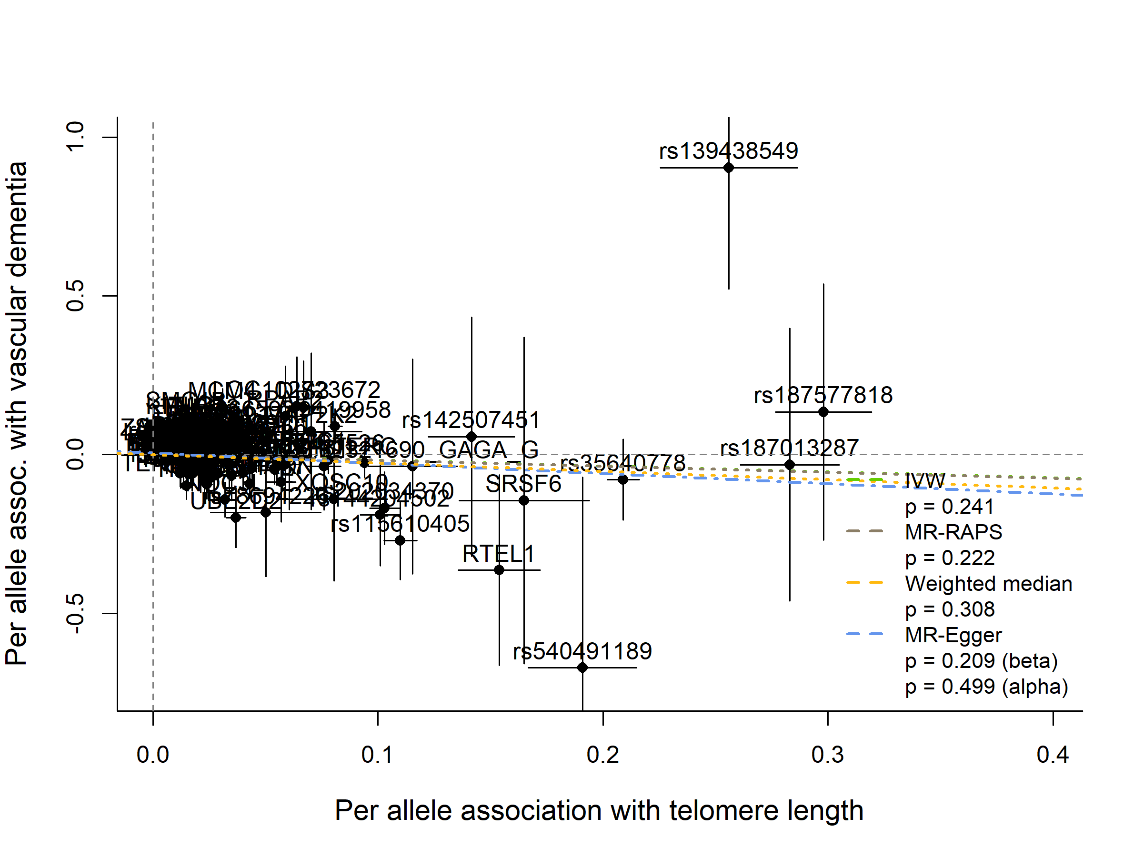


**Figure S14.** SNP-vascular dementia association plotted against SNP-telomere length association, labelled by the mapped gene, with MR slope estimates shown


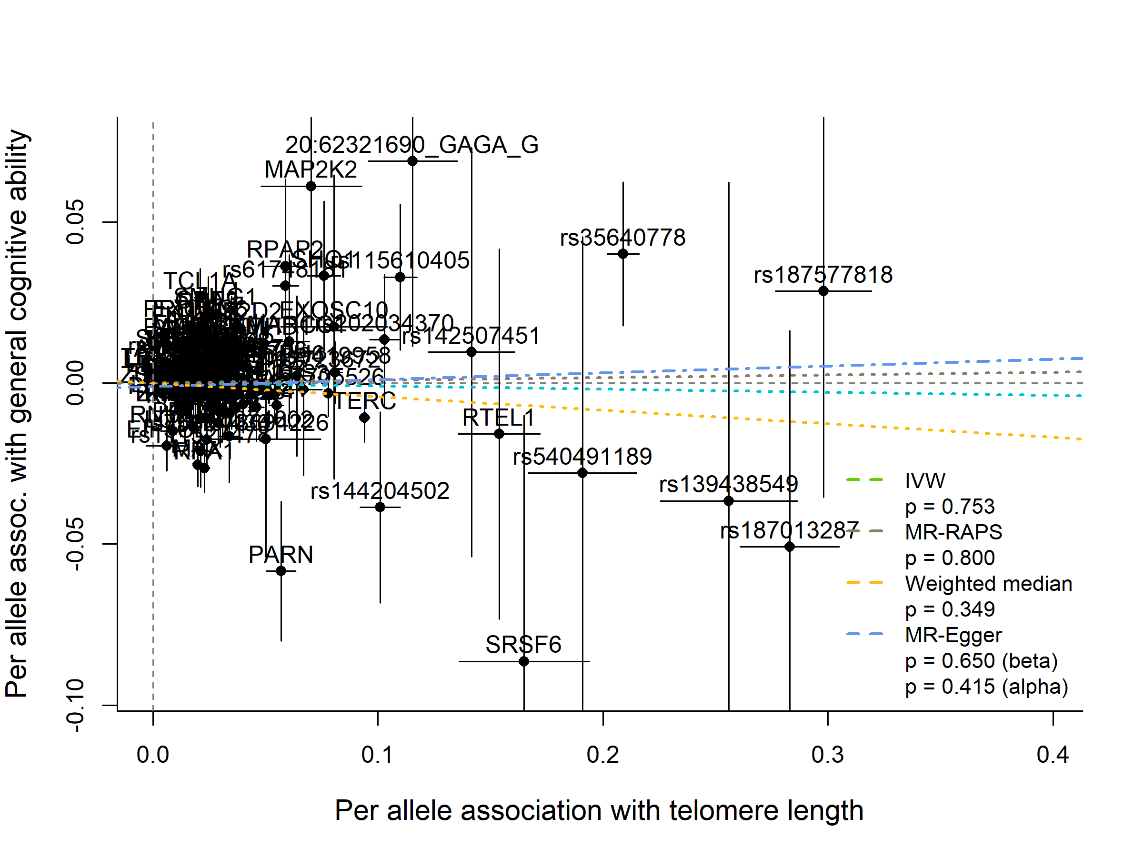


**Figure S15.** SNP-general cognitive ability association plotted against SNP-telomere length association, labelled by the mapped gene, with MR slope estimates shown


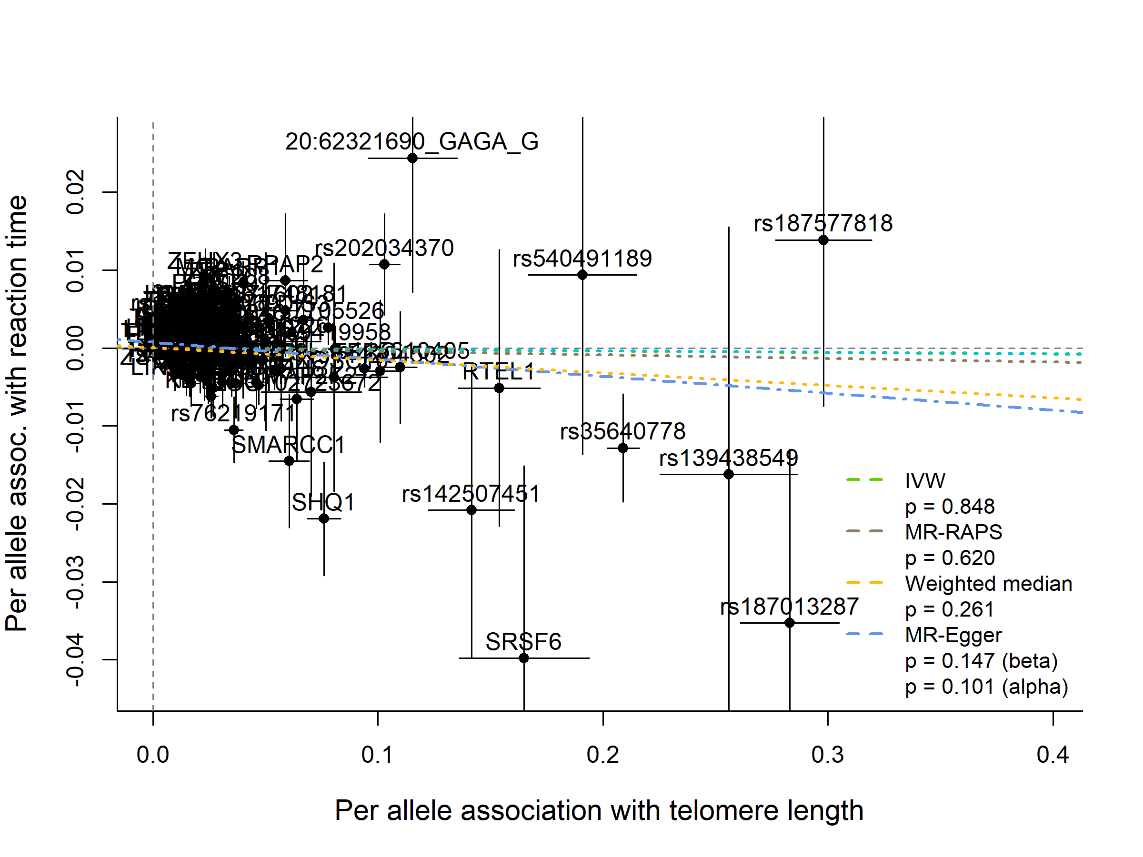


**Figure S16.** SNP-reaction time association plotted against SNP-telomere length association, labelled by the mapped gene, with MR slope estimates shown


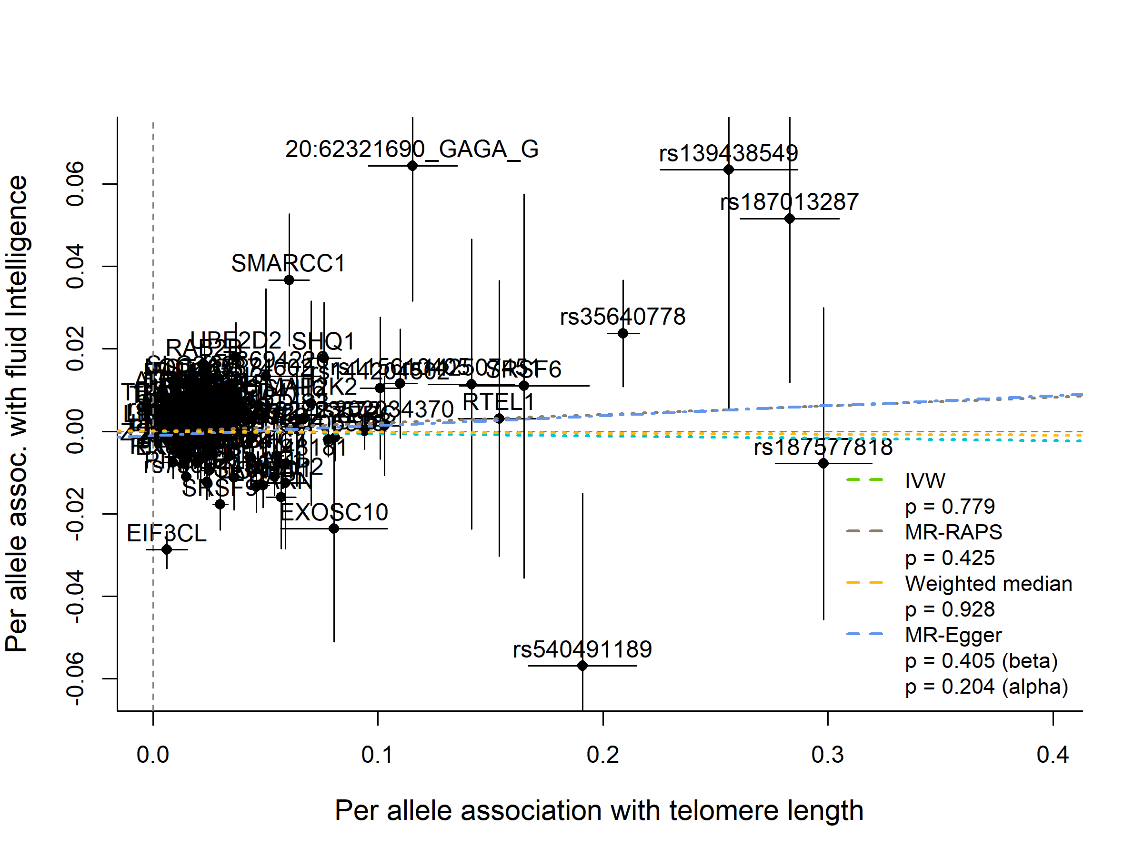


**Figure S17.** SNP-fluid intelligence association plotted against SNP-telomere length association, labelled by the mapped gene, with MR slope estimates shown


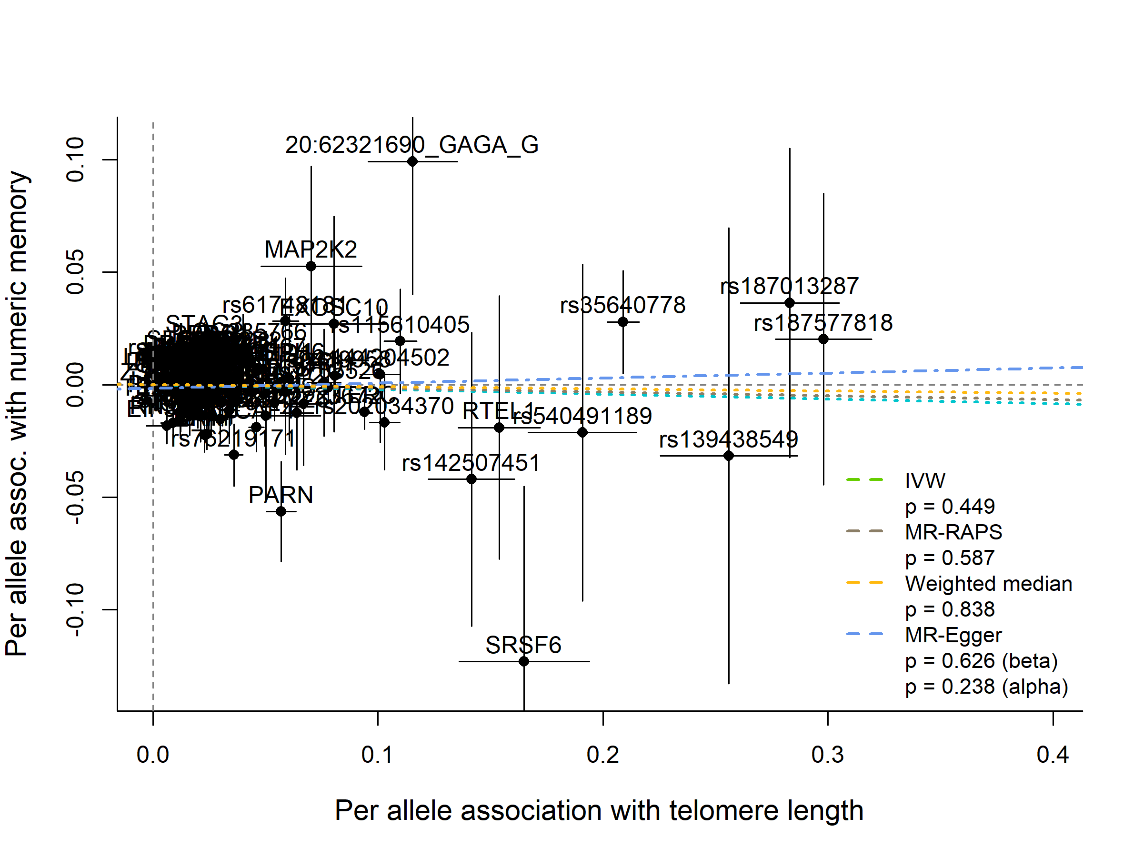


**Figure S18.** SNP-numeric memory association plotted against SNP-telomere length association, labelled by the mapped gene, with MR slope estimates shown


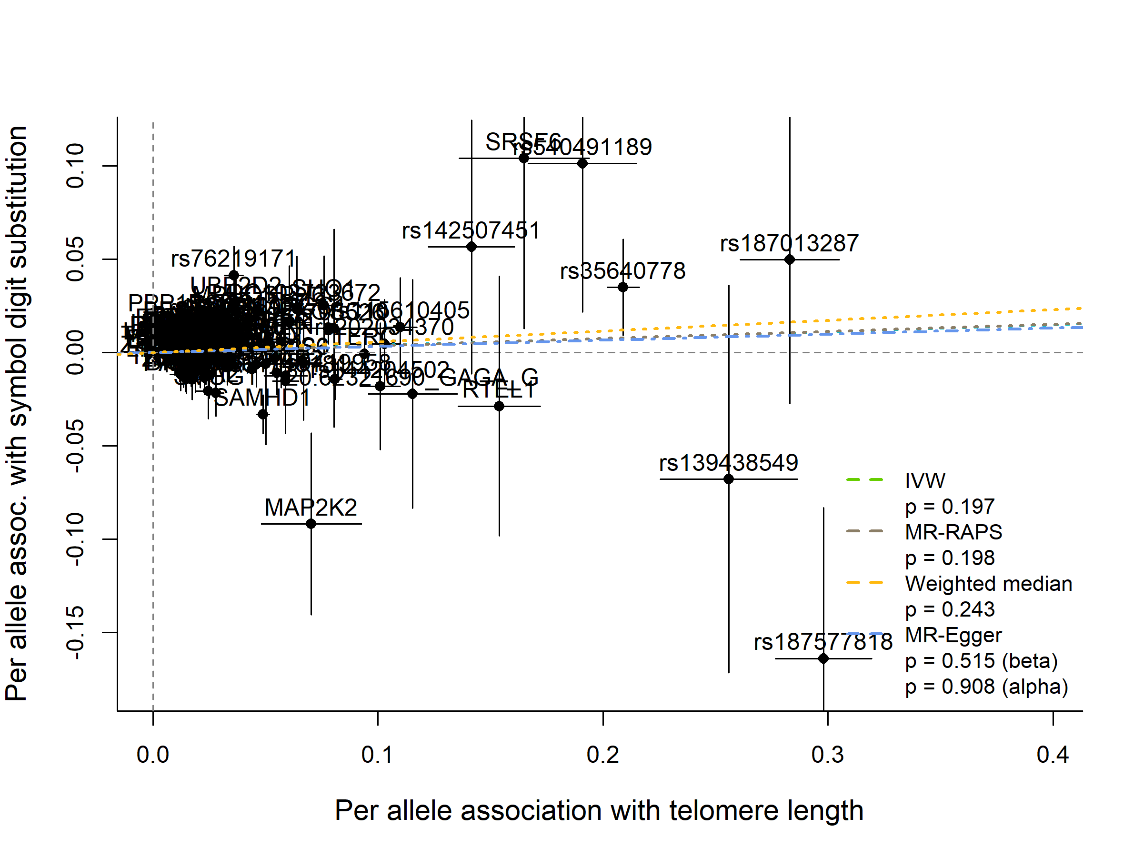


**Figure S19.** SNP-symbol digit substitution association plotted against SNP-telomere length association, labelled by the mapped gene, with MR slope estimates shown


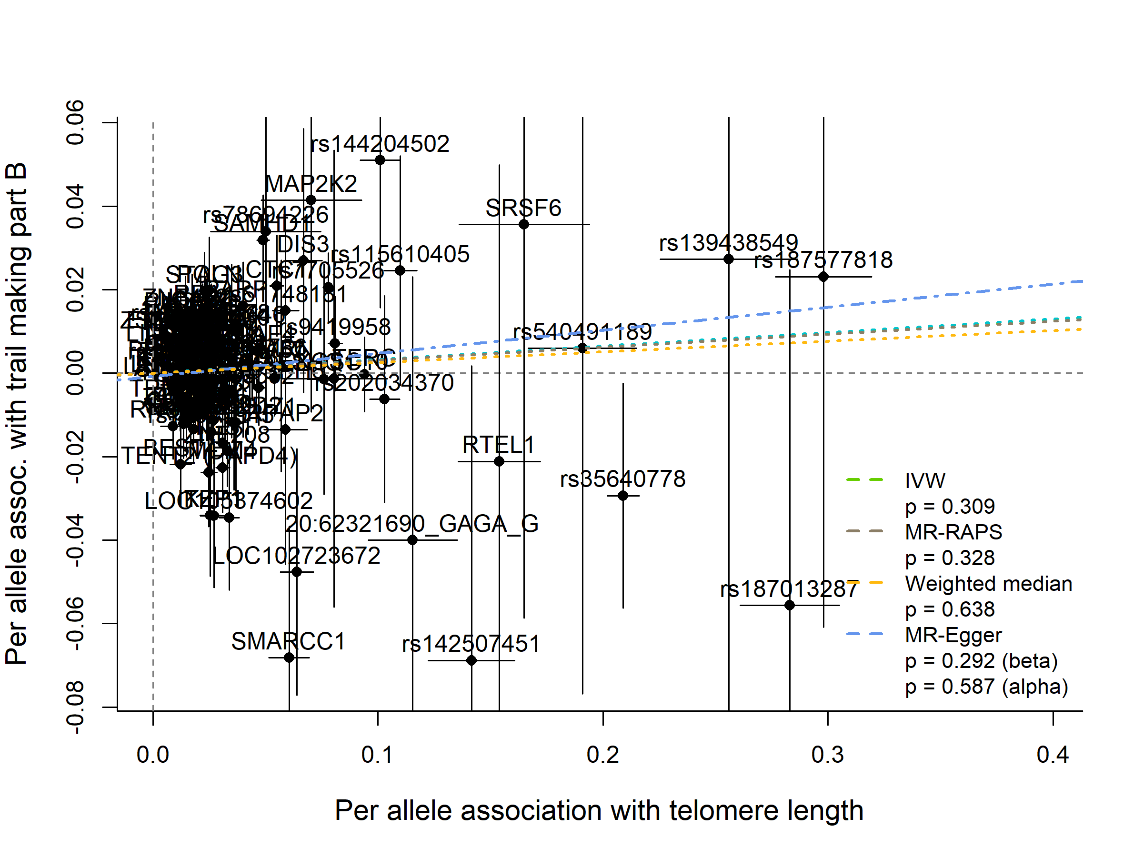


**Figure S20.** SNP-trail making part B association plotted against SNP-telomere length association, labelled by the mapped gene, with MR slope estimates shown


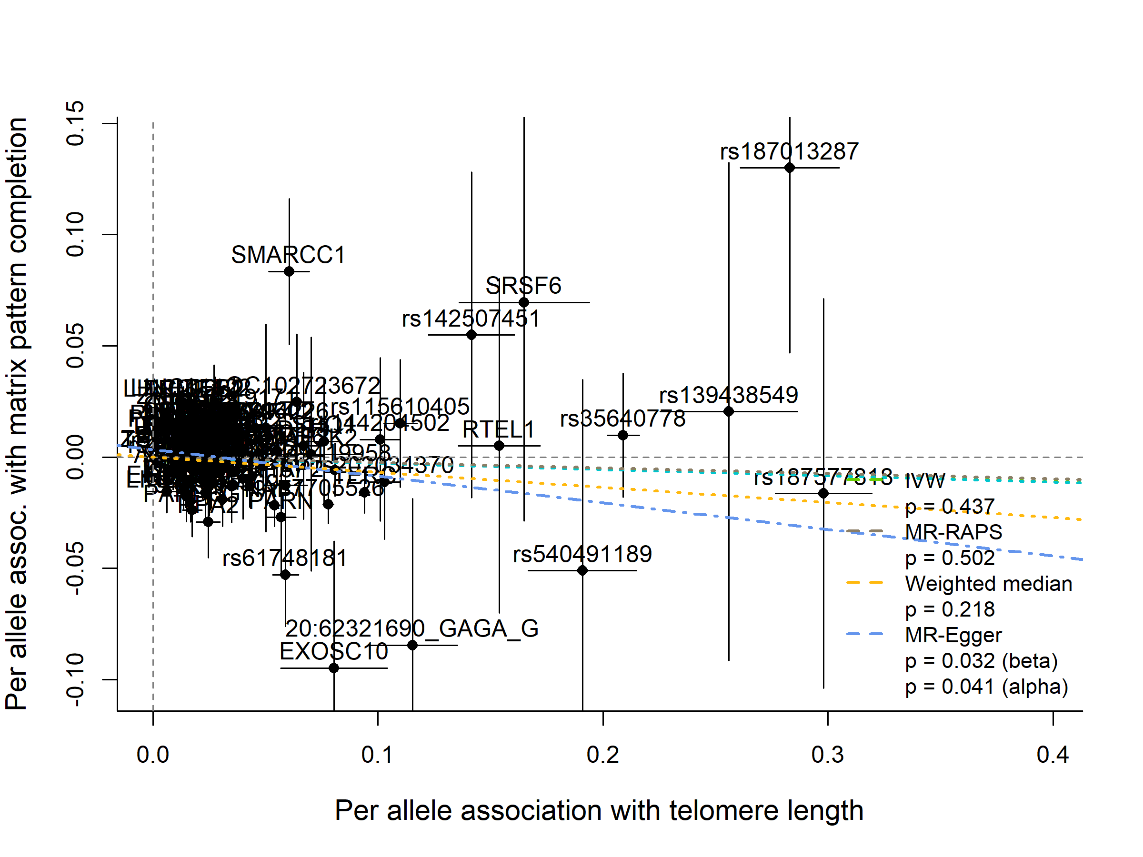


**Figure S21.** SNP-matrix pattern completion association plotted against SNP-telomere length association, labelled by the mapped gene, with MR slope estimates shown


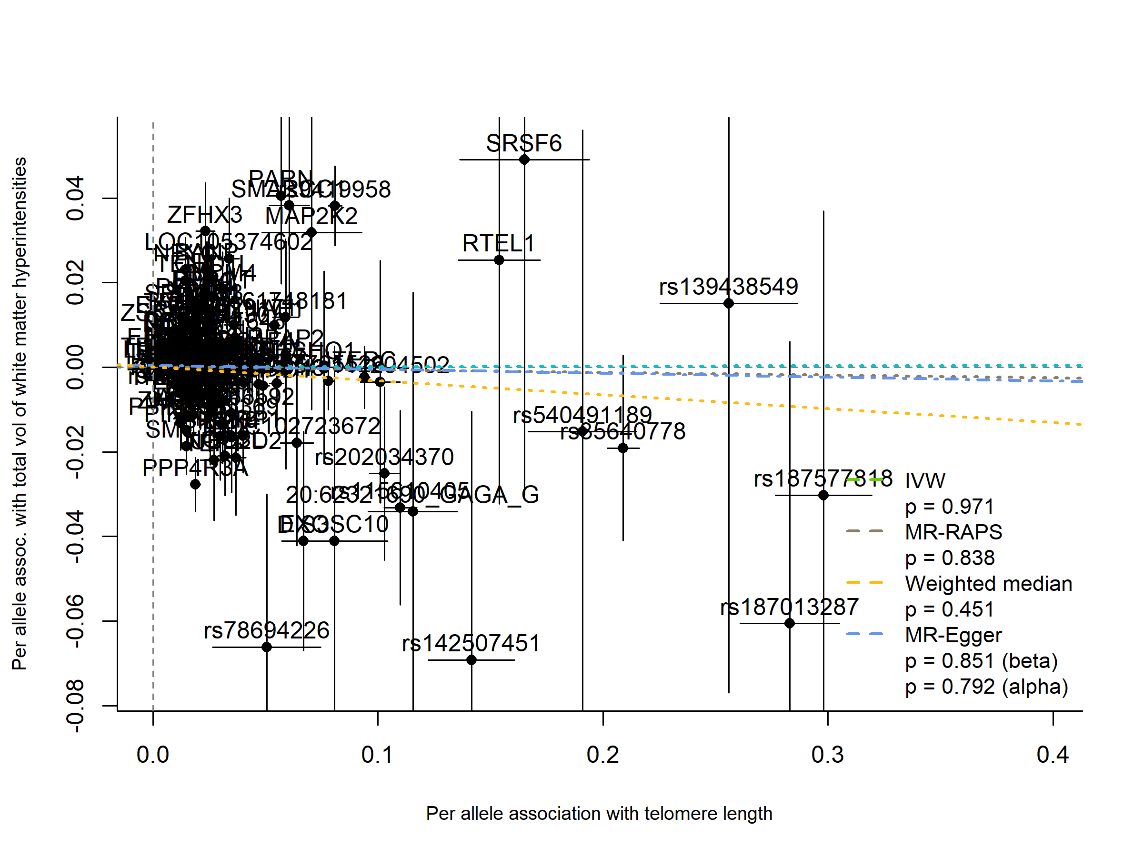


**Figure S22.** SNP-total volume of white matter hyperintensities association plotted against SNP-telomere length association, labelled by the mapped gene, with MR slope estimates shown


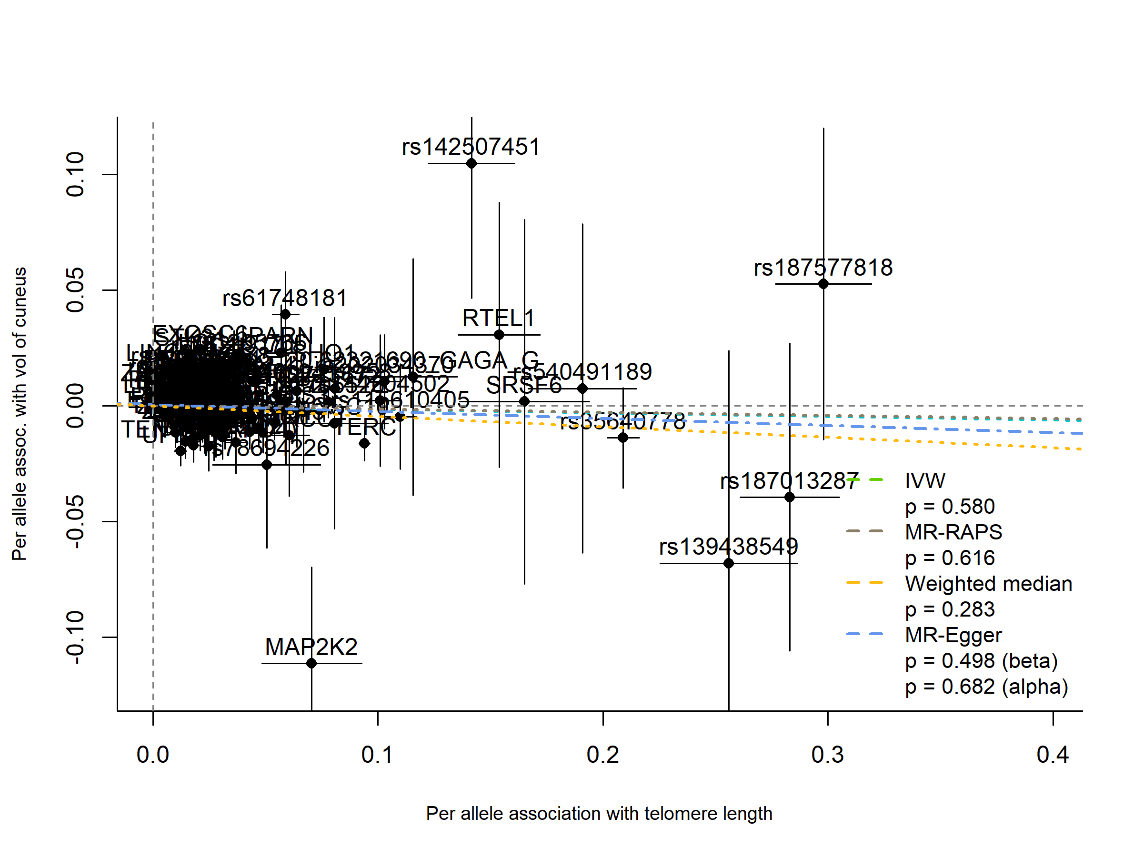


**Figure S23.** SNP-cuneus volume association plotted against SNP-telomere length association, labelled by the mapped gene, with MR slope estimates shown

**
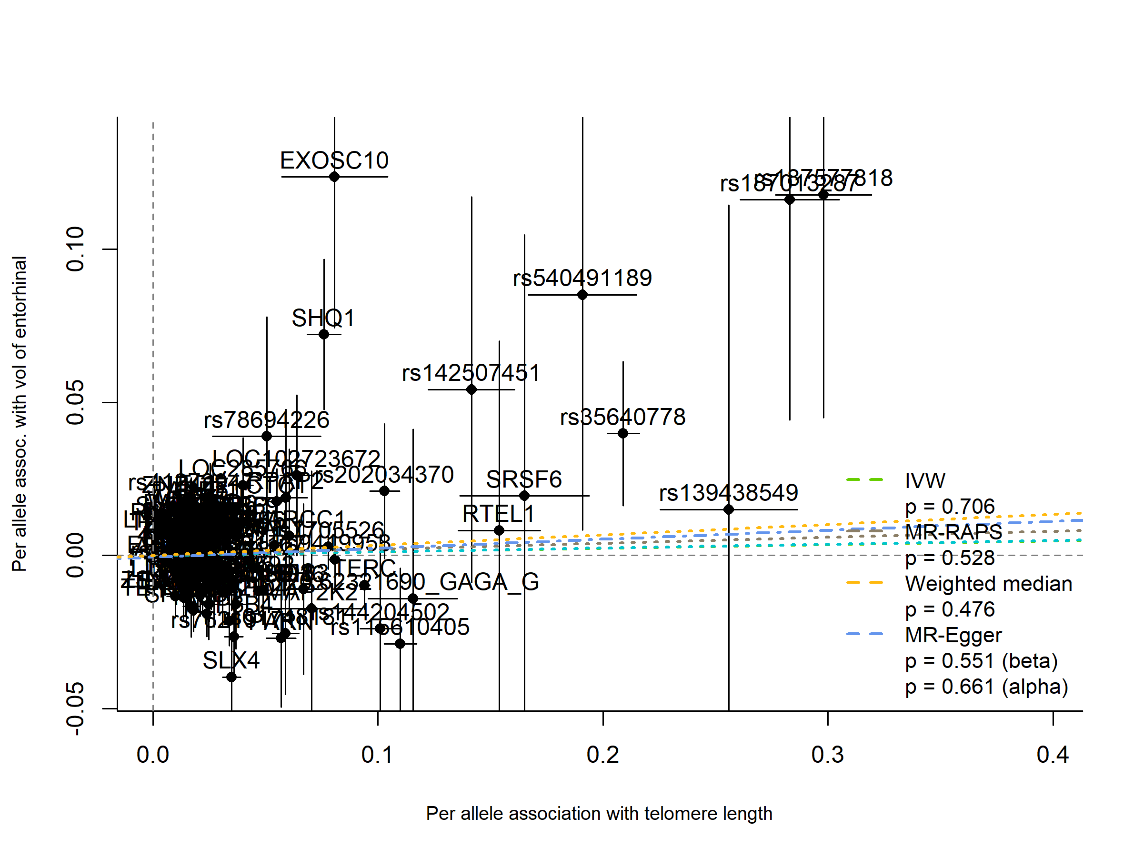
**

**Figure S24.** SNP-entorhinal volume association plotted against SNP-telomere length association, labelled by the mapped gene, with MR slope estimates shown


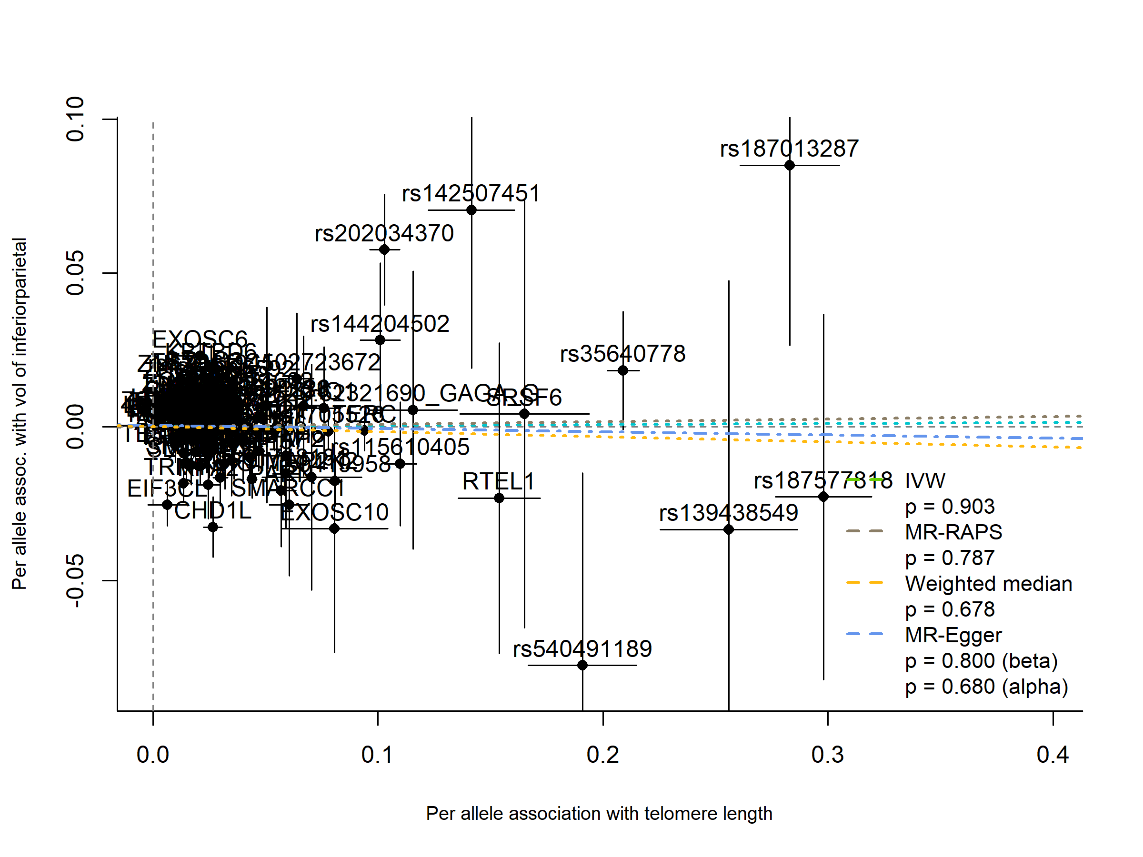


**Figure S25.** SNP-inferiorparietal volume association plotted against SNP-telomere length association, labelled by the mapped gene, with MR slope estimates shown


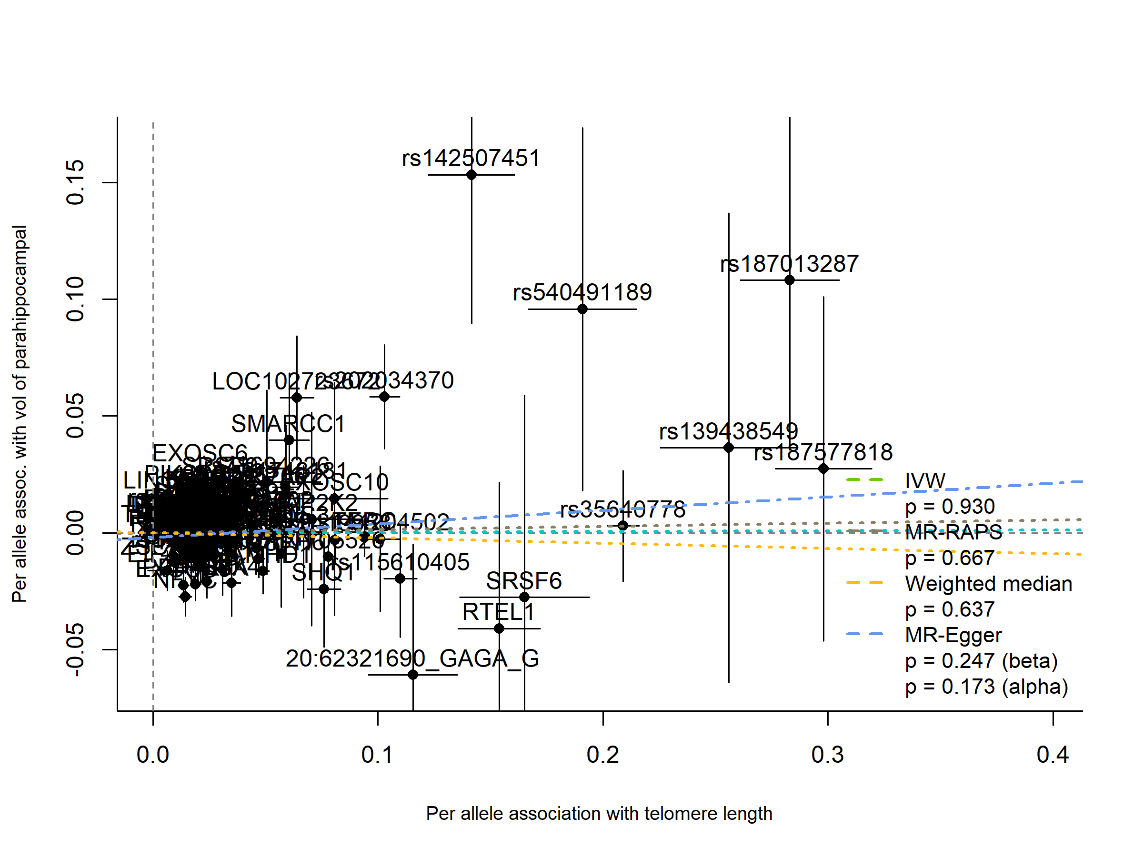


**Figure S26.** SNP-parahippocampal volume association plotted against SNP-telomere length association, labelled by the mapped gene, with MR slope estimates shown


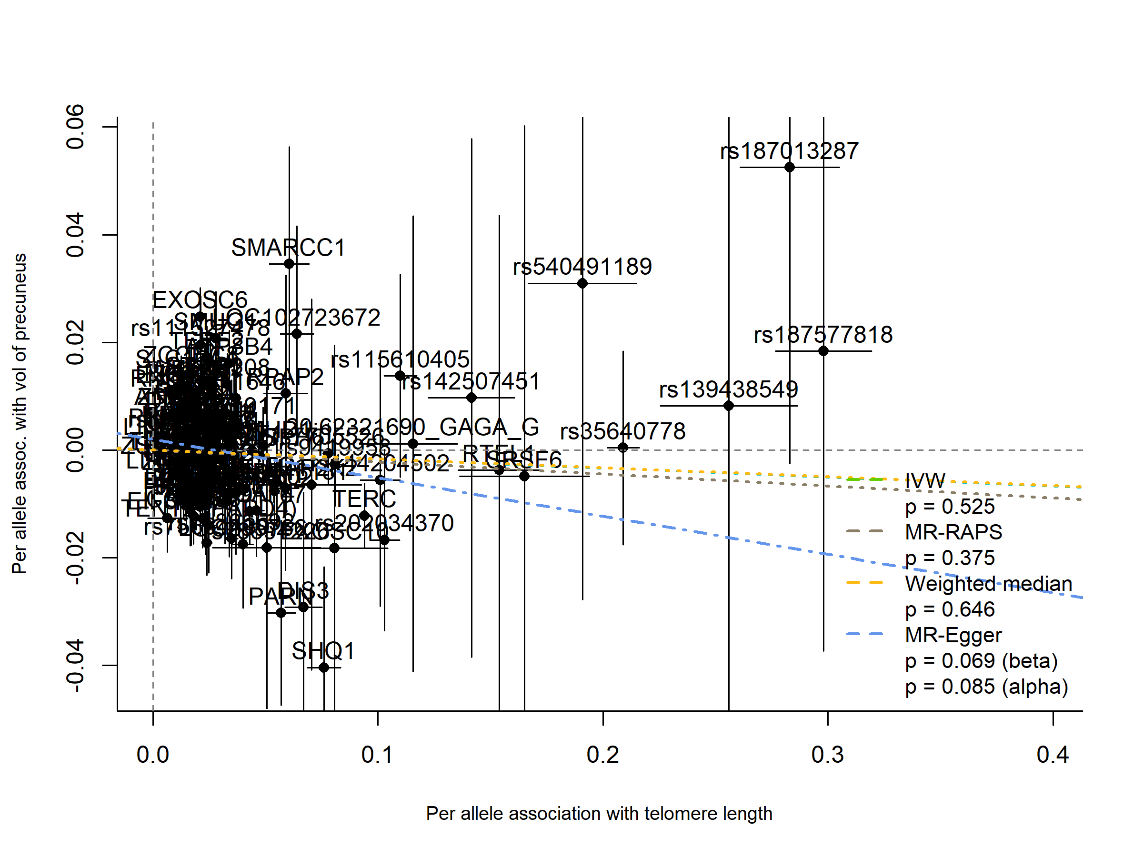


**Figure S27.** SNP-precuneus volume association plotted against SNP-telomere length association, labelled by the mapped gene, with MR slope estimates shown


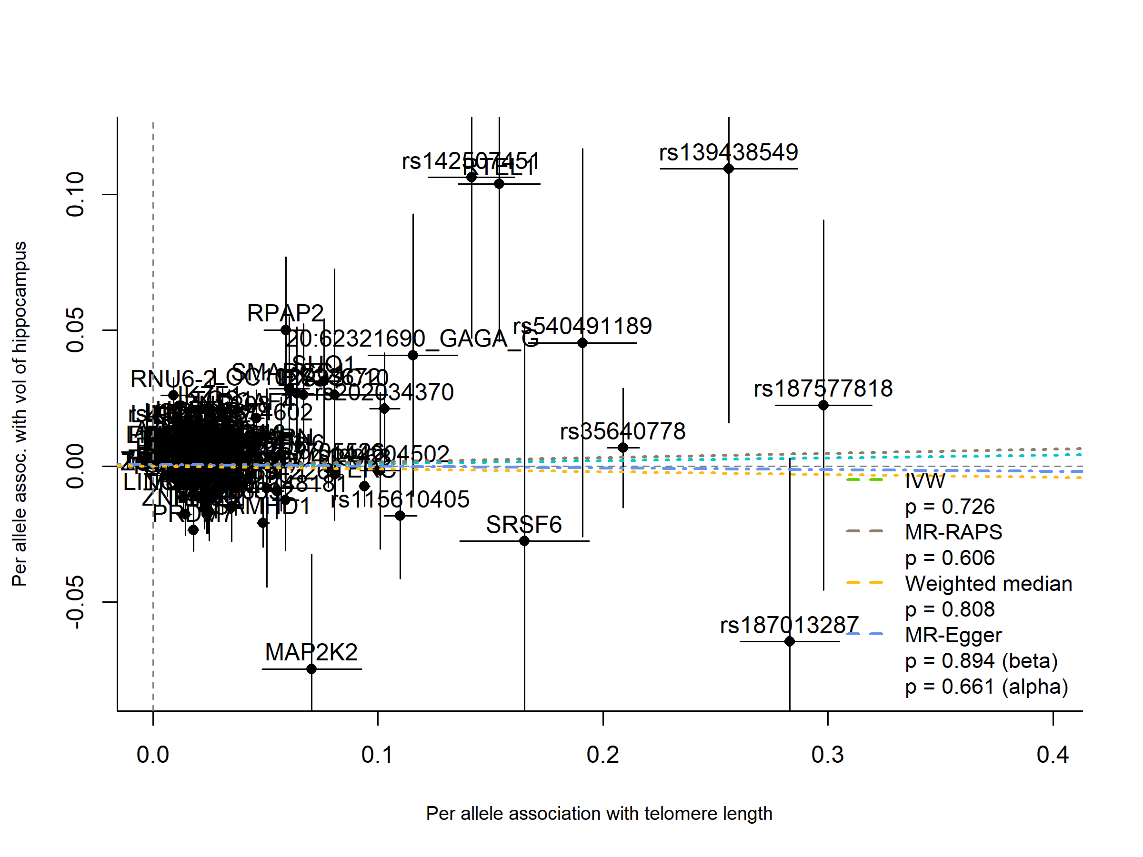


**Figure S28.** SNP-hippocampus volume association plotted against SNP-telomere length association, labelled by the mapped gene, with MR slope estimates shown


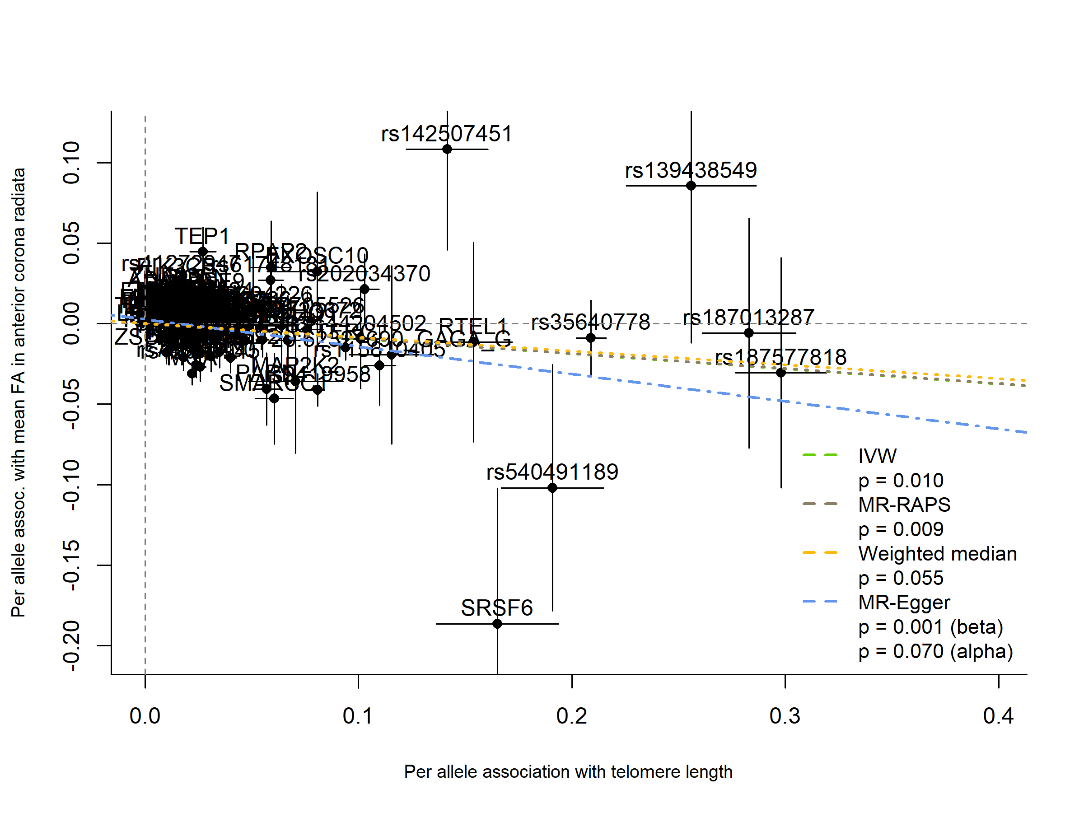


**Figure S29.** SNP-mean FA in anterior corona radiata association plotted against SNP-telomere length association, labelled by the mapped gene, with MR slope estimates shown


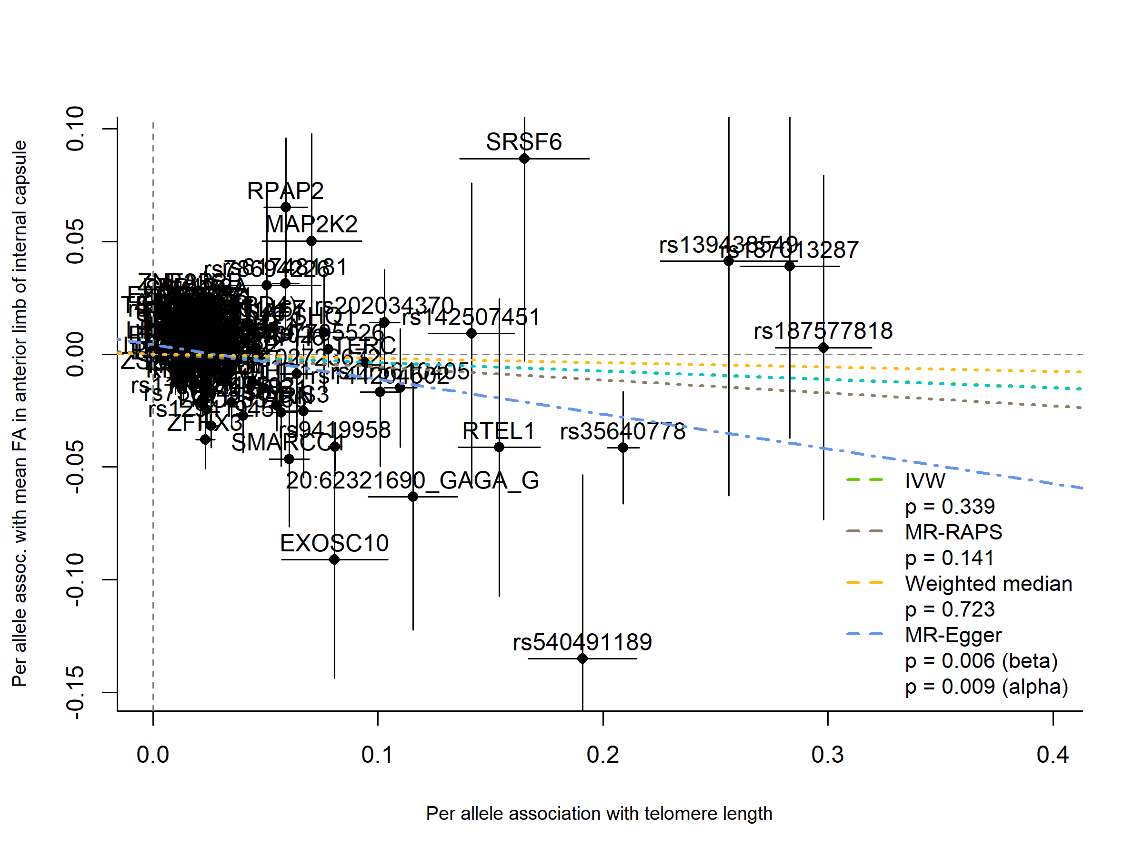


**Figure S30.** SNP-mean FA in anterior limb of internal capsule association plotted against SNP-telomere length association, labelled by the mapped gene, with MR slope estimates shown


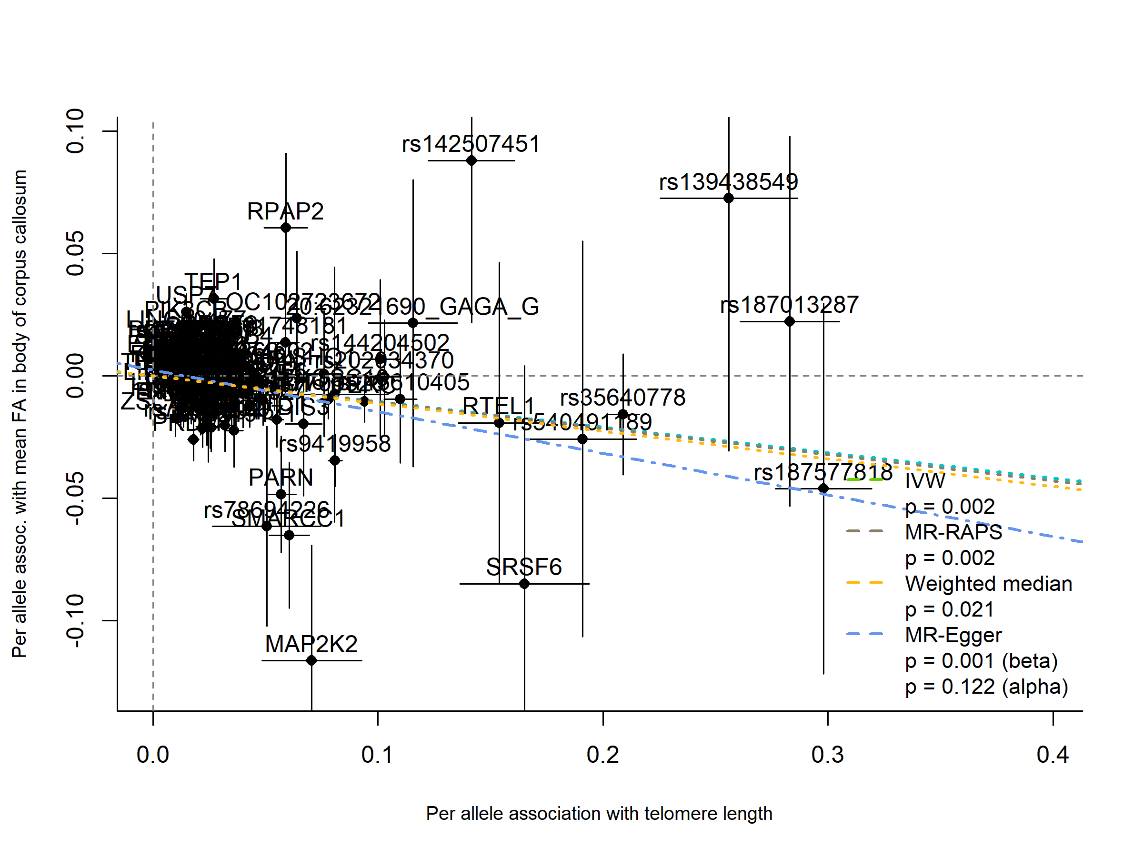


**Figure S31.** SNP-mean FA in body of corpus callosum association plotted against SNP-telomere length association, labelled by the mapped gene, with MR slope estimates shown


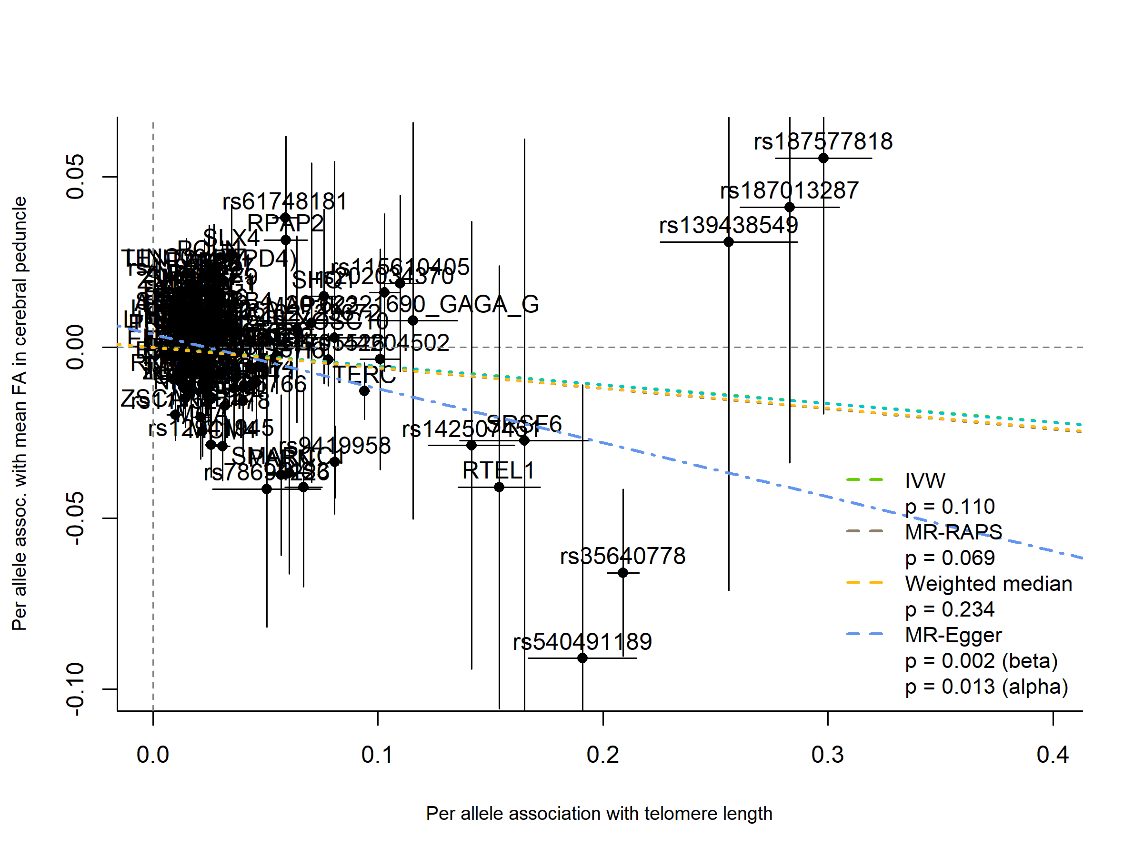


**Figure S32.** SNP-mean FA in cerebral peduncle association plotted against SNP-telomere length association, labelled by the mapped gene, with MR slope estimates shown


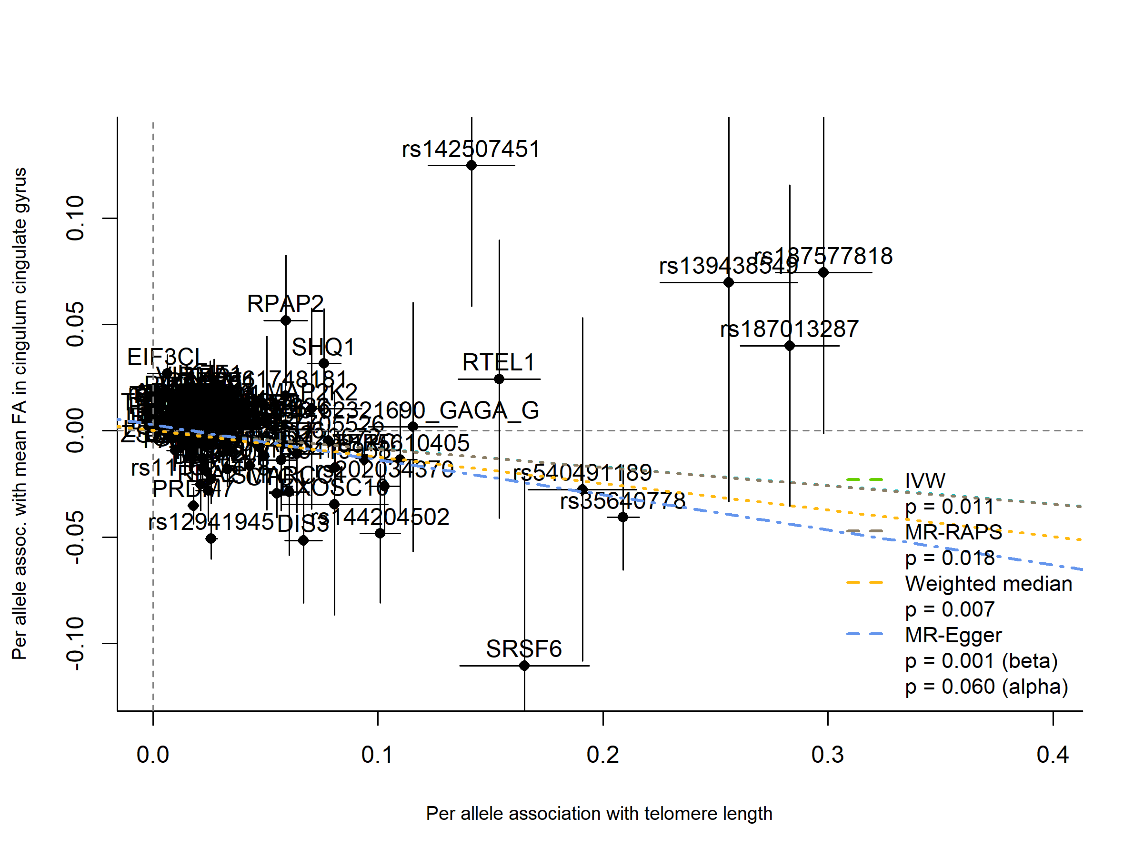


**Figure S33.** SNP-mean FA in cingulum cingulate gyrus association plotted against SNP-telomere length association, labelled by the mapped gene, with MR slope estimates shown


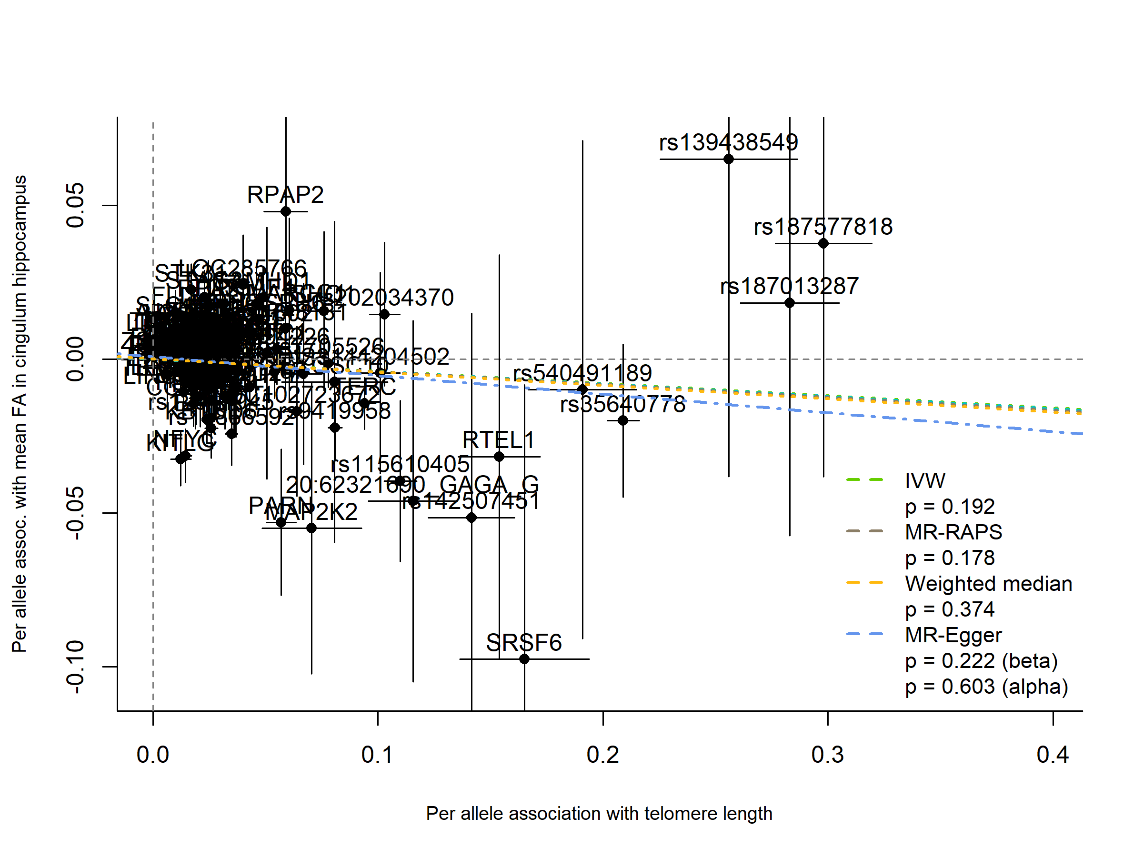


**Figure S34.** SNP-mean FA in cingulum hippocampus association plotted against SNP-telomere length association, labelled by the mapped gene, with MR slope estimates shown


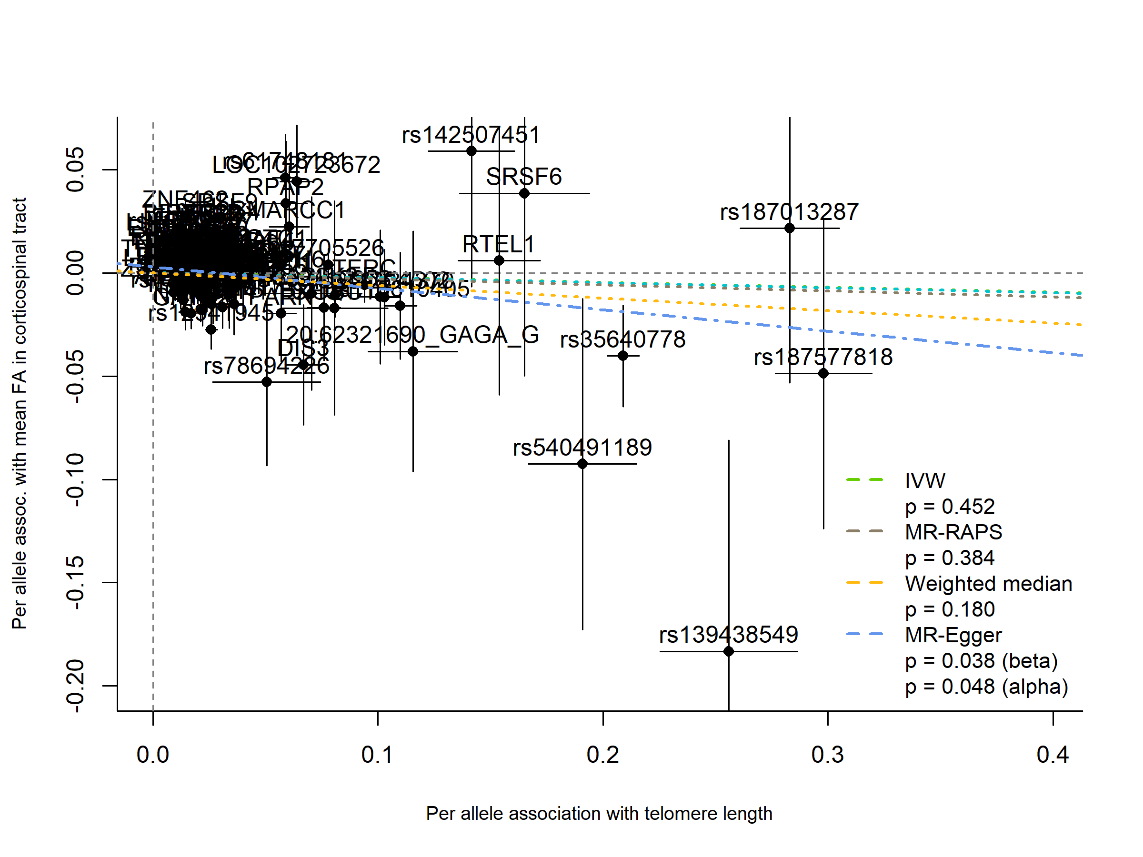


**Figure S35.** SNP-mean FA in corticospinal tract association plotted against SNP-telomere length association, labelled by the mapped gene, with MR slope estimates shown


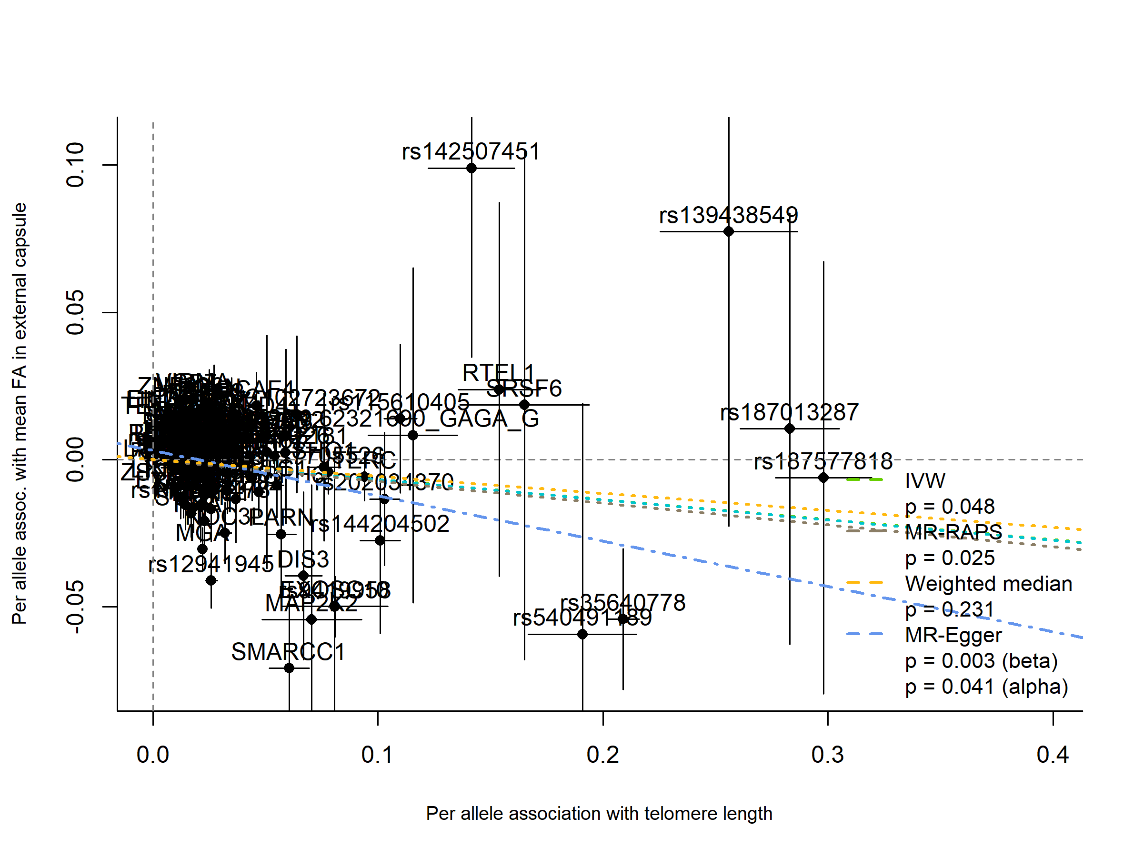


**Figure S36.** SNP-mean FA in external capsule association plotted against SNP-telomere length association, labelled by the mapped gene, with MR slope estimates shown


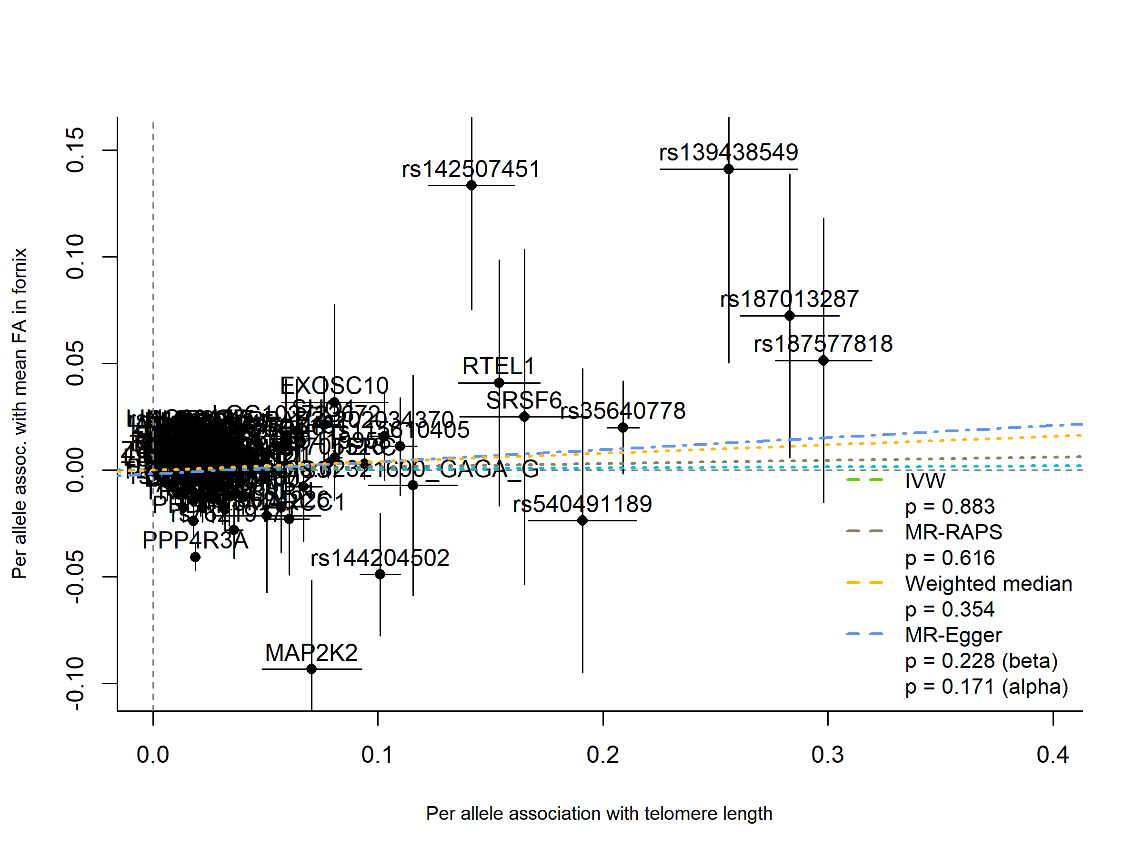


**Figure S37.** SNP-mean FA in fornix association plotted against SNP-telomere length association, labelled by the mapped gene, with MR slope estimates shown


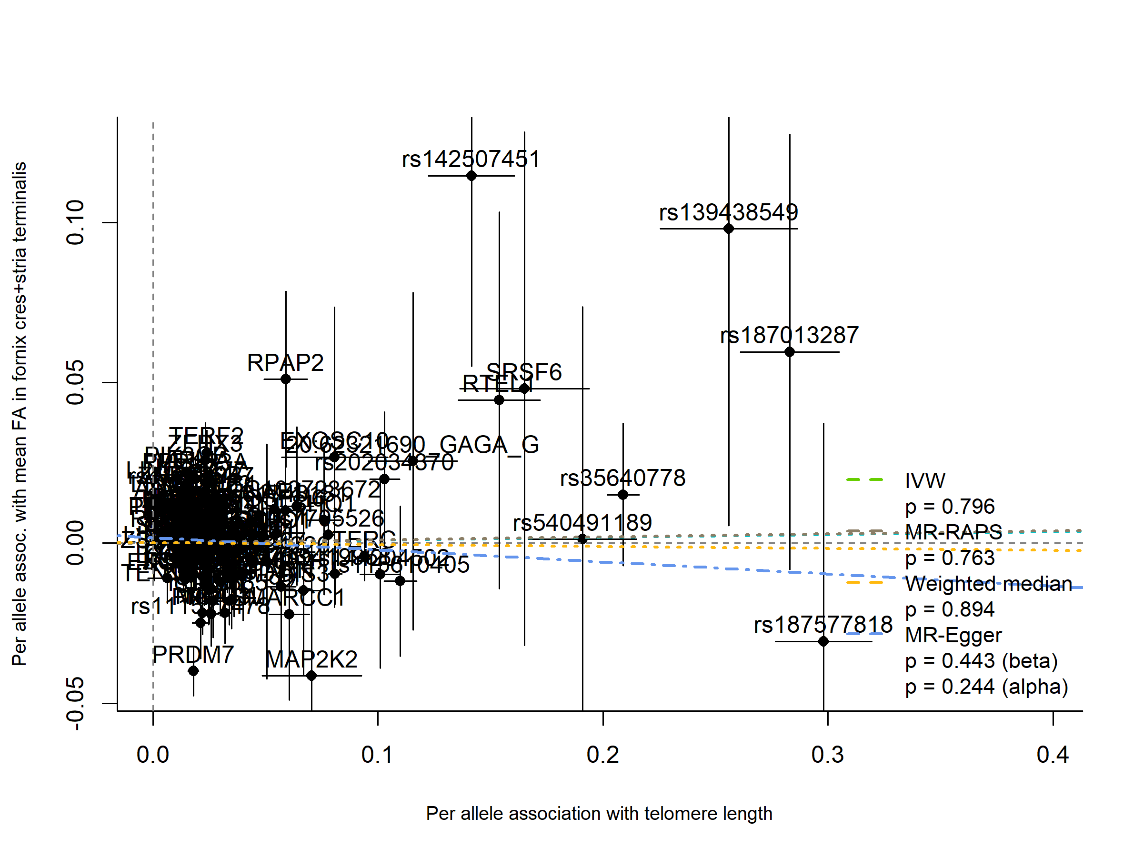


**Figure S38.** SNP-mean FA in fornix cres+stria terminalis association plotted against SNP-telomere length association, labelled by the mapped gene, with MR slope estimates shown


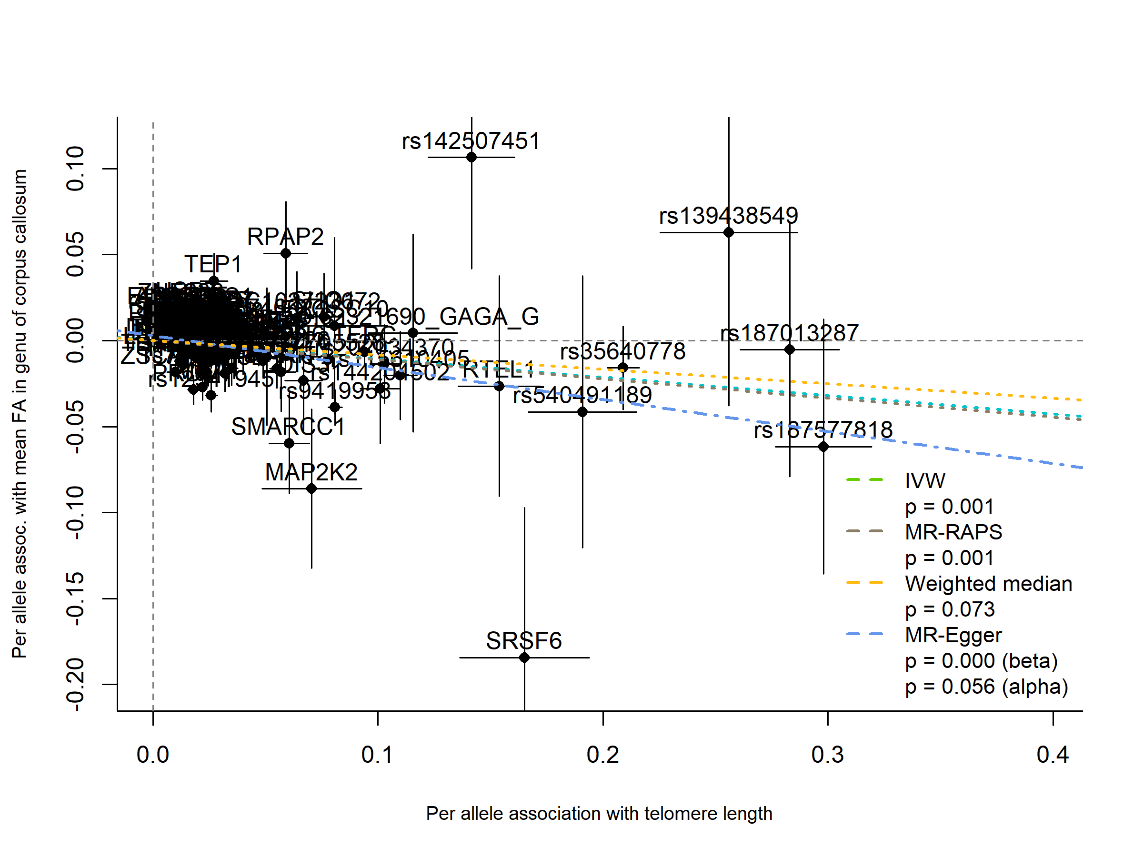


**Figure S39.** SNP-mean FA in genu of corpus callosum association plotted against SNP-telomere length association, labelled by the mapped gene, with MR slope estimates shown


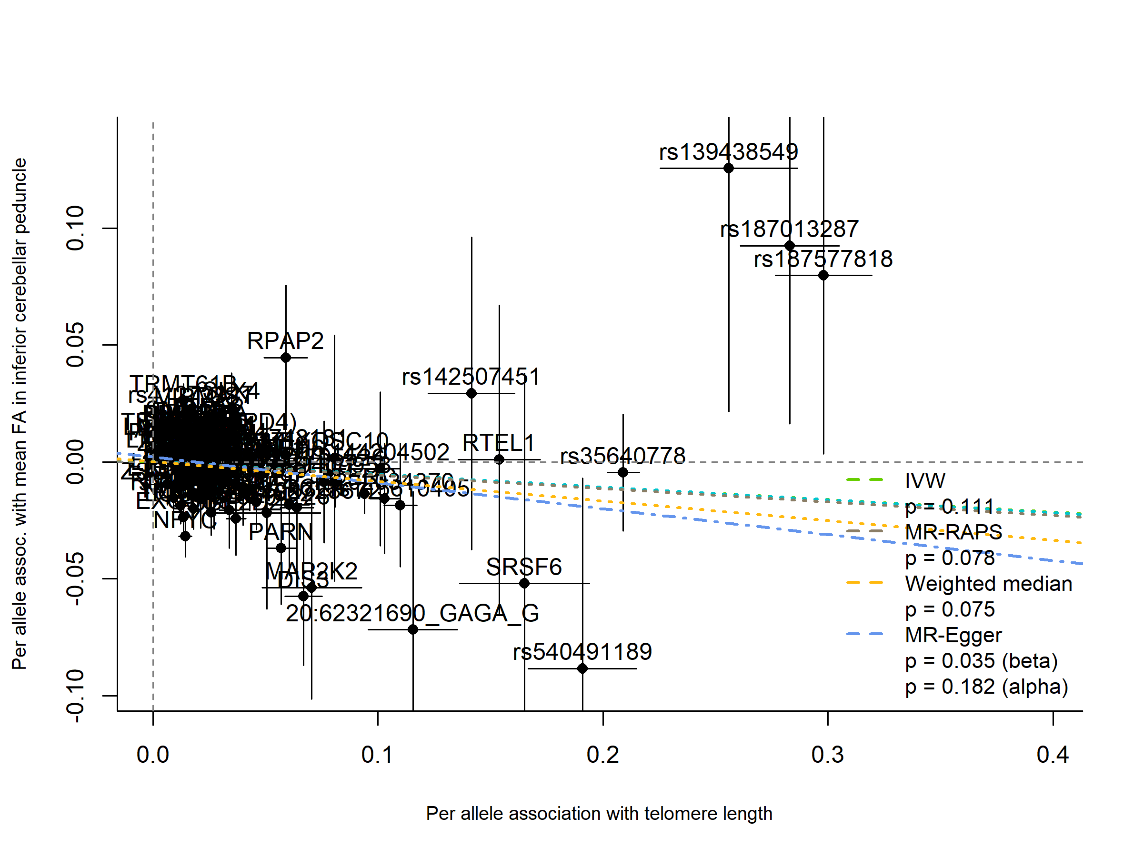


**Figure S40.** SNP-mean FA in inferior cerebellar peduncle association plotted against SNP-telomere length association, labelled by the mapped gene, with MR slope estimates shown


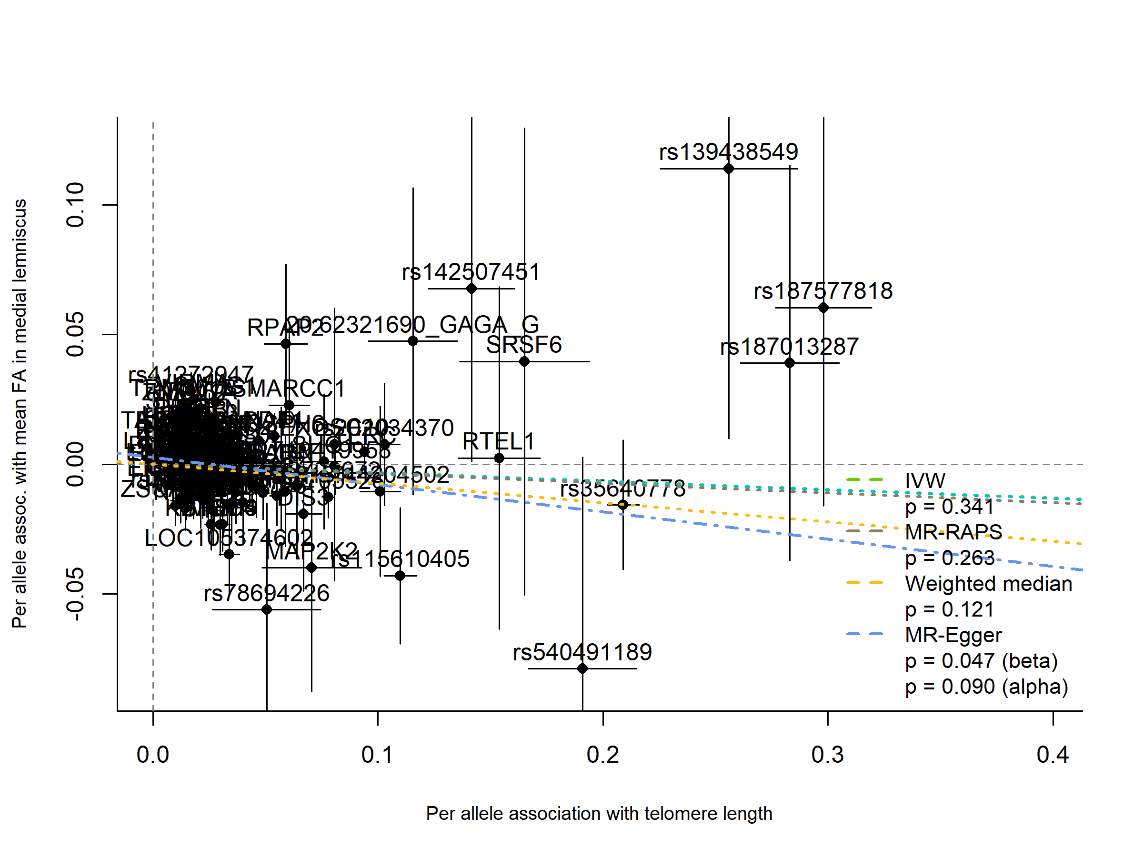


**Figure S41.** SNP-mean FA in medial lemniscus association plotted against SNP-telomere length association, labelled by the mapped gene, with MR slope estimates shown


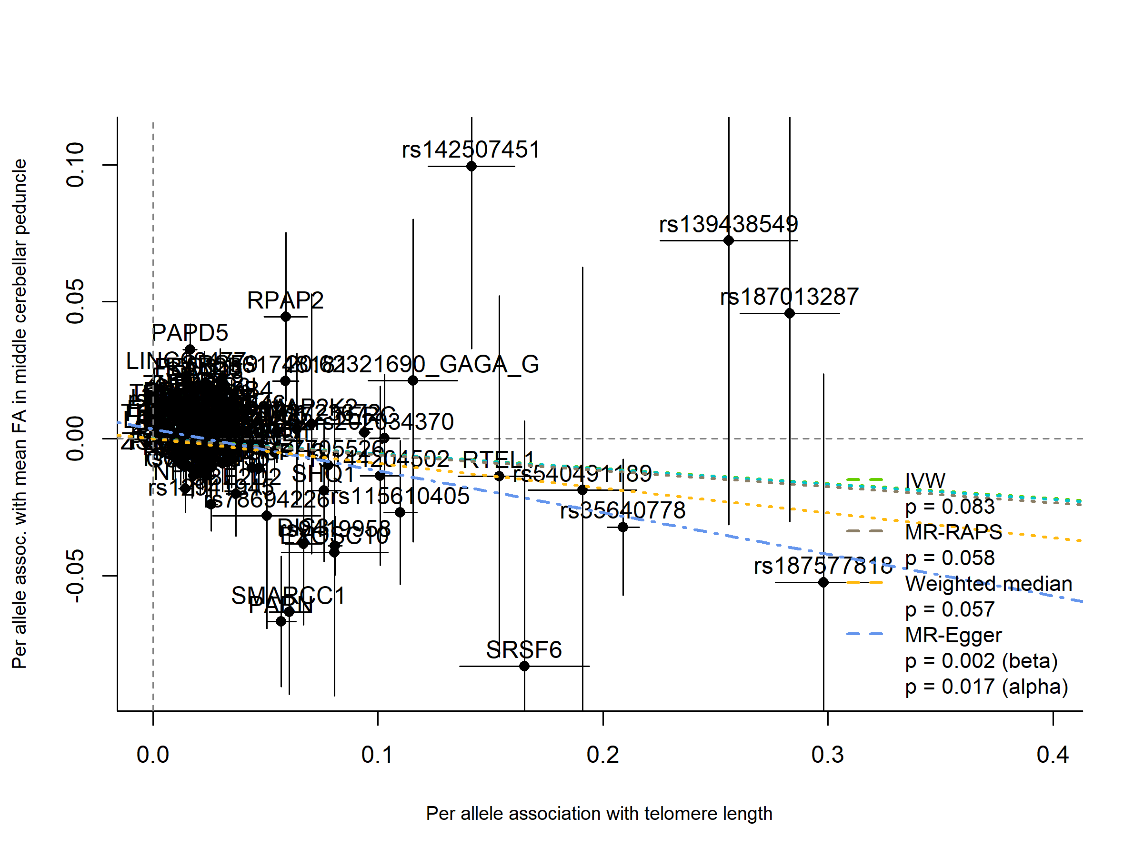


**Figure S42.** SNP-mean FA in middle cerebellar peduncle association plotted against SNP-telomere length association, labelled by the mapped gene, with MR slope estimates shown


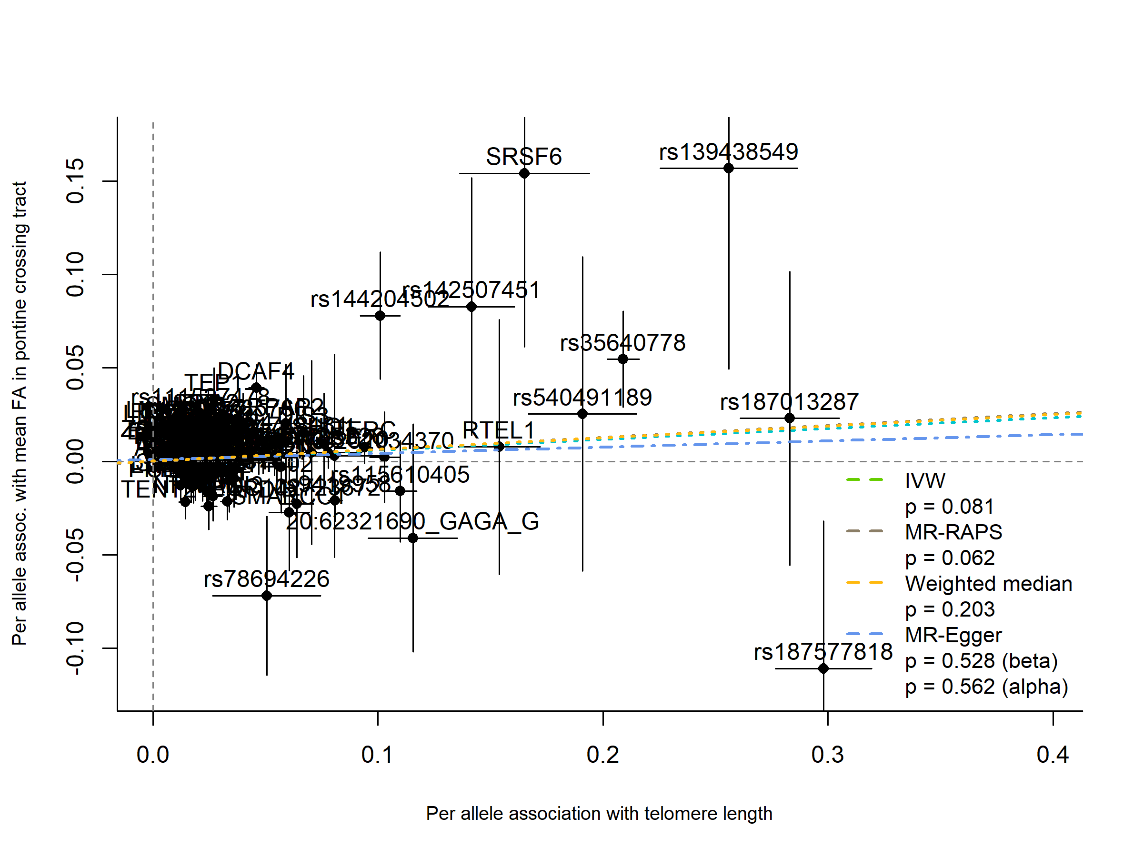


**Figure S43.** SNP-mean FA in pontine crossing tract association plotted against SNP-telomere length association, labelled by the mapped gene, with MR slope estimates shown


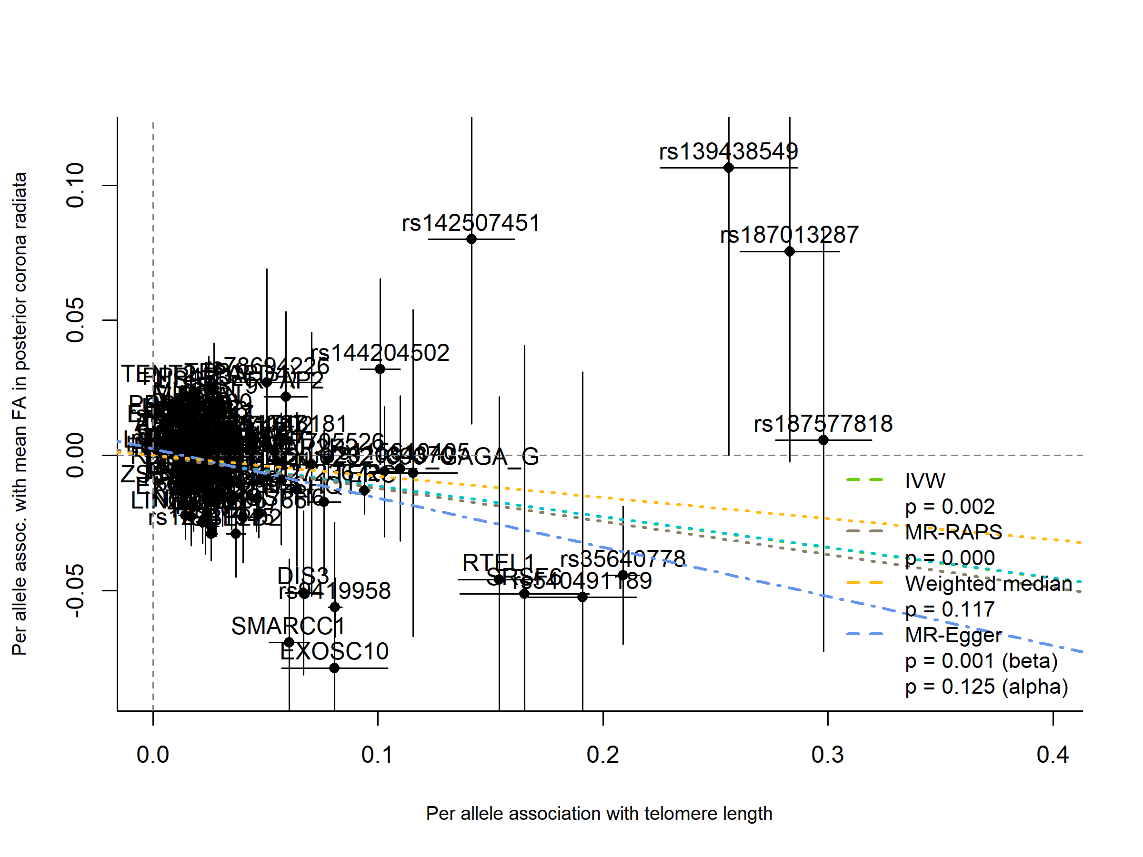


**Figure S44.** SNP-mean FA in posterior corona radiata association plotted against SNP-telomere length association, labelled by the mapped gene, with MR slope estimates shown


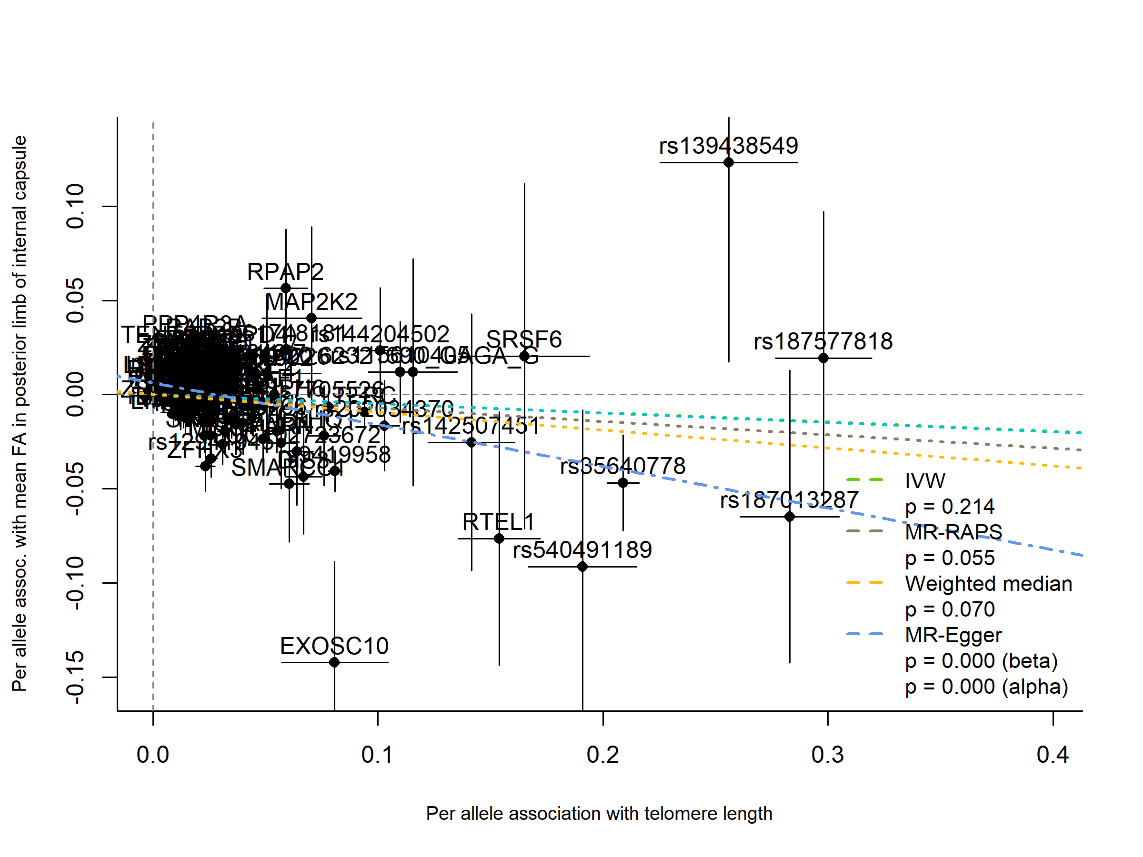


**Figure S45.** SNP-mean FA in posterior limb of internal capsule association plotted against SNP-telomere length association, labelled by the mapped gene, with MR slope estimates shown


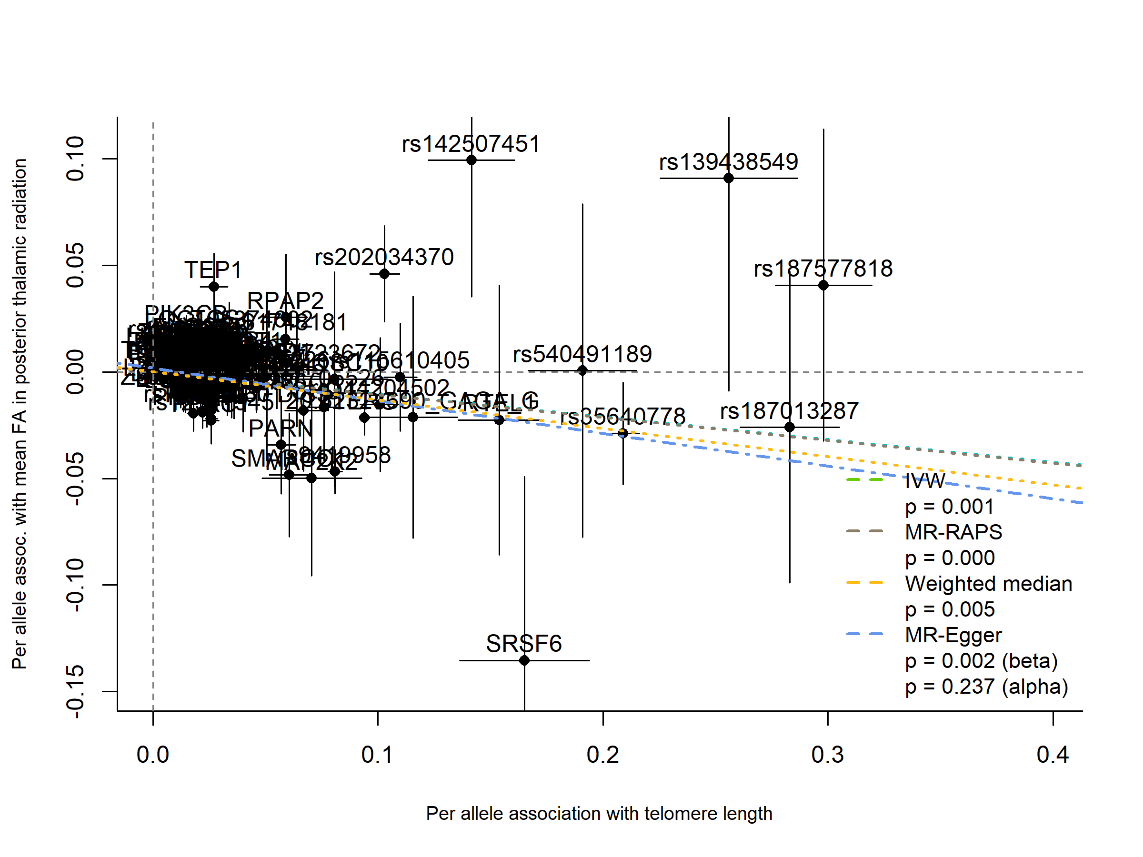


**Figure S46.** SNP-mean FA in posterior thalamic radiation association plotted against SNP-telomere length association, labelled by the mapped gene, with MR slope estimates shown


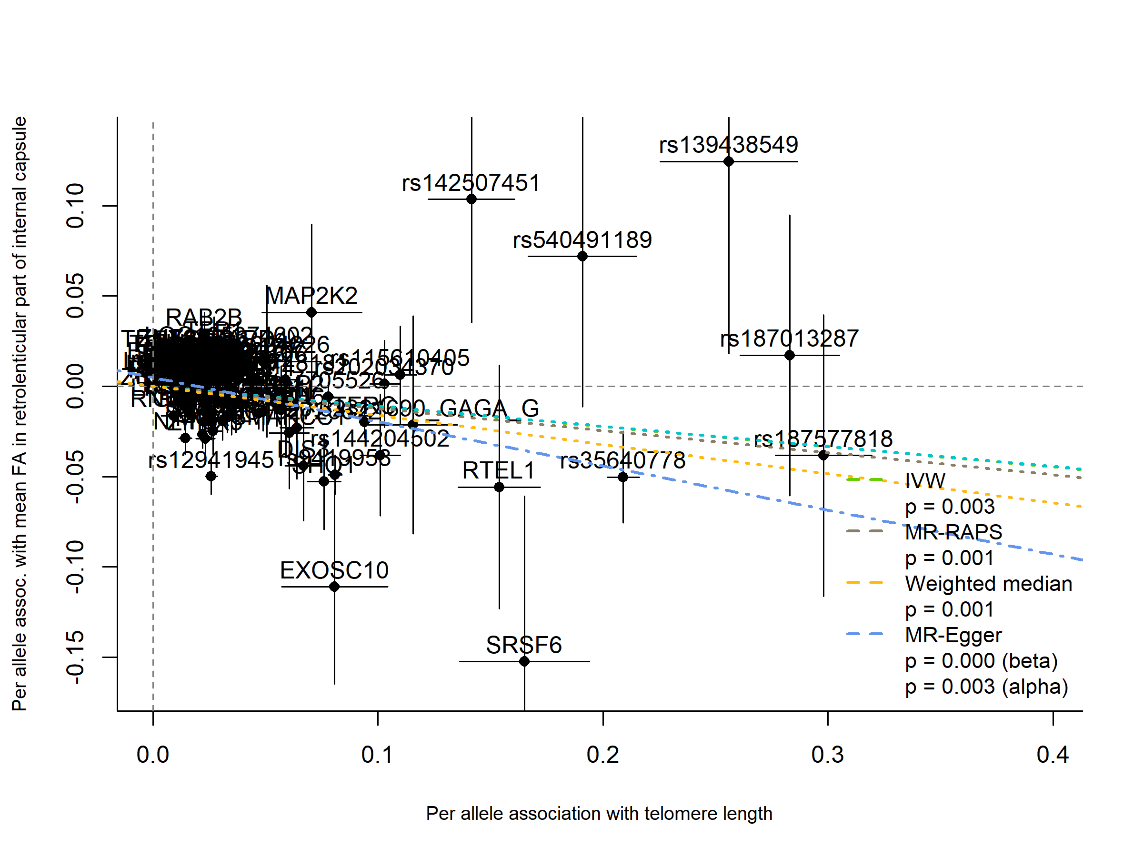


**Figure S47.** SNP-mean FA in retrolenticular part of internal capsule association plotted against SNP-telomere length association, labelled by the mapped gene, with MR slope estimates shown


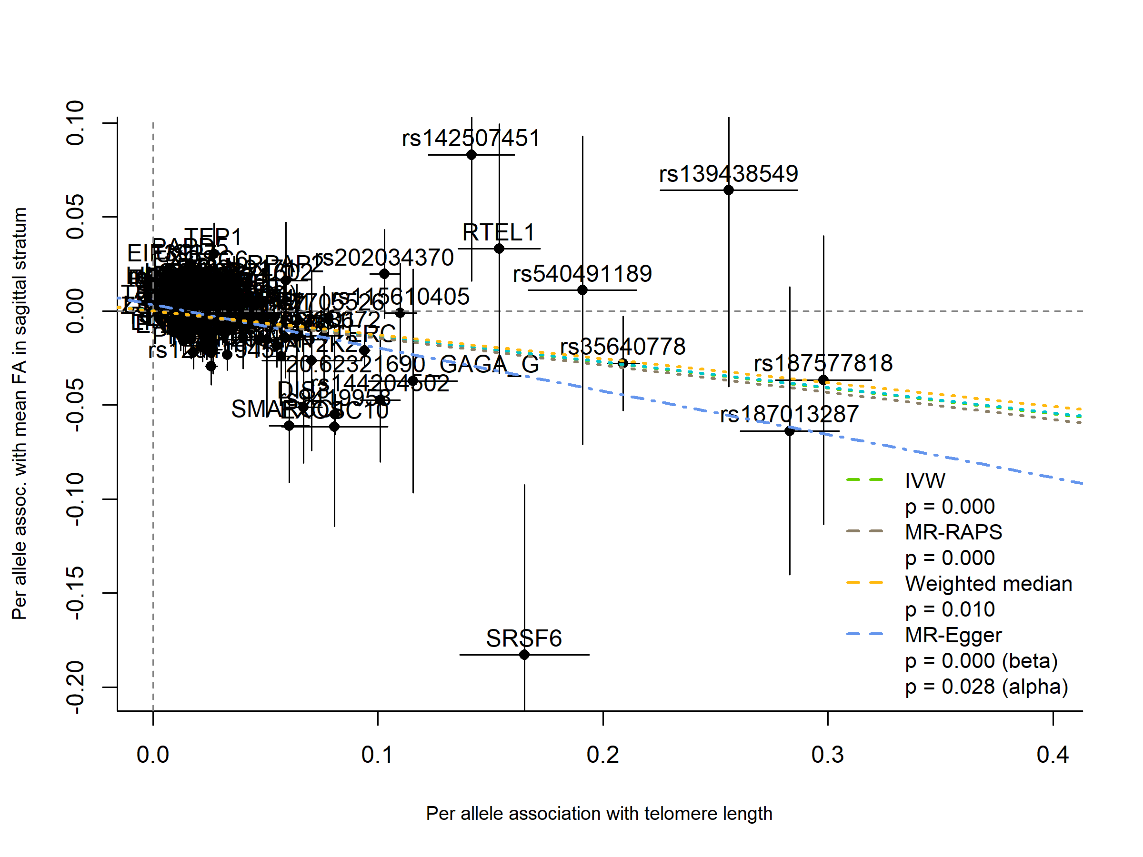


**Figure S48.** SNP-mean FA in sagittal stratum association plotted against SNP-telomere length association, labelled by the mapped gene, with MR slope estimates shown


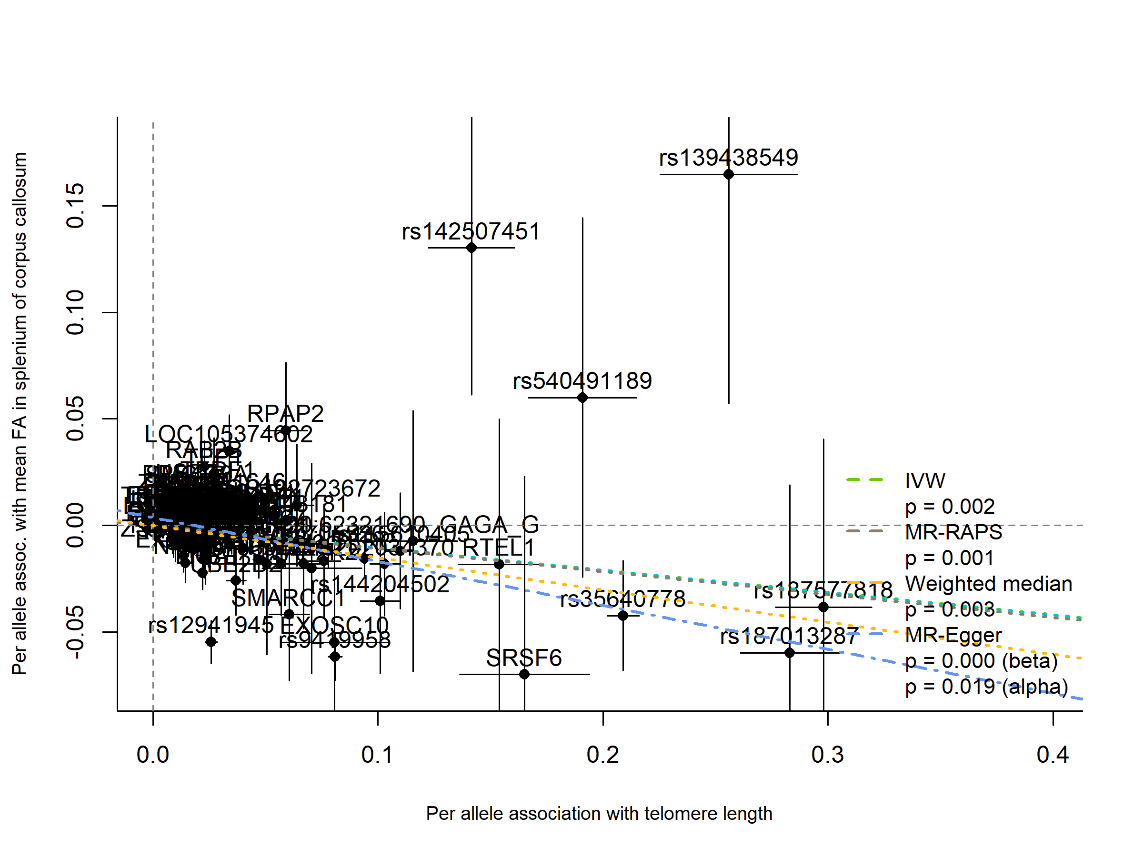


**Figure S49.** SNP-mean FA in splenium of corpus callosum association plotted against SNP-telomere length association, labelled by the mapped gene, with MR slope estimates shown


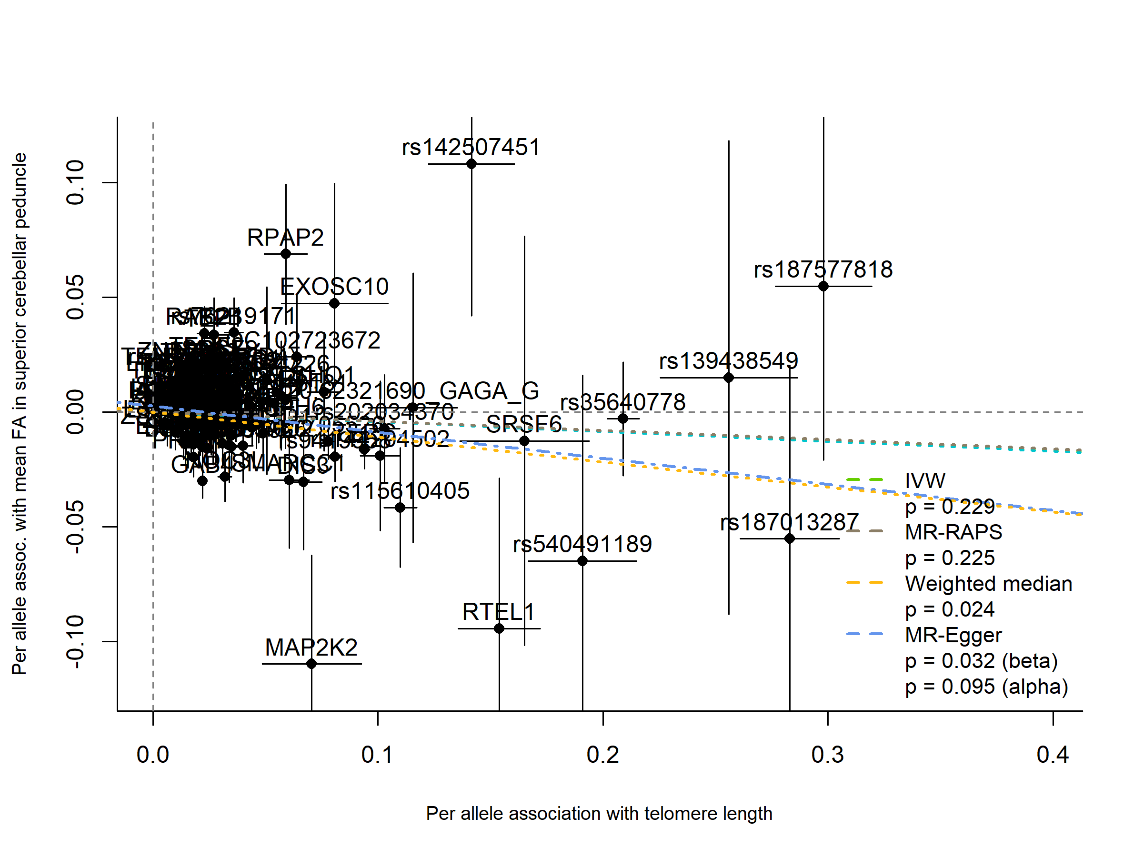


**Figure S50.** SNP-mean FA in superior cerebellar peduncle association plotted against SNP-telomere length association, labelled by the mapped gene, with MR slope estimates shown


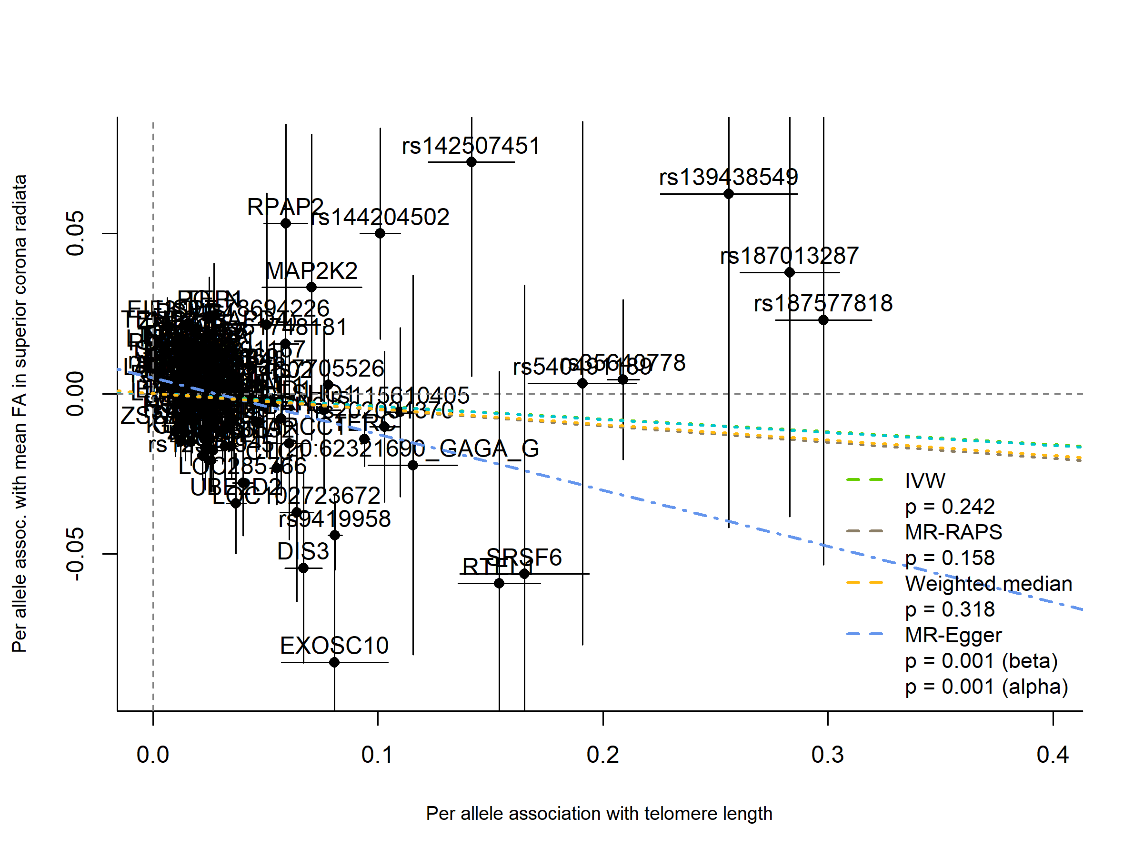


**Figure S51.** SNP-mean FA in superior corona radiata association plotted against SNP-telomere length association, labelled by the mapped gene, with MR slope estimates shown


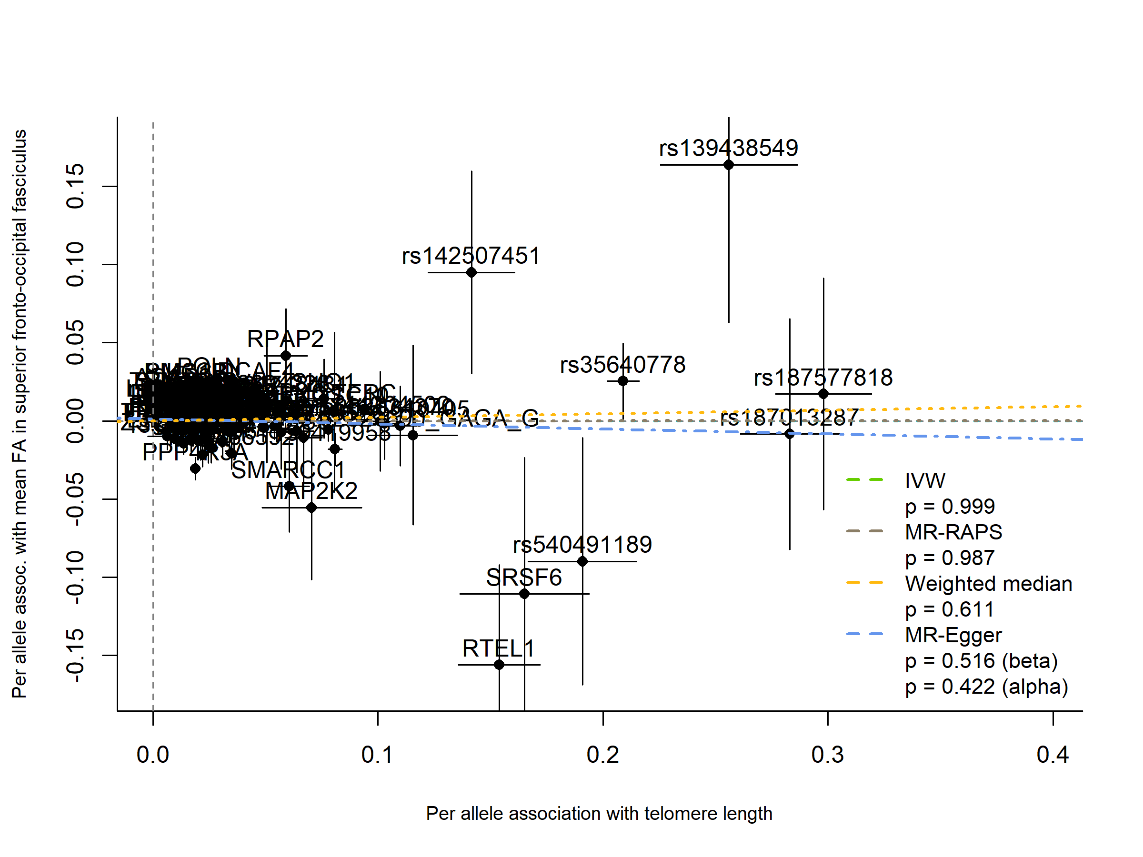


**Figure S52.** SNP-mean FA in superior fronto-occipital fasciculus association plotted against SNP-telomere length association, labelled by the mapped gene, with MR slope estimates shown


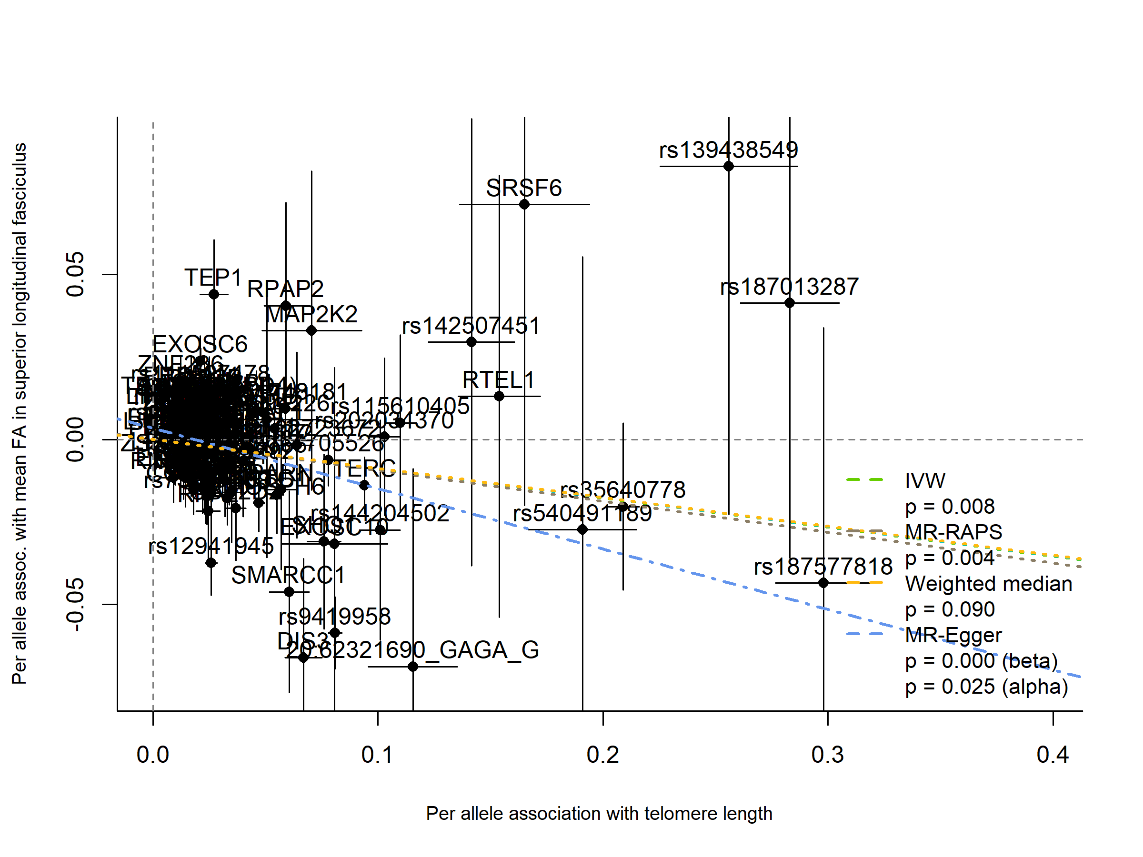


**Figure S53.** SNP-mean FA in superior longitudinal fasciculus association plotted against SNP-telomere length association, labelled by the mapped gene, with MR slope estimates shown


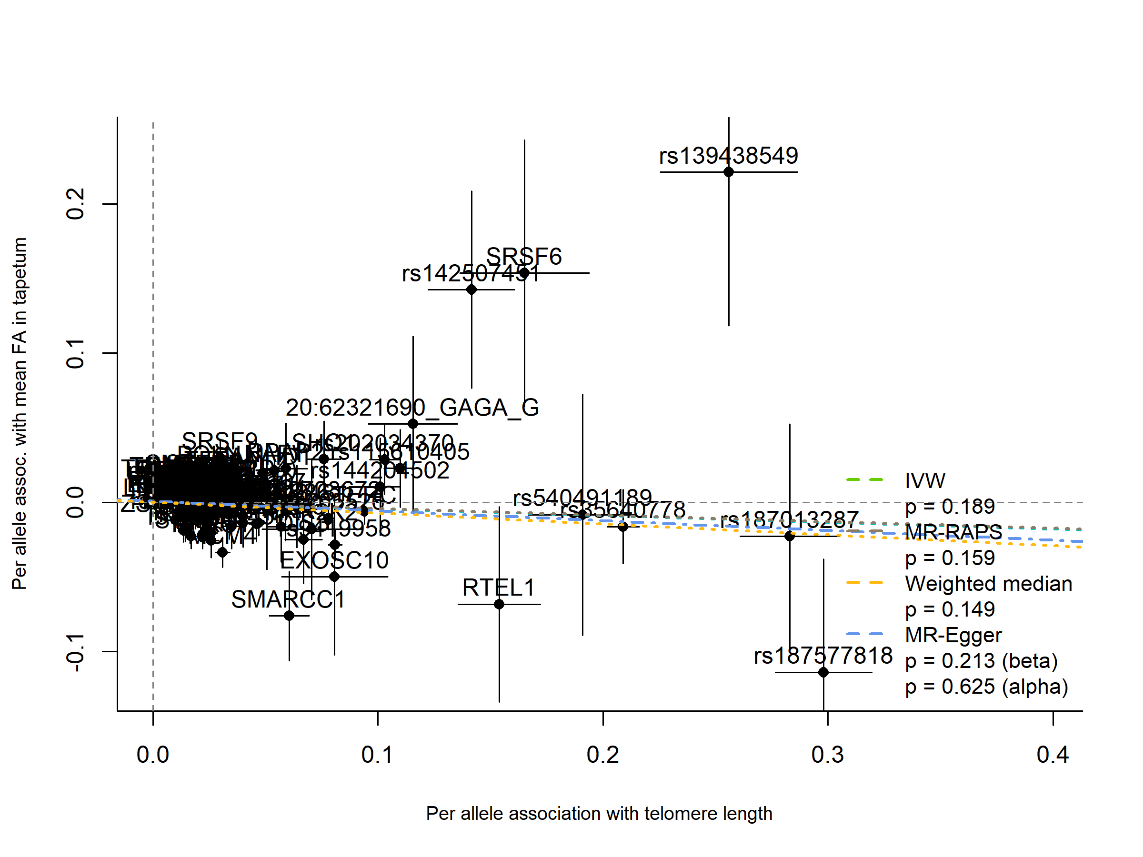


**Figure S54.** SNP-mean FA in tapetum association plotted against SNP-telomere length association, labelled by the mapped gene, with MR slope estimates shown


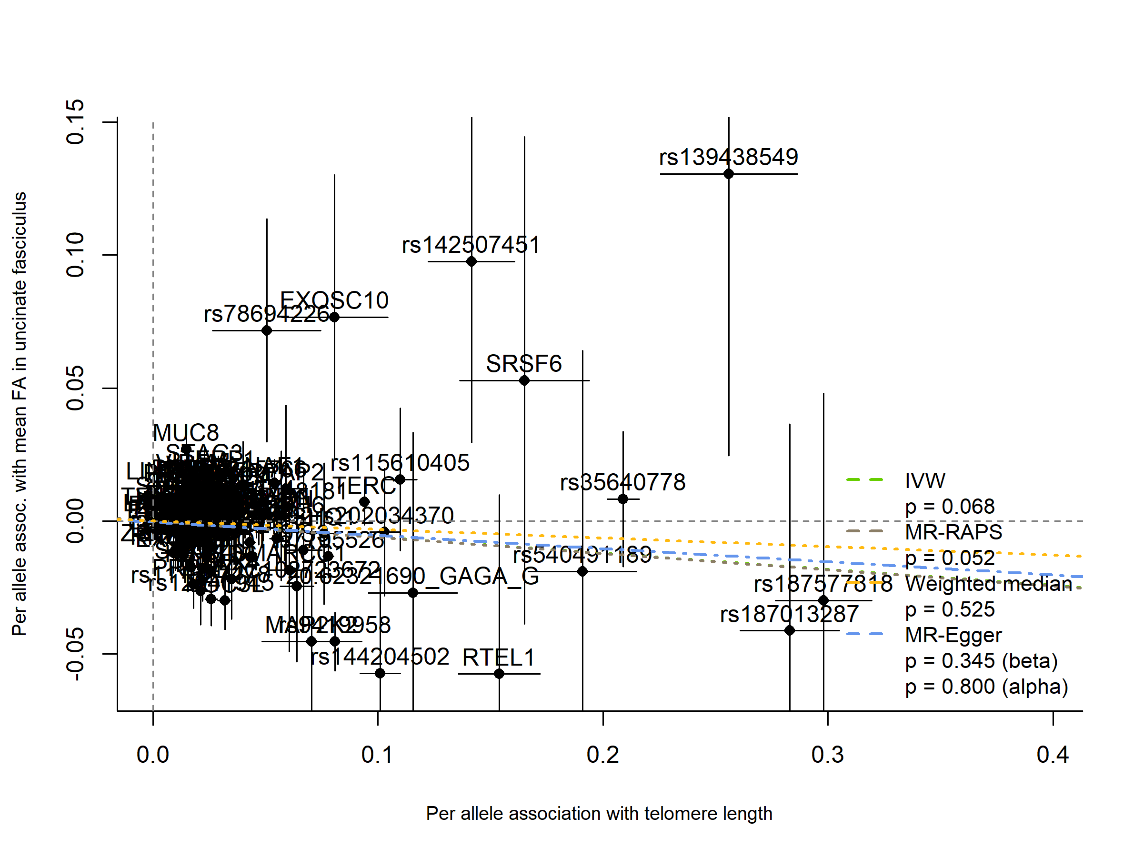


**Figure S55.** SNP-mean FA in uncinate fasciculus association plotted against SNP-telomere length association, labelled by the mapped gene, with MR slope estimates shown

**
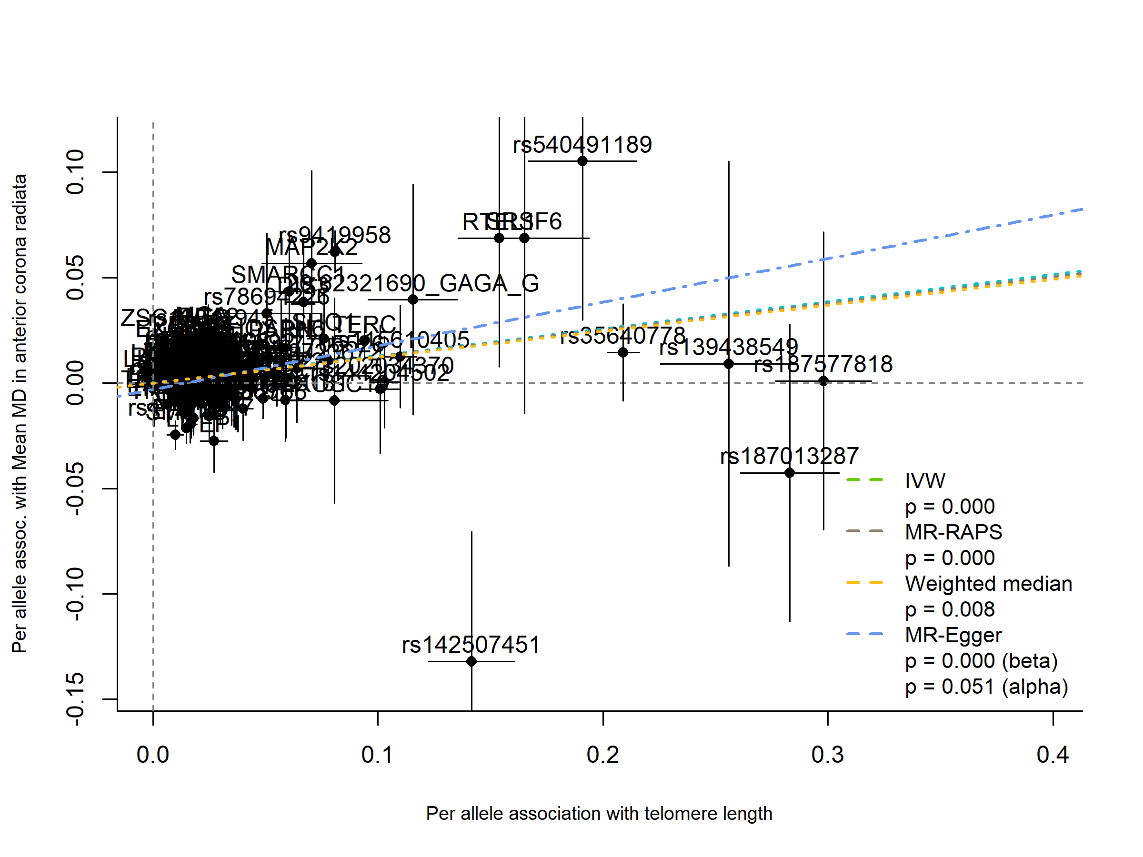
**

**Figure S56.** SNP-mean MD in anterior corona radiata association plotted against SNP-telomere length association, labelled by the mapped gene, with MR slope estimates shown


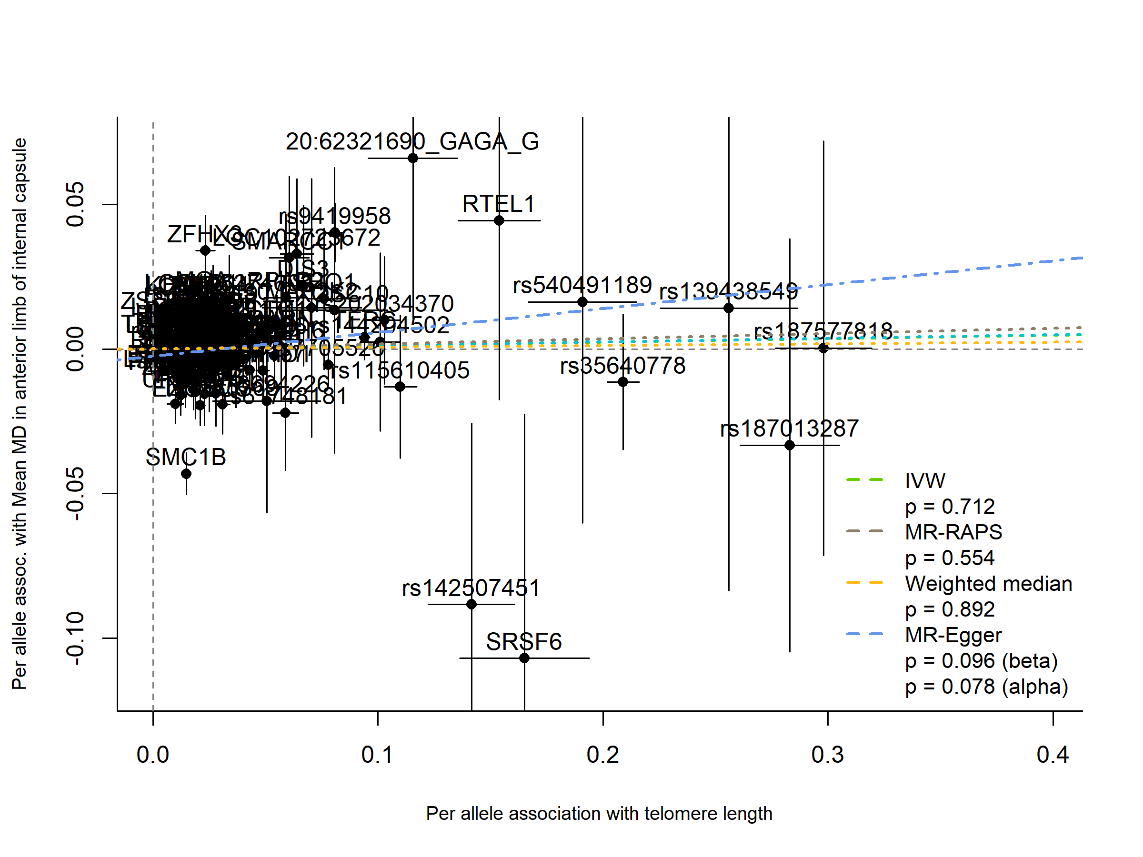


**Figure S57.** SNP-mean MD in anterior limb of internal capsule association plotted against SNP-telomere length association, labelled by the mapped gene, with MR slope estimates shown


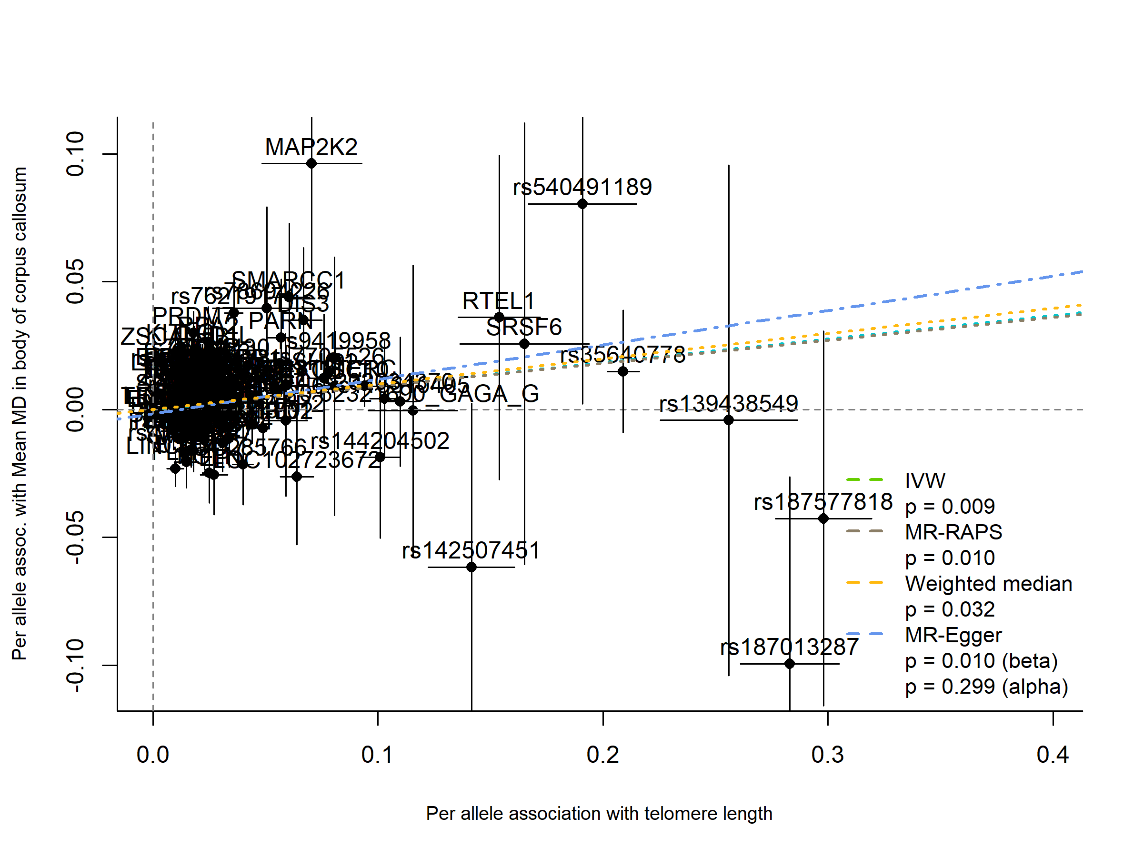


**Figure S58.** SNP-mean MD in body of corpus callosum association plotted against SNP-telomere length association, labelled by the mapped gene, with MR slope estimates shown


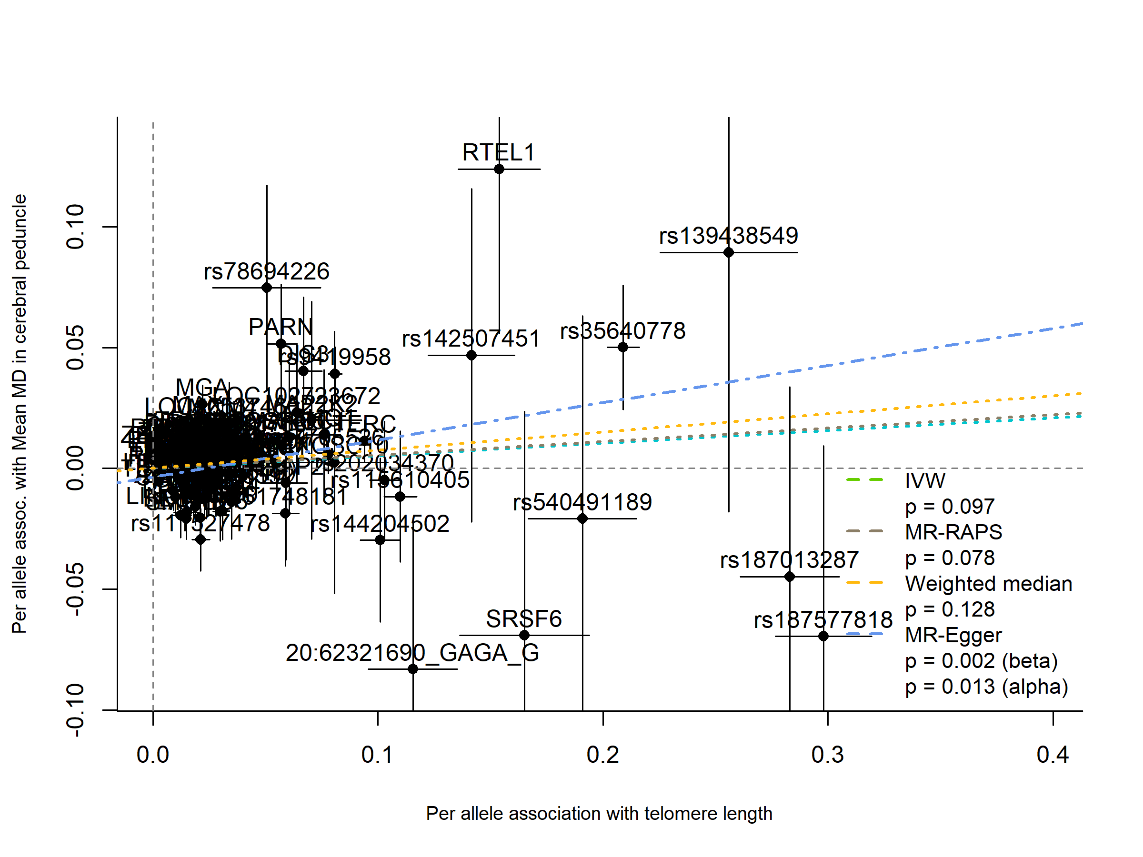


**Figure S59.** SNP-mean MD in cerebral peduncle association plotted against SNP-telomere length association, labelled by the mapped gene, with MR slope estimates shown


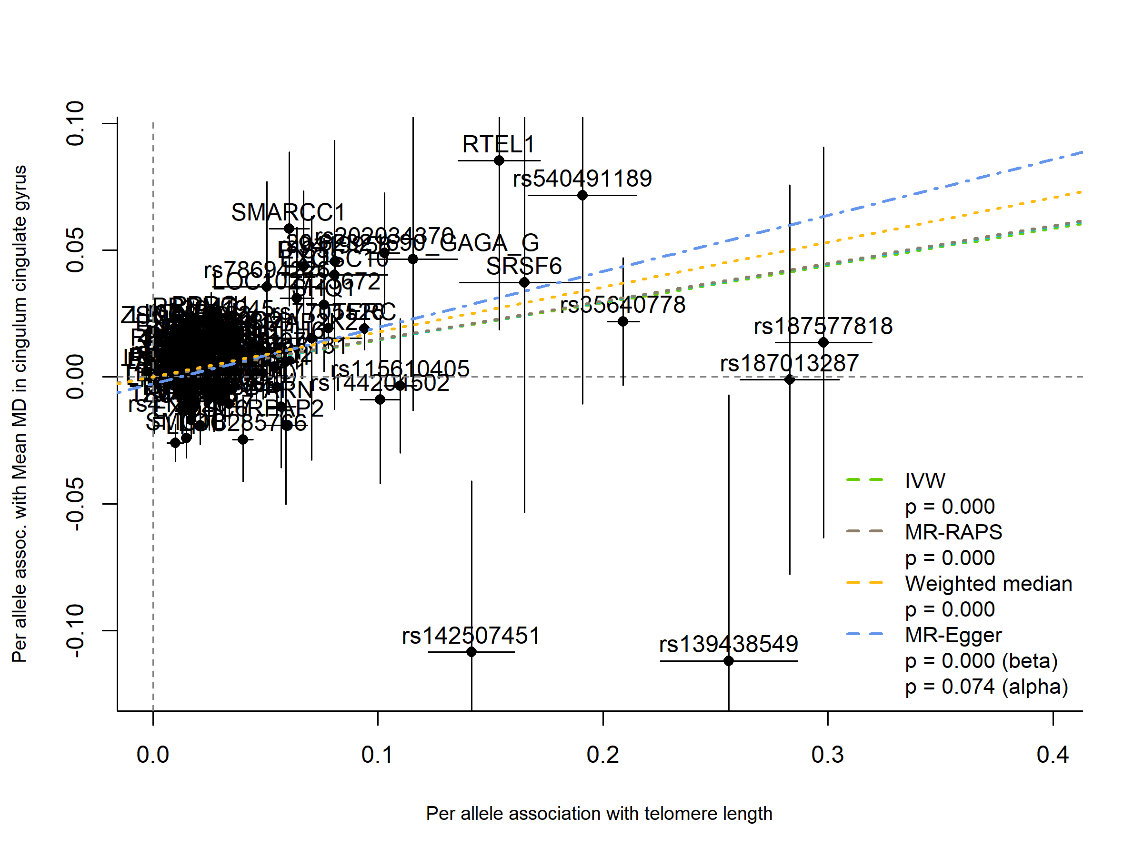


**Figure S60.** SNP-mean MD in cingulum cingulate gyrus association plotted against SNP-telomere length association, labelled by the mapped gene, with MR slope estimates shown


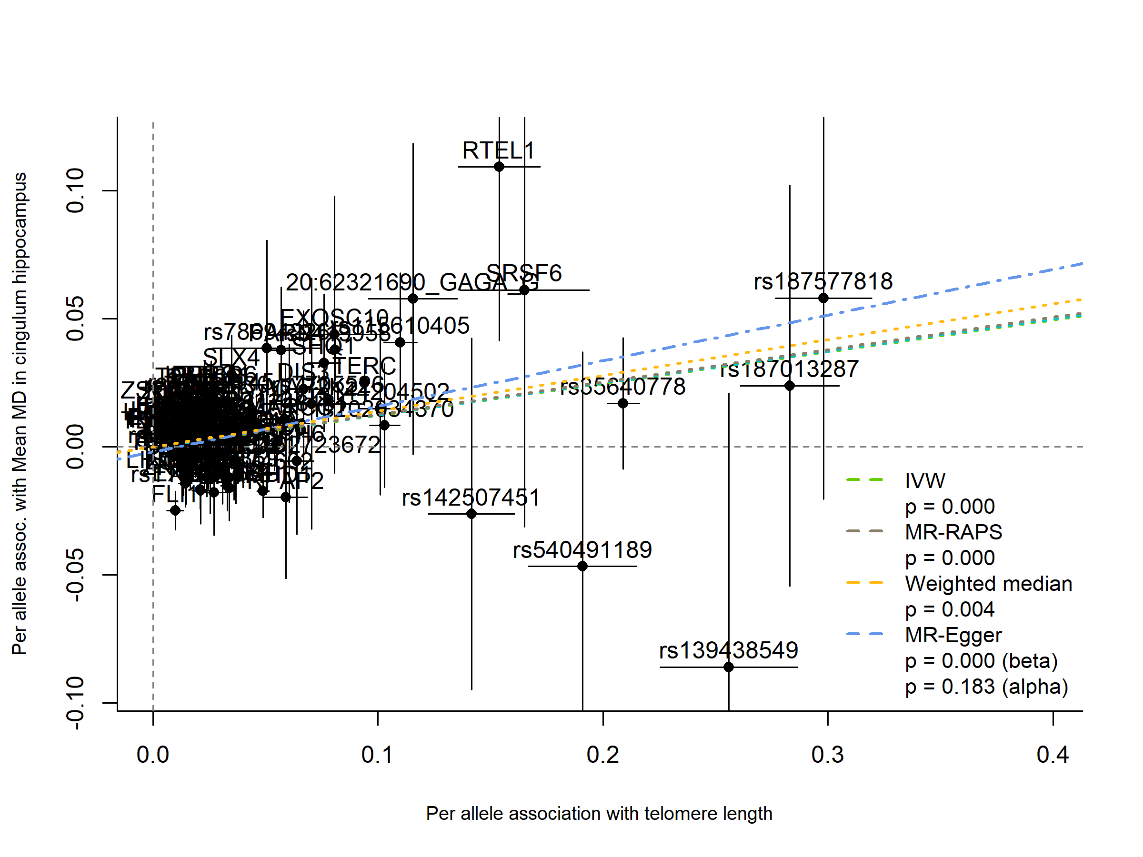


**Figure S61.** SNP-mean MD in cingulum hippocampus association plotted against SNP-telomere length association, labelled by the mapped gene, with MR slope estimates shown


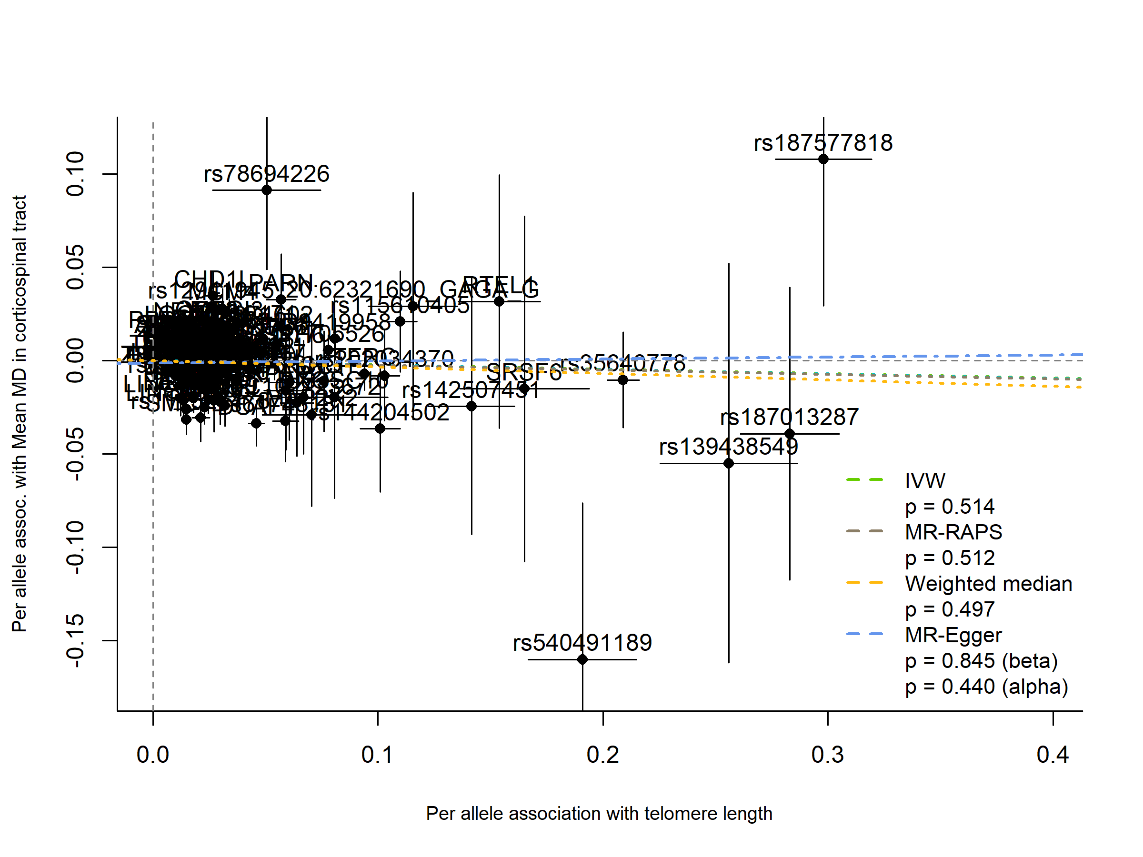


**Figure S62.** SNP-mean MD in corticospinal tract association plotted against SNP-telomere length association, labelled by the mapped gene, with MR slope estimates shown


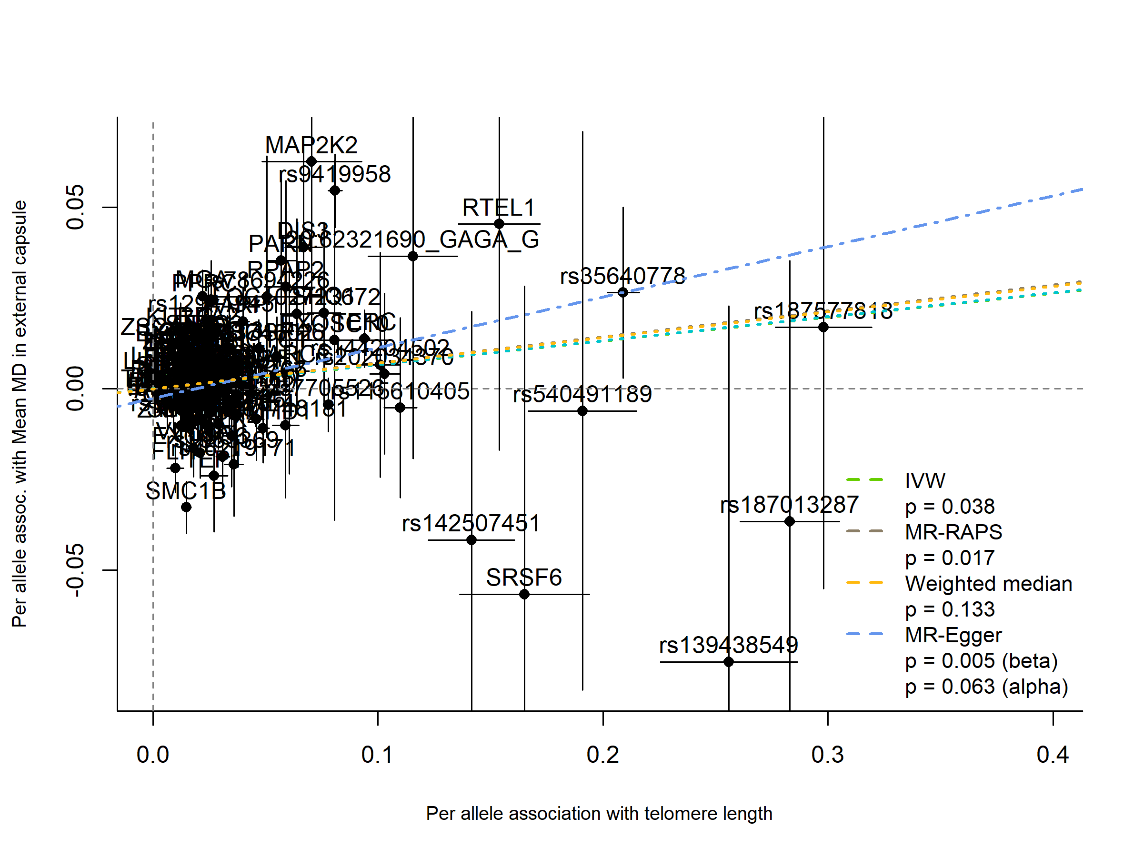


**Figure S63.** SNP-mean MD in external capsule association plotted against SNP-telomere length association, labelled by the mapped gene, with MR slope estimates shown


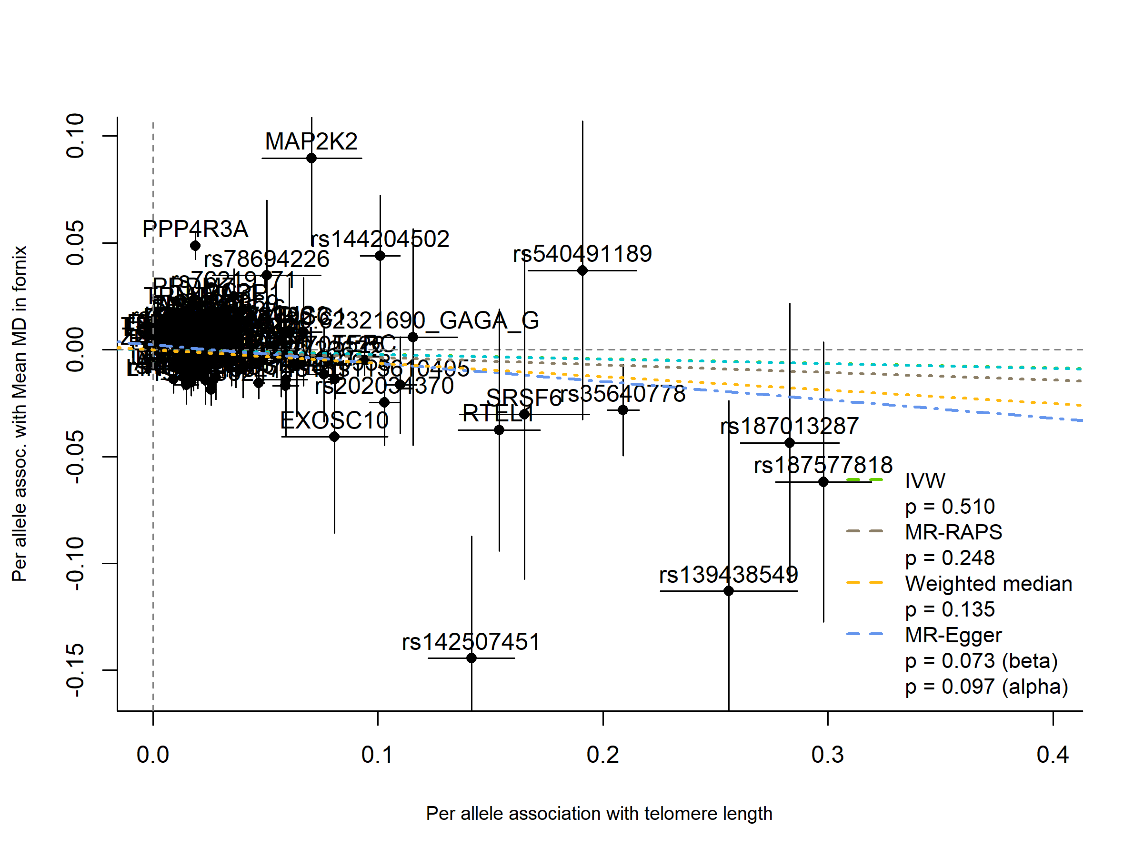


**Figure S64.** SNP-mean MD in fornix association plotted against SNP-telomere length association, labelled by the mapped gene, with MR slope estimates shown


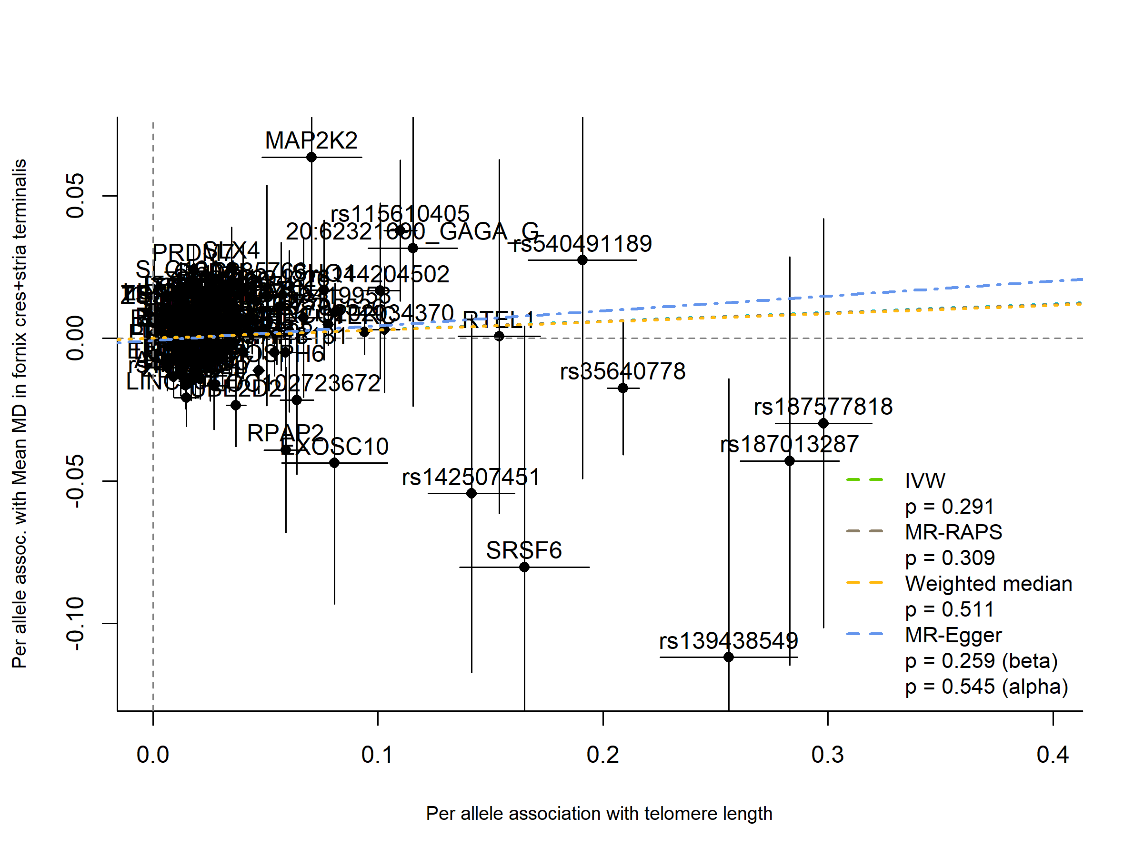


**Figure S65.** SNP-mean MD in fornix cres+stria terminalis association plotted against SNP-telomere length association, labelled by the mapped gene, with MR slope estimates shown


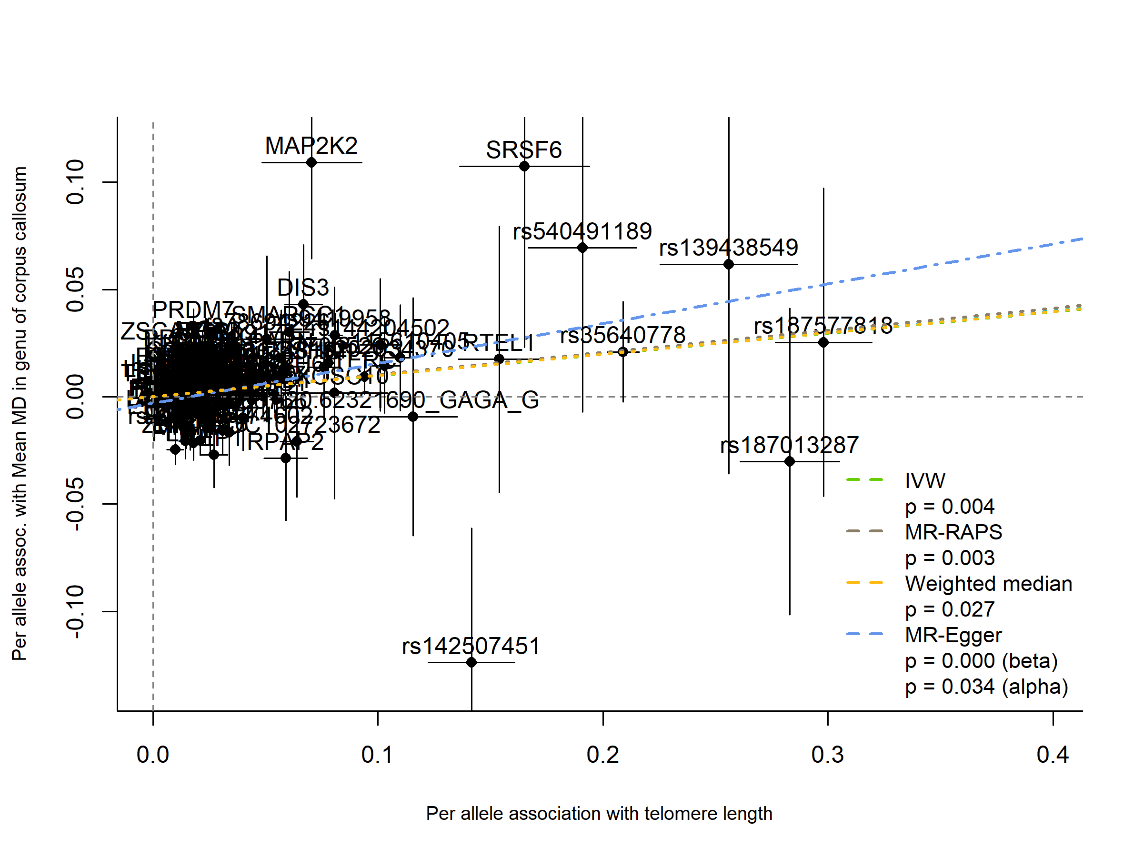


**Figure S66.** SNP-mean MD in genu of corpus callosum association plotted against SNP-telomere length association, labelled by the mapped gene, with MR slope estimates shown


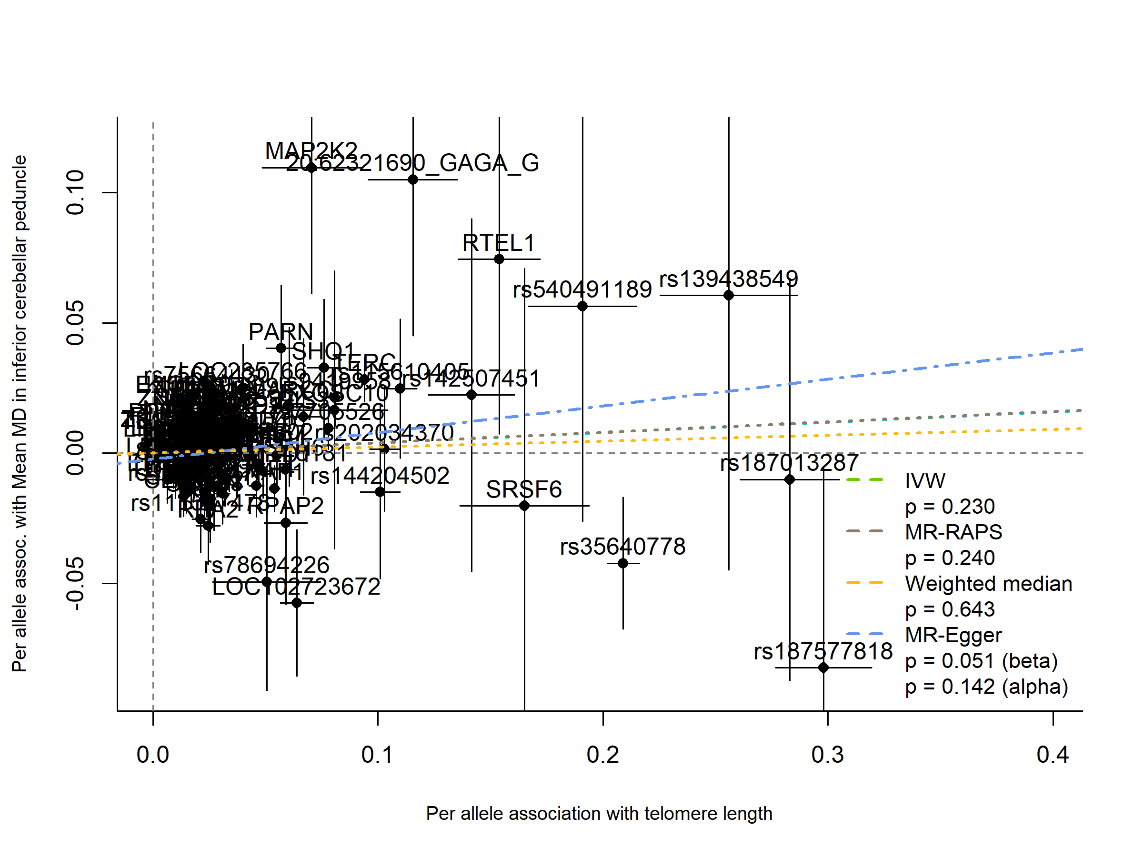


**Figure S67.** SNP-mean MD in inferior cerebellar peduncle association plotted against SNP-telomere length association, labelled by the mapped gene, with MR slope estimates shown


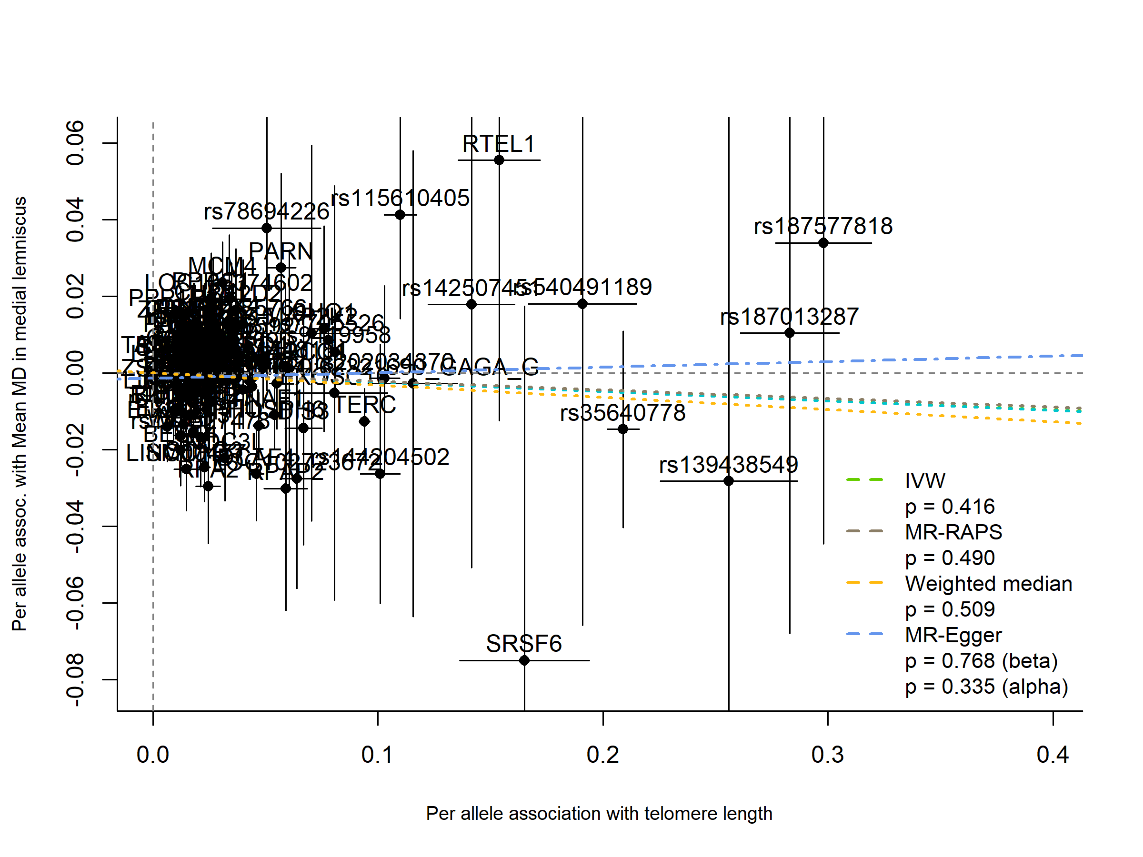


**Figure S68.** SNP-mean MD in medial lemniscus association plotted against SNP-telomere length association, labelled by the mapped gene, with MR slope estimates shown


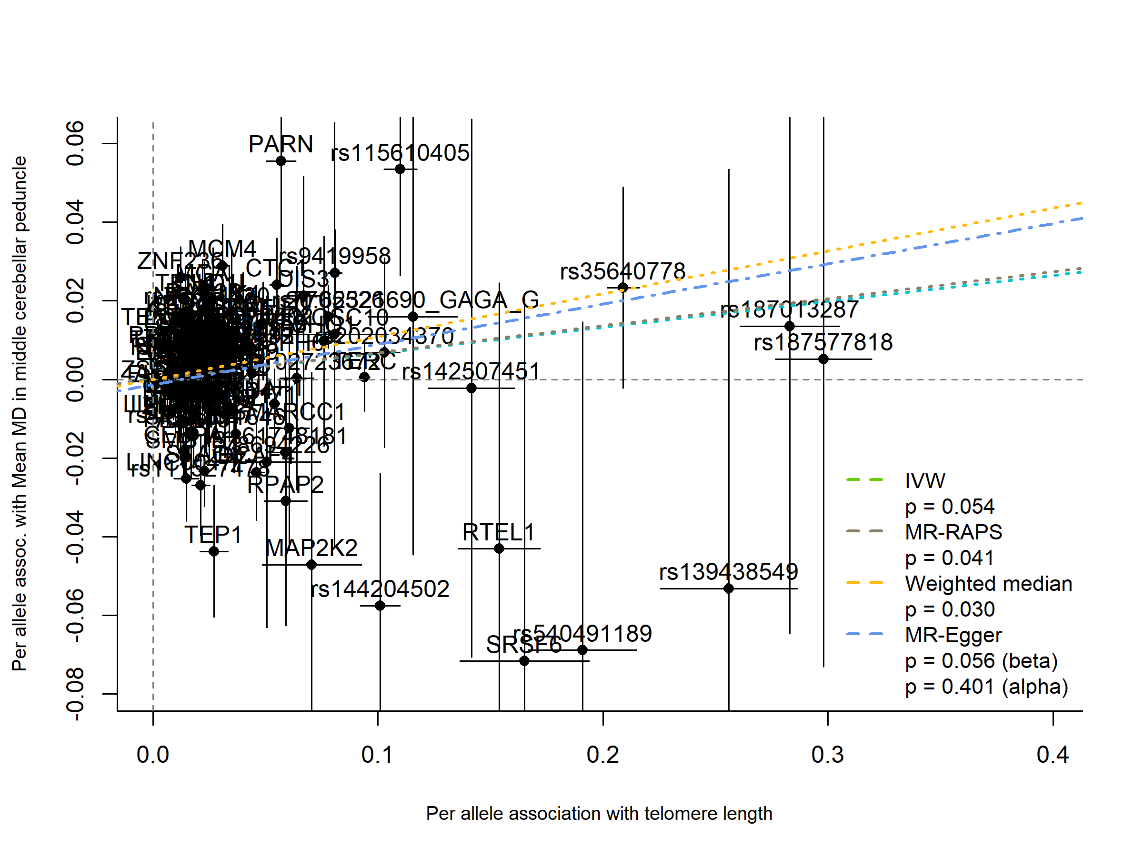


**Figure S69.** SNP-mean MD in middle cerebellar peduncle association plotted against SNP-telomere length association, labelled by the mapped gene, with MR slope estimates shown


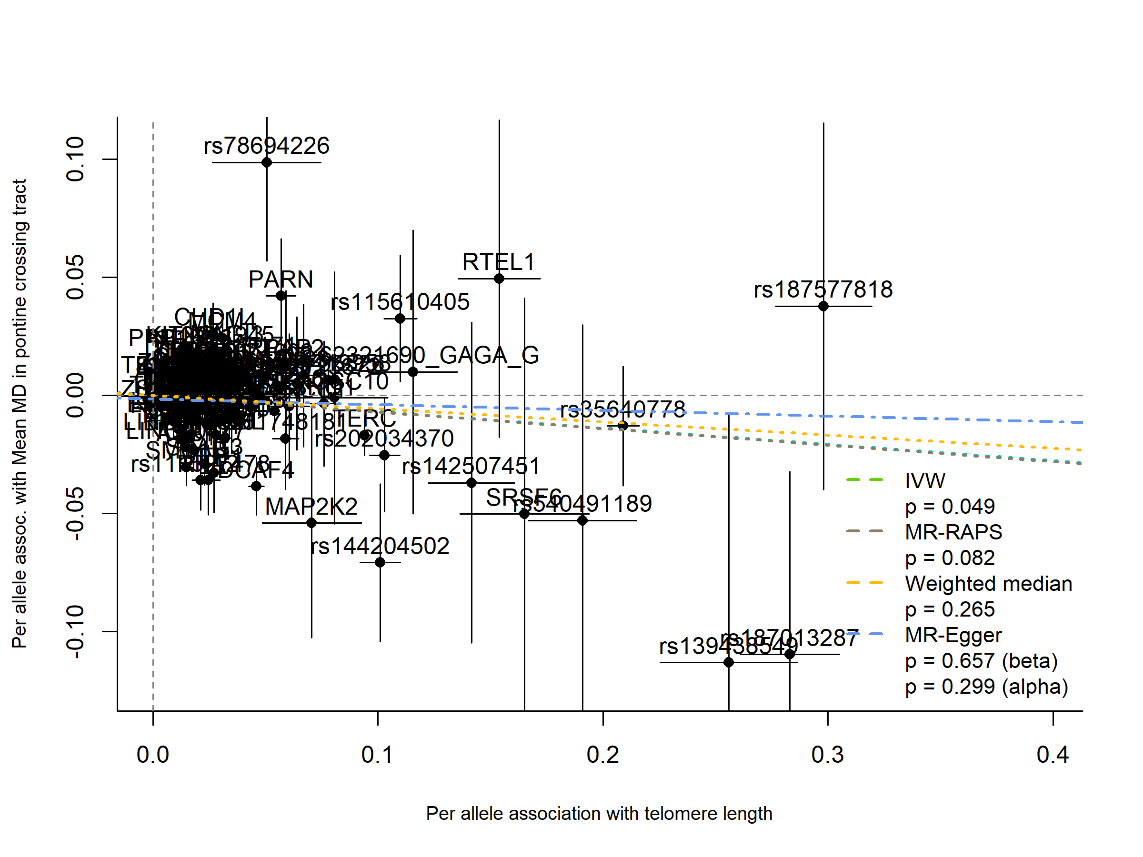


**Figure S70.** SNP-mean MD in pontine crossing tract association plotted against SNP-telomere length association, labelled by the mapped gene, with MR slope estimates shown


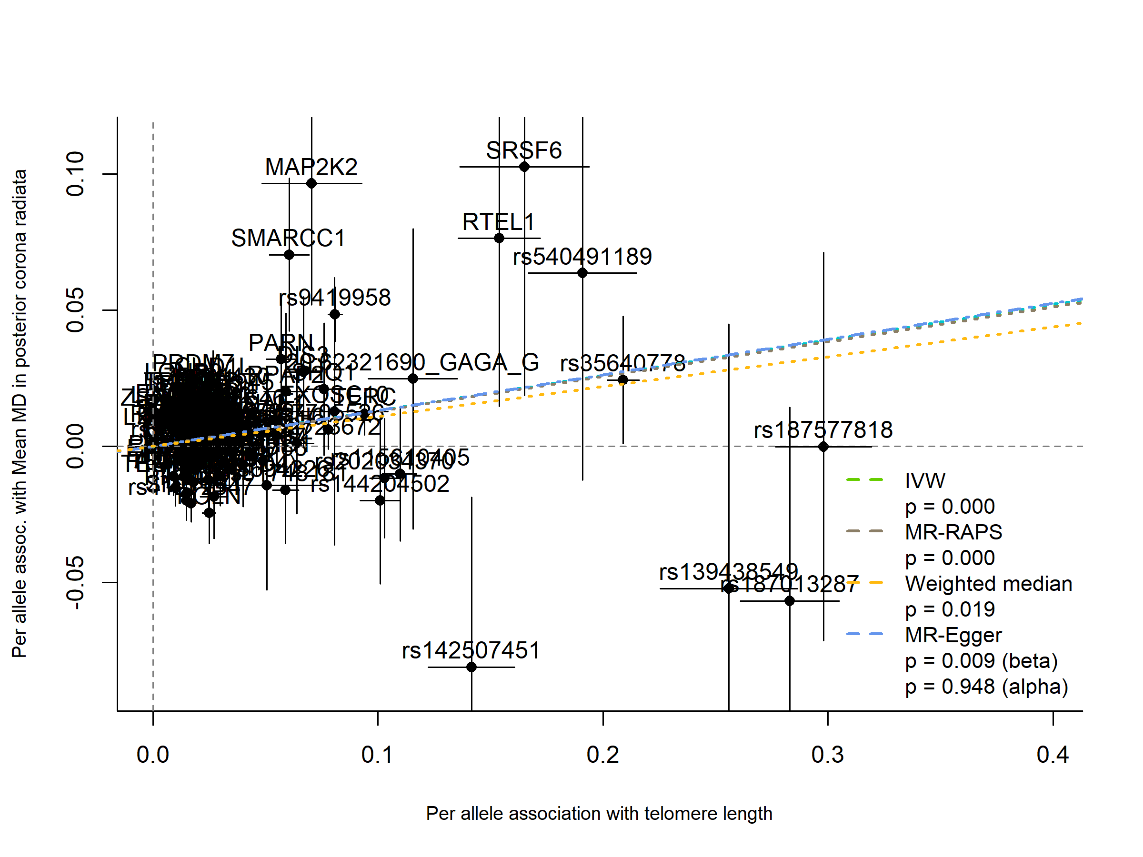


**Figure S71.** SNP-mean MD in posterior corona radiata association plotted against SNP-telomere length association, labelled by the mapped gene, with MR slope estimates shown


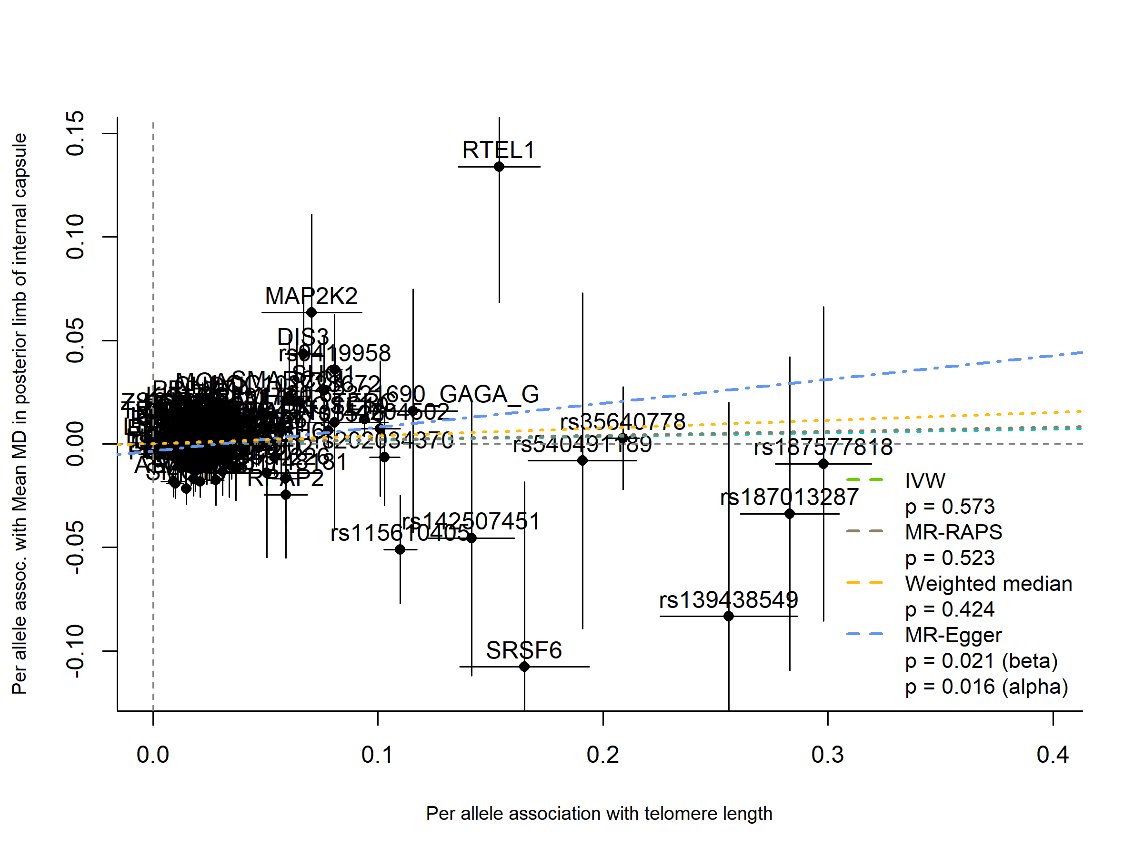


**Figure S72.** SNP-mean MD in posterior limb of internal capsule association plotted against SNP-telomere length association, labelled by the mapped gene, with MR slope estimates shown


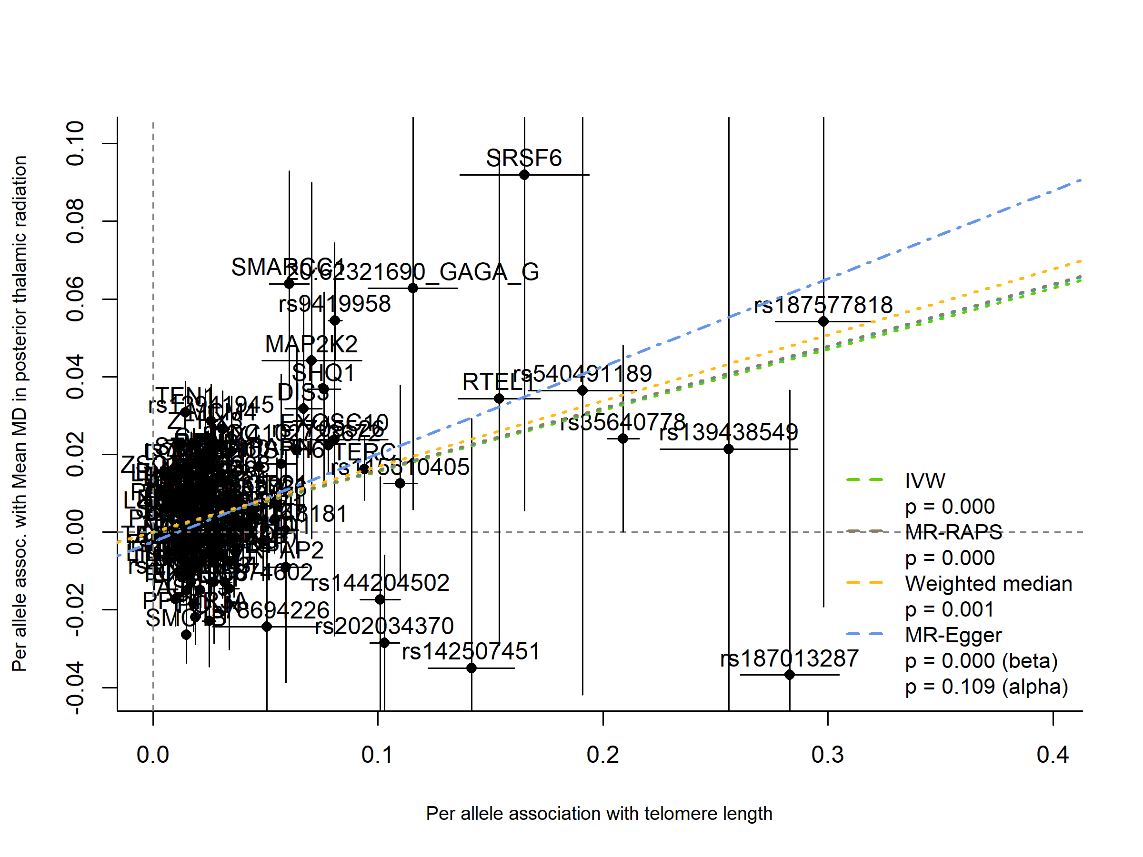


**Figure S73.** SNP-mean MD in posterior thalamic radiation association plotted against SNP-telomere length association, labelled by the mapped gene, with MR slope estimates shown


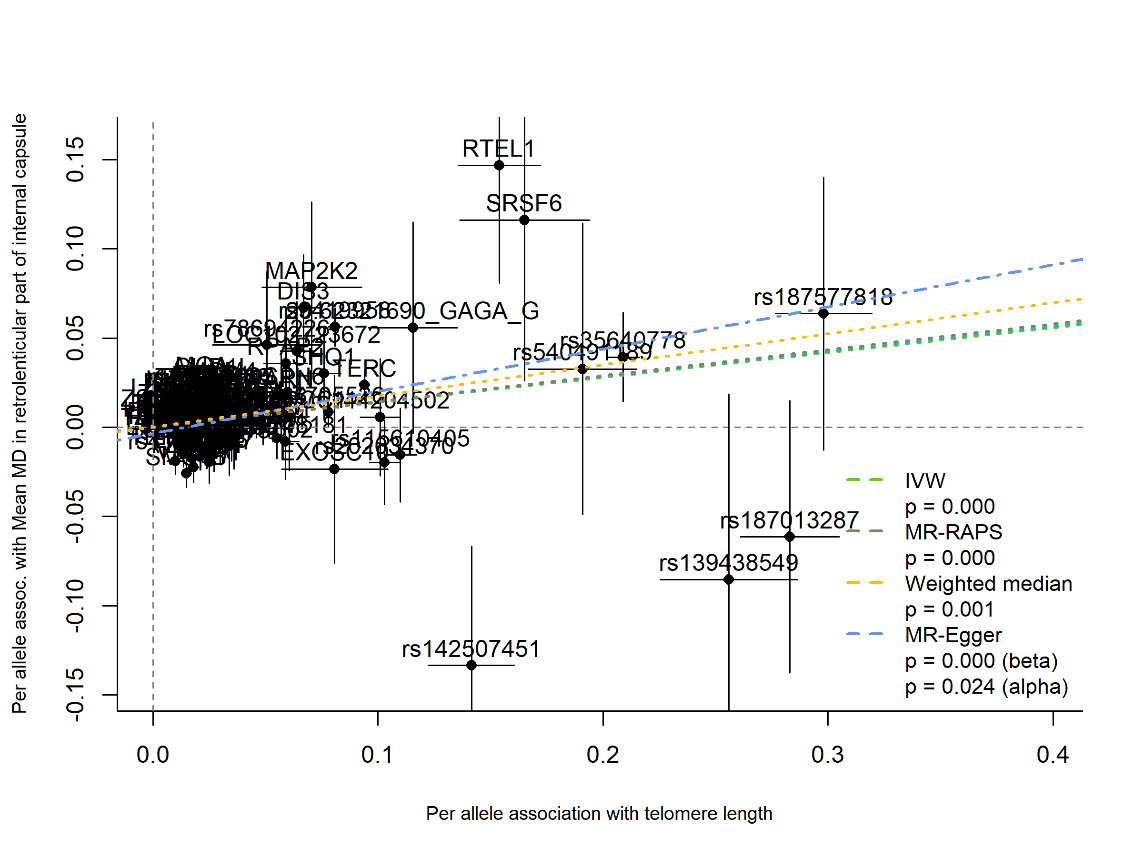


**Figure S74.** SNP-mean MD in retrolenticular part of internal capsule association plotted against SNP-telomere length association, labelled by the mapped gene, with MR slope estimates shown


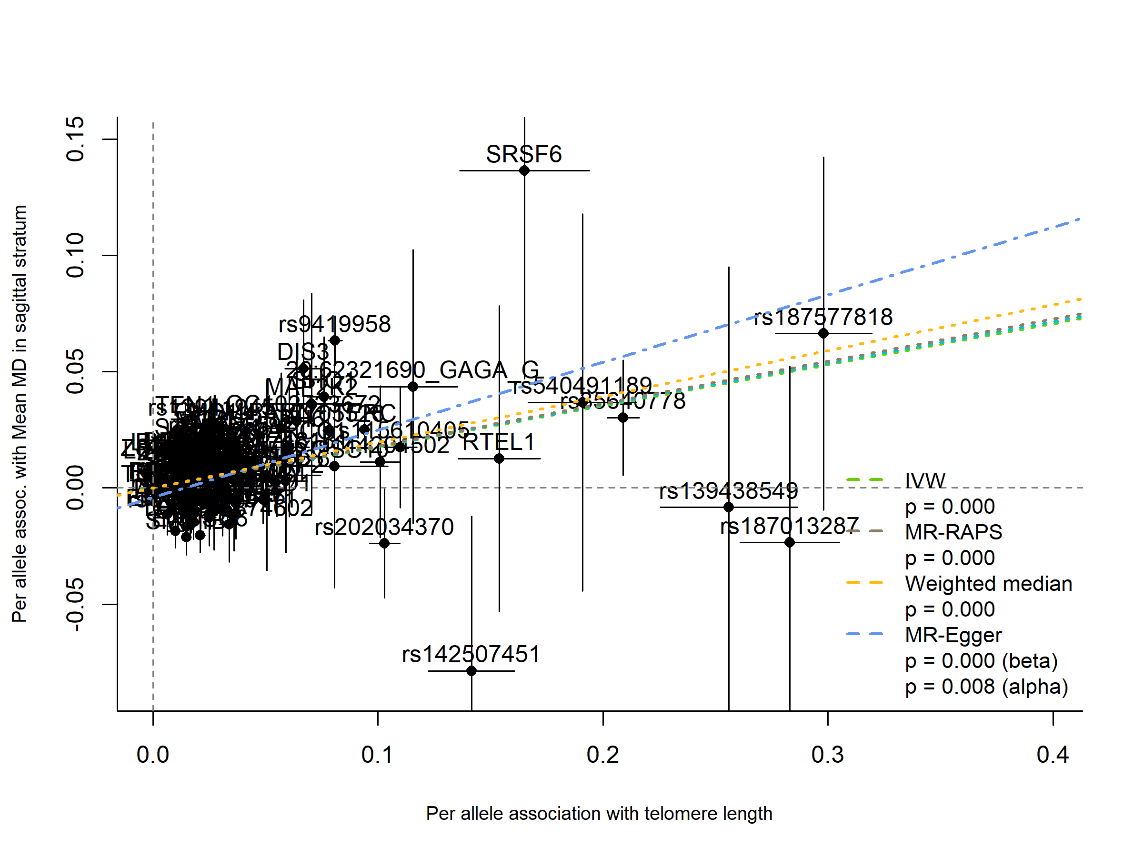


**Figure S75.** SNP-mean MD in sagittal stratum association plotted against SNP-telomere length association, labelled by the mapped gene, with MR slope estimates shown


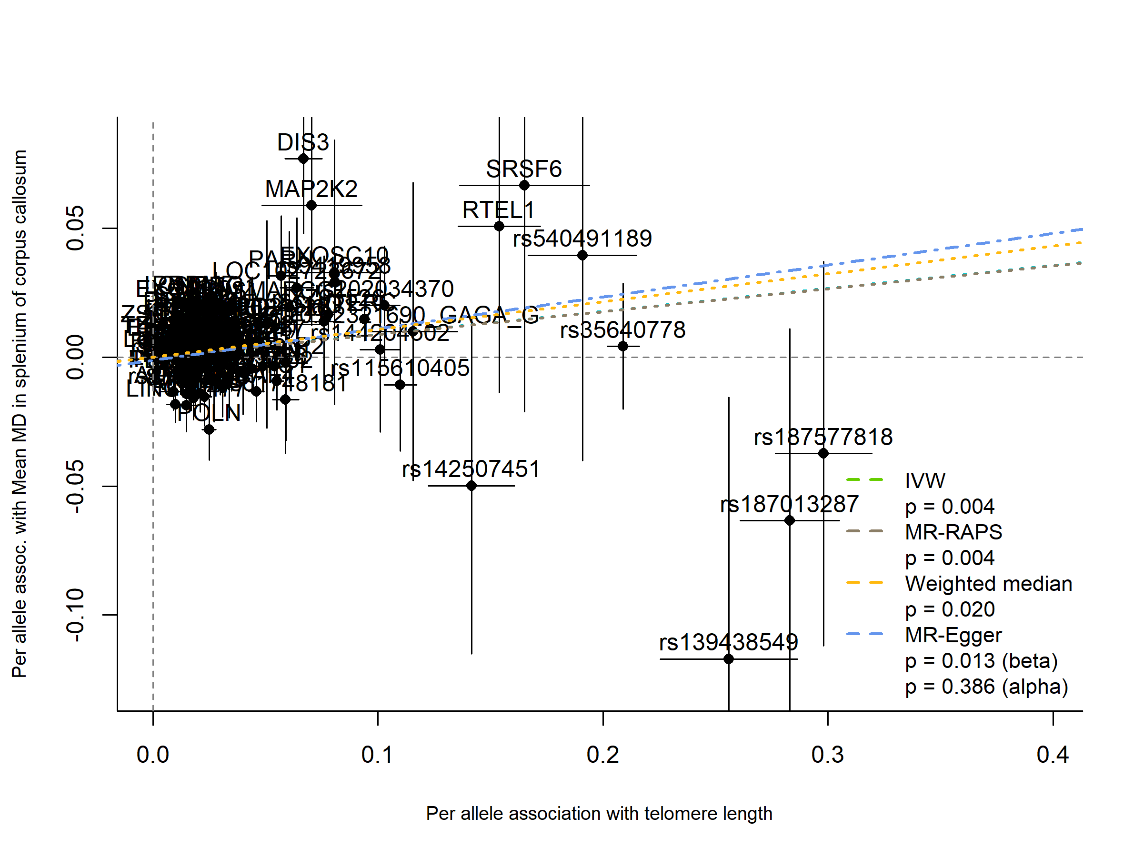


**Figure S76.** SNP-mean MD in splenium of corpus callosum association plotted against SNP-telomere length association, labelled by the mapped gene, with MR slope estimates shown


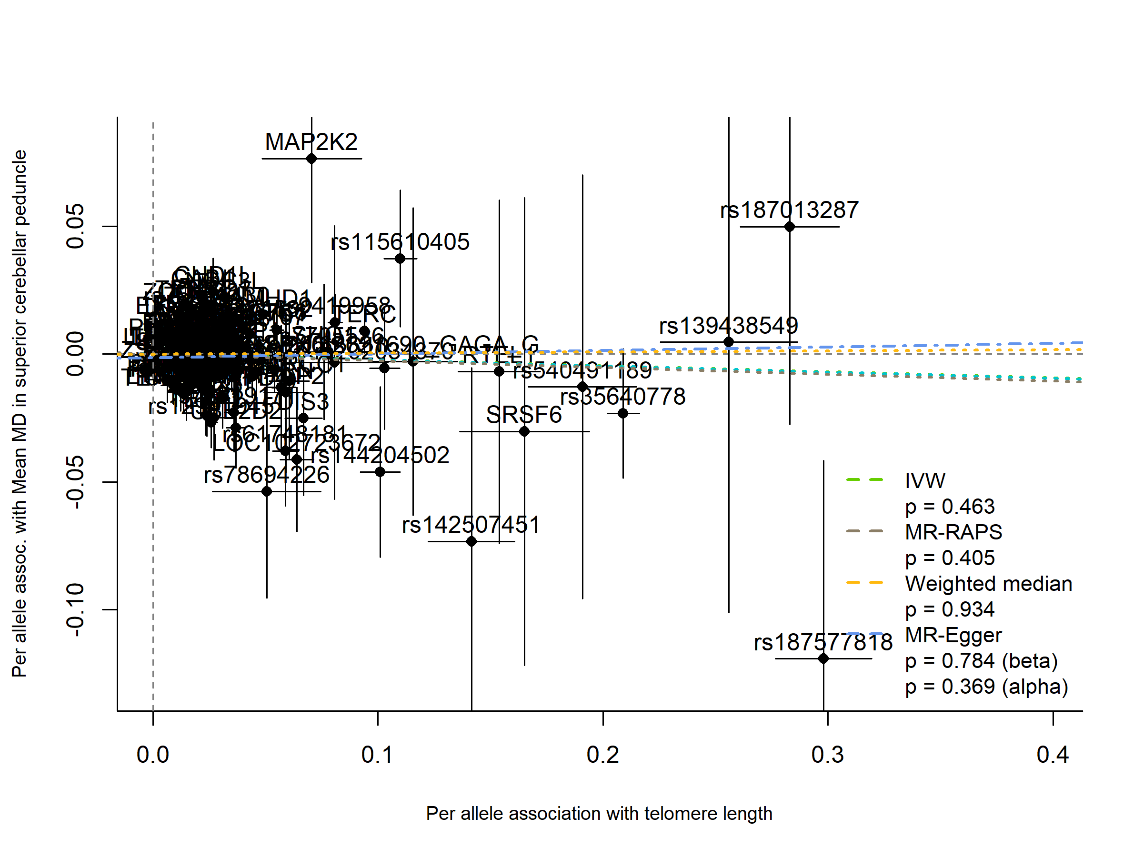


**Figure S77.** SNP-mean MD in superior cerebellar peduncle association plotted against SNP-telomere length association, labelled by the mapped gene, with MR slope estimates shown


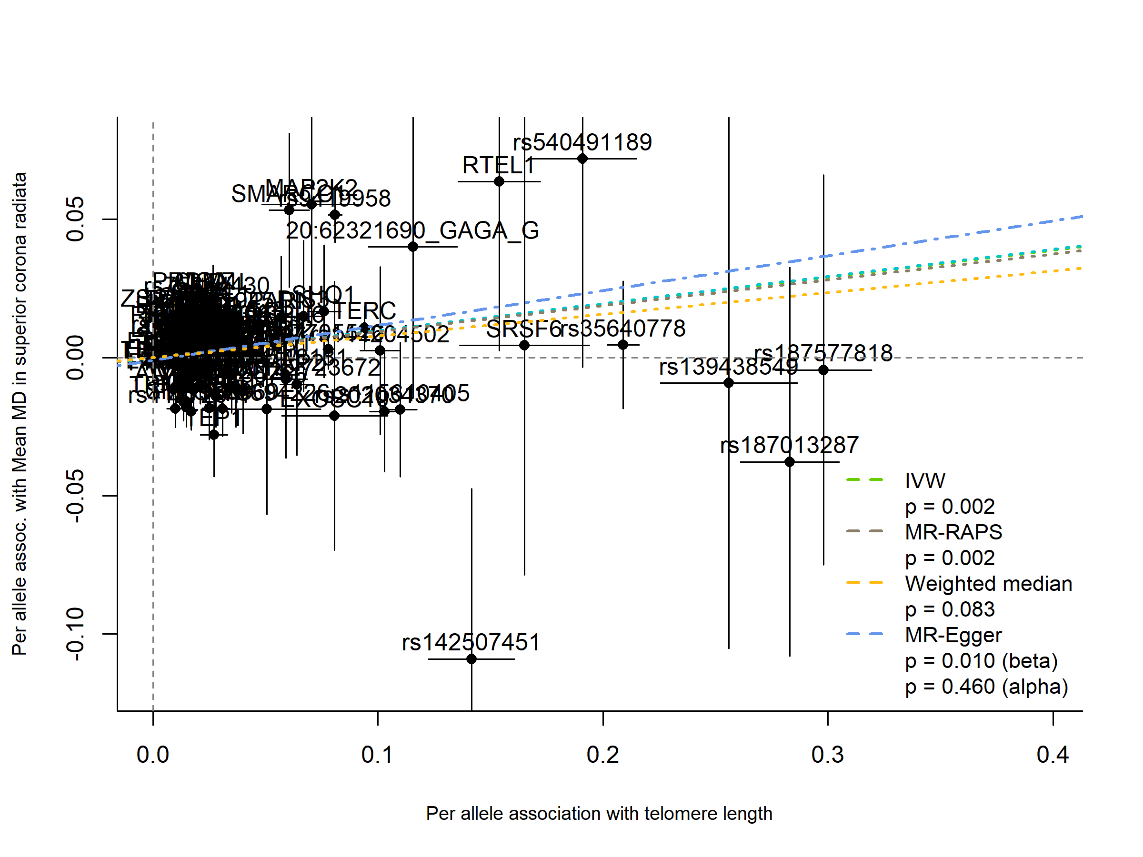


**Figure S78.** SNP-mean MD in superior corona radiata association plotted against SNP-telomere length association, labelled by the mapped gene, with MR slope estimates shown


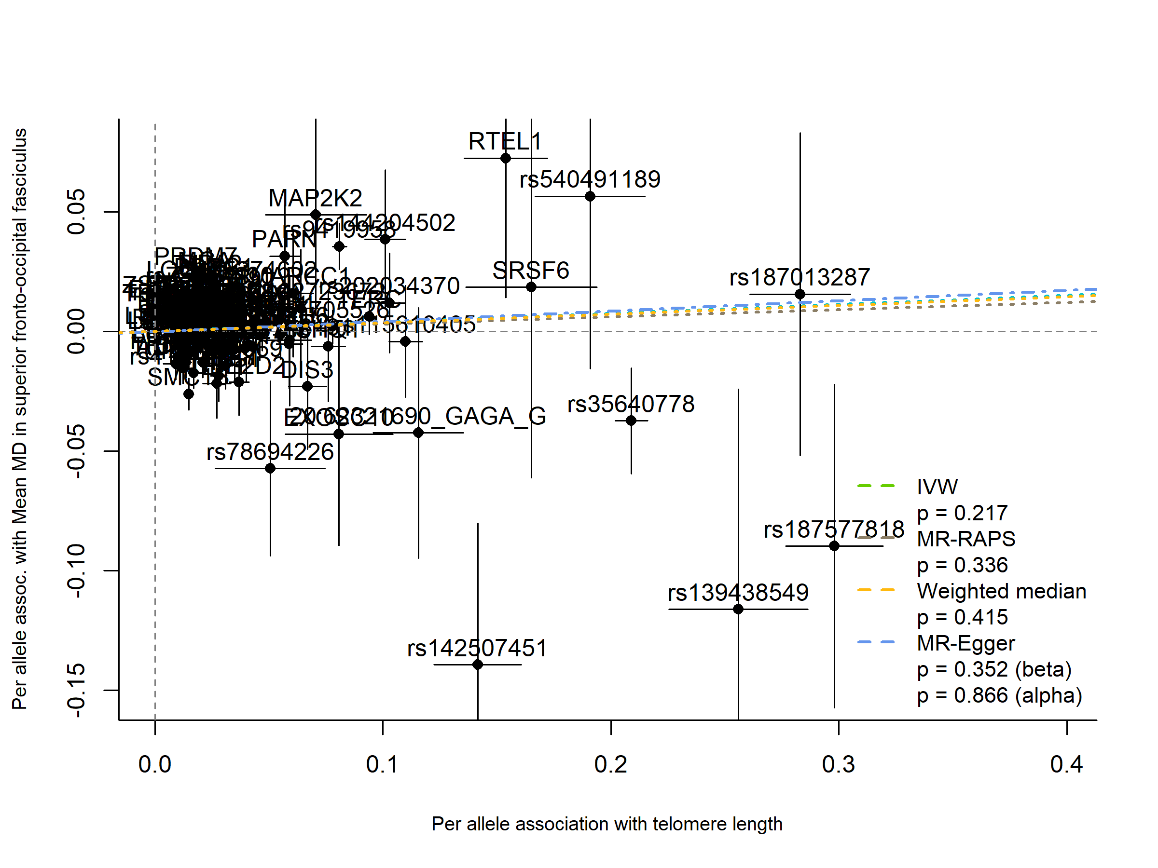


**Figure S79.** SNP-mean MD in superior fronto-occipital fasciculus association plotted against SNP-telomere length association, labelled by the mapped gene, with MR slope estimates shown


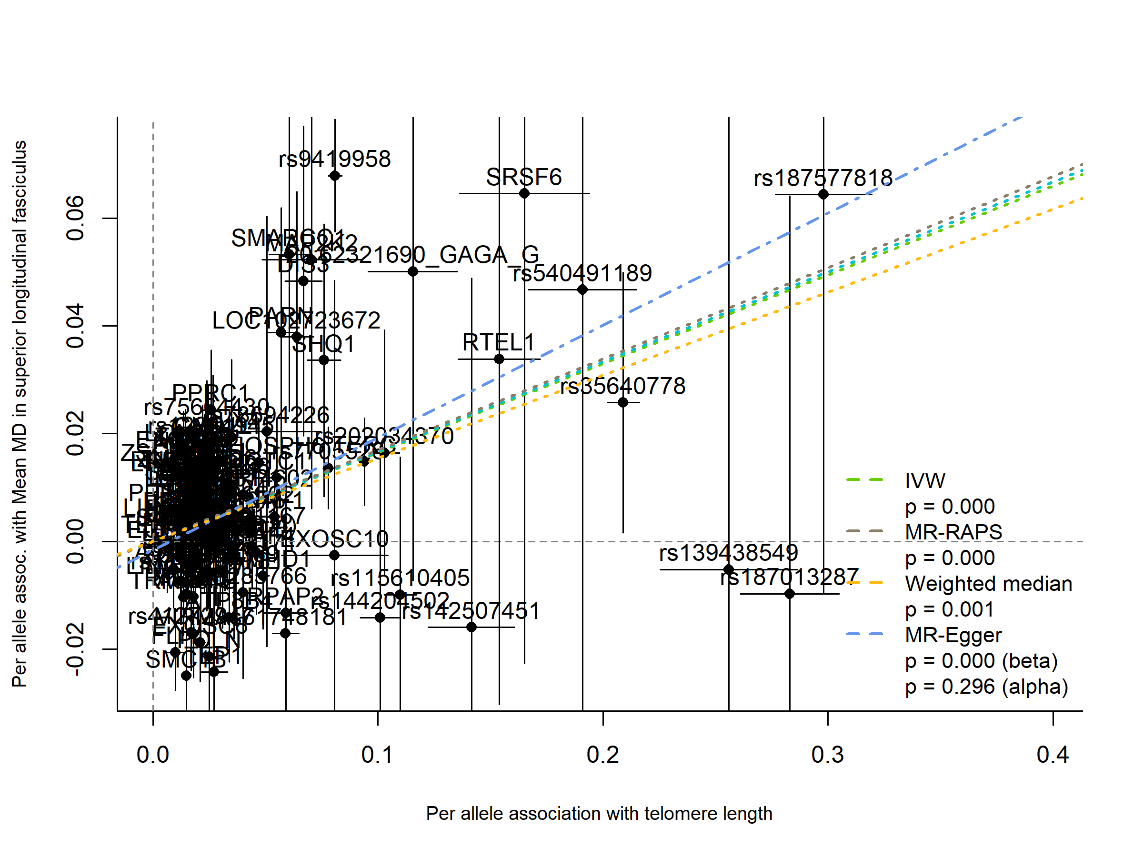


**Figure S80.** SNP-mean MD in superior longitudinal fasciculus association plotted against SNP-telomere length association, labelled by the mapped gene, with MR slope estimates shown


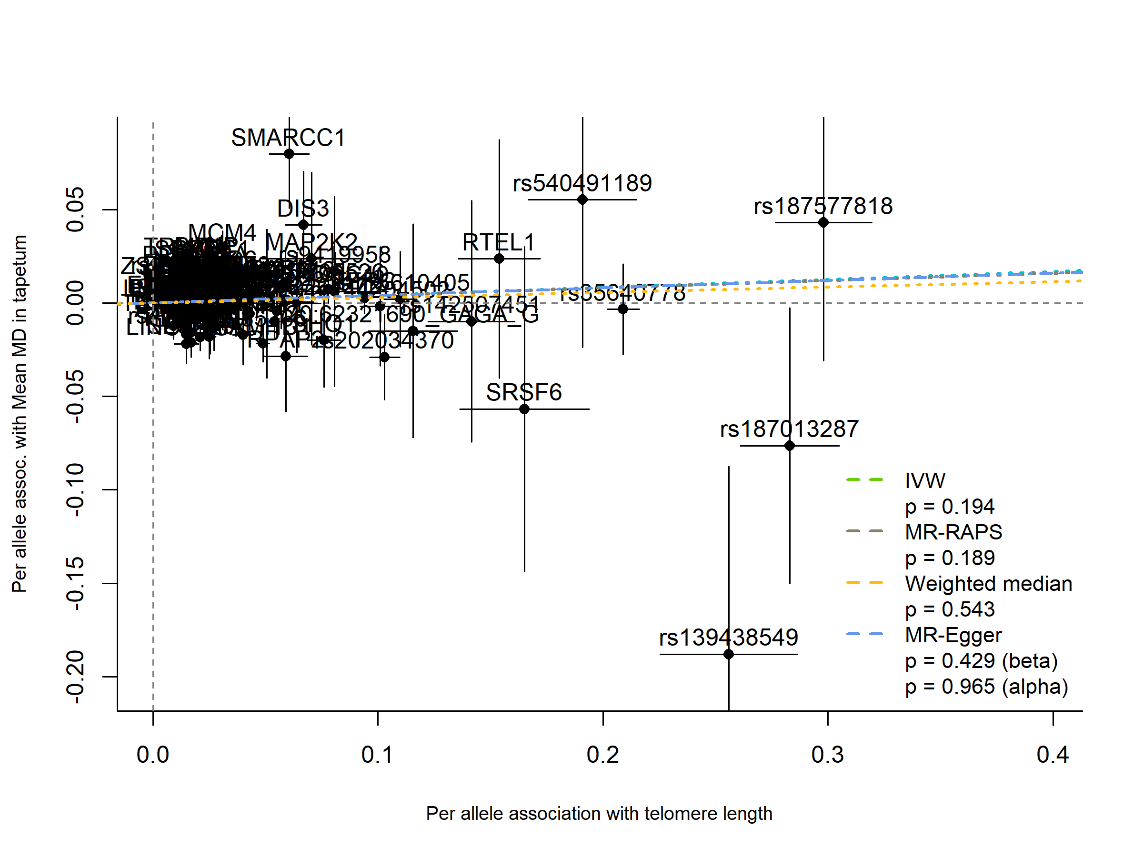


**Figure S81.** SNP-mean MD in tapetum association plotted against SNP-telomere length association, labelled by the mapped gene, with MR slope estimates shown


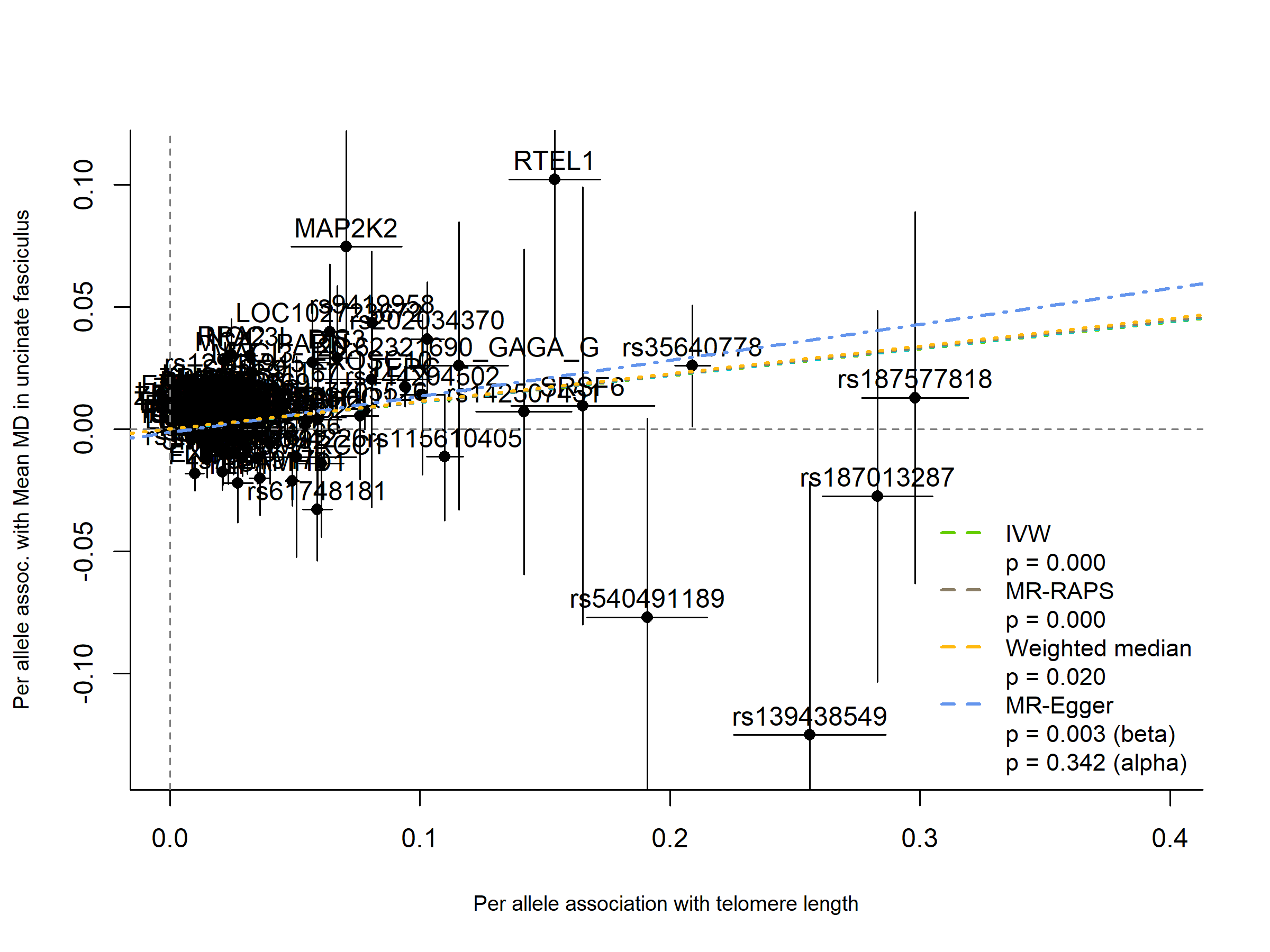


**Figure S82.** SNP-mean MD in uncinate fasciculus association plotted against SNP-telomere length association, labelled by the mapped gene, with MR slope estimates shown
