# Supplementary material for: Mid‐life leukocyte telomere length and dementia risk: An observational and mendelian randomization study of 435,046 UK Biobank participants
Source: Aging Cell. 2023 May 30;22(7):e13808. doi: 10.1111/acel.13808 (PMC10352557; doi:10.1111/acel.13808)
Supplement: Supplementary file 3 — TablesS2‐S8 [file ACEL-22-e13808-s004.docx]

**Tables S2-S8**

**Mid-life leukocyte telomere length and dementia risk: an observational and Mendelian randomization study of 435,046 UK Biobank participants**

**Table S2** | UKB field IDs and ICD-10 codes for identification of AD/ADRD

**Table S3** | A selection of UK Biobank cognitive tests

**Table S4** | Selected imaging derived phenotypes

**Table S5** | Baseline characteristics of participants in the baseline or imaging cohort

**Table S6** | Participant characteristics at baseline of incident AD/ADRD cases versus normal controls

**Table S7** | Participant characteristics at baseline of incident Alzheimer’s disease (AD) cases versus normal controls

**Table S8 |** Participant characteristics at baseline of incident vascular dementia (VD) cases versus normal controls

**Table S2. UKB field IDs and ICD-10 codes for identification of AD/ADRD**

| **AD/ADRD** | **UKB field ID** | **ICD-10** | **Data release period currently available in England*** |
| --- | --- | --- | --- |
| Alzheimer’s disease (AD) | 131036 | G30 | Death data: April 2006-February 2021  Hospital inpatient data: 1997-March 2021 for half of the UKB cohort  Primary care data: 1938-2017  Self-reported data: initial assessment visit (2006-2010), first repeat assessment visit (2012-2013), imaging visit (2014+), first repeat imaging visit (2019+) |
| Dementia in AD | 130836 | F00 |  |
| Vascular dementia | 130838 | F01 |  |
| Unspecified dementia | 130842 | F03 |  |
| Other degenerative diseases of nervous system, not elsewhere classified, including frontotemporal dementia and Lewy body’s dementia | 131038 | G31 |  |

1. *Data release period varied but similar for different countries in the United Kingdom; participants from England account for over 95% of the cohort.

**Table S3. A selection of UK Biobank cognitive tests**

| Cognitive domain | Cognitive test | Field ID | Baseline visit | First imaging visit | Web link | |
| --- | --- | --- | --- | --- | --- | --- |
| Processing speed | Reaction time | 20023 | G √ |  | | <https://biobank.ndph.ox.ac.uk/showcase/field.cgi?id=20023> |
| Working memory | Numeric memory | 4282 | G √ |  | | <https://biobank.ndph.ox.ac.uk/showcase/field.cgi?id=4282> |
| Verbal and numerical reasoning | Fluid intelligence | 20016 | G √ |  | | <https://biobank.ndph.ox.ac.uk/showcase/field.cgi?id=20016> |
| Prospective memory | Prospective memory | 20018 | G |  | | <https://biobank.ctsu.ox.ac.uk/crystal/field.cgi?id=20018> |
| Visual declarative memory | Pairs matching | 399 | G |  | | <https://biobank.ndph.ox.ac.uk/showcase/field.cgi?id=399> |
| Processing speed | Symbol digit substitution | 23324 |  | √ | | <https://biobank.ndph.ox.ac.uk/showcase/field.cgi?id=23324> |
| Executive function | Trail making part B | 6350 |  | √ | | <https://biobank.ndph.ox.ac.uk/showcase/field.cgi?id=6350> |
| Non-verbal reasoning | Matrix pattern completion | 6373 |  | √ | | <https://biobank.ndph.ox.ac.uk/showcase/field.cgi?id=6373> |

1. G: general cognitive ability element

**Table S4. Selected imaging derived phenotypes**

| Imaging derived phenotype (IDP) | UKB Field ID |
| --- | --- |
| Volume of hippocampus (from T1 brain image) | 25019/25020 |
| Volume of precuneus generated by parcellation of the white surface using Desikan-Killiany parcellation | 26812/26913 |
| Volume of cuneus generated by parcellation of the white surface using Desikan-Killiany parcellation | 26792/26893 |
| Volume of entorhinal generated by parcellation of the white surface using Desikan-Killiany parcellation | 26793/26894 |
| Volume of inferiorparietal generated by parcellation of the white surface using Desikan-Killiany parcellation | 26795/26896 |
| Volume of parahippocampal generated by parcellation of the white surface using Desikan-Killiany parcellation | 26803/26904 |
| Total volume of white matter hyperintensities (from T1 and T2_FLAIR images) | 25781 |
| Mean FA (fractional anisotropy) in anterior corona radiata on FA skeleton | 25079/25078 |
| Mean FA (fractional anisotropy) in anterior limb of internal capsule on FA skeleton | 25073/25072 |
| Mean FA (fractional anisotropy) in body of corpus callosum on FA skeleton | 25059 |
| Mean FA (fractional anisotropy) in cerebral peduncle on FA skeleton | 25071/25070 |
| Mean FA (fractional anisotropy) in cingulum cingulate gyrus on FA skeleton | 25091/25090 |
| Mean FA (fractional anisotropy) in cingulum hippocampus on FA skeleton | 25093/25092 |
| Mean FA (fractional anisotropy) in corticospinal tract on FA skeleton | 25063/25062 |
| Mean FA (fractional anisotropy) in external capsule on FA skeleton | 25089/25088 |
| Mean FA (fractional anisotropy) in fornix cres+stria terminalis on FA skeleton | 25095/25094 |
| Mean FA (fractional anisotropy) in fornix on FA skeleton | 25061 |
| Mean FA (fractional anisotropy) in genu of corpus callosum on FA skeleton | 25058 |
| Mean FA (fractional anisotropy) in inferior cerebellar peduncle on FA skeleton | 25067/25066 |
| Mean FA (fractional anisotropy) in medial lemniscus on FA skeleton | 25065/25064 |
| Mean FA (fractional anisotropy) in middle cerebellar peduncle on FA skeleton | 25056 |
| Mean FA (fractional anisotropy) in pontine crossing tract on FA skeleton | 25057 |
| Mean FA (fractional anisotropy) in posterior corona radiata on FA skeleton | 25083/25082 |
| Mean FA (fractional anisotropy) in posterior limb of internal capsule on FA skeleton | 25075/25074 |
| Mean FA (fractional anisotropy) in posterior thalamic radiation on FA skeleton | 25085/25084 |
| Mean FA (fractional anisotropy) in retrolenticular part of internal capsule on FA skeleton | 25077/25076 |
| Mean FA (fractional anisotropy) in sagittal stratum on FA skeleton | 25087/25086 |
| Mean FA (fractional anisotropy) in splenium of corpus callosum on FA skeleton | 25060 |
| Mean FA (fractional anisotropy) in superior cerebellar peduncle on FA skeleton | 25069/25068 |
| Mean FA (fractional anisotropy) in superior corona radiata on FA skeleton | 25081/25080 |
| Mean FA (fractional anisotropy) in superior fronto-occipital fasciculus on FA skeleton | 25099/25098 |
| Mean FA (fractional anisotropy) in superior longitudinal fasciculus on FA skeleton | 25097/25096 |
| Mean FA (fractional anisotropy) in tapetum on FA skeleton | 25103/25102 |
| Mean FA (fractional anisotropy) in uncinate fasciculus on FA skeleton | 25101/25100 |
| Mean MD (mean diffusivity) in anterior corona radiata on FA (fractional anisotropy) skeleton | 25127/25126 |
| Mean MD (mean diffusivity) in anterior limb of internal capsule on FA (fractional anisotropy) skeleton | 25121/25120 |
| Mean MD (mean diffusivity) in body of corpus callosum on FA (fractional anisotropy) skeleton | 25107 |
| Mean MD (mean diffusivity) in cerebral peduncle on FA (fractional anisotropy) skeleton | 25119/25118 |
| Mean MD (mean diffusivity) in cingulum cingulate gyrus on FA (fractional anisotropy) skeleton | 25139/25138 |
| Mean MD (mean diffusivity) in cingulum hippocampus on FA (fractional anisotropy) skeleton | 25141/25140 |
| Mean MD (mean diffusivity) in corticospinal tract on FA (fractional anisotropy) skeleton | 25111/25110 |
| Mean MD (mean diffusivity) in external capsule on FA (fractional anisotropy) skeleton | 25137/25136 |
| Mean MD (mean diffusivity) in fornix cres+stria terminalis on FA (fractional anisotropy) skeleton | 25143/25142 |
| Mean MD (mean diffusivity) in fornix on FA (fractional anisotropy) skeleton | 25109 |
| Mean MD (mean diffusivity) in genu of corpus callosum on FA (fractional anisotropy) skeleton | 25106 |
| Mean MD (mean diffusivity) in inferior cerebellar peduncle on FA (fractional anisotropy) skeleton | 25115/25114 |
| Mean MD (mean diffusivity) in medial lemniscus on FA (fractional anisotropy) skeleton | 25113/25112 |
| Mean MD (mean diffusivity) in middle cerebellar peduncle on FA (fractional anisotropy) skeleton | 25104 |
| Mean MD (mean diffusivity) in pontine crossing tract on FA (fractional anisotropy) skeleton | 25105 |
| Mean MD (mean diffusivity) in posterior corona radiata on FA (fractional anisotropy) skeleton | 25131/25130 |
| Mean MD (mean diffusivity) in posterior limb of internal capsule on FA (fractional anisotropy) skeleton | 25123/25122 |
| Mean MD (mean diffusivity) in posterior thalamic radiation on FA (fractional anisotropy) skeleton | 25133/25132 |
| Mean MD (mean diffusivity) in retrolenticular part of internal capsule on FA (fractional anisotropy) skeleton | 25125/25124 |
| Mean MD (mean diffusivity) in sagittal stratum on FA (fractional anisotropy) skeleton | 25135/25134 |
| Mean MD (mean diffusivity) in splenium of corpus callosum on FA (fractional anisotropy) skeleton | 25108 |
| Mean MD (mean diffusivity) in superior cerebellar peduncle on FA (fractional anisotropy) skeleton | 25117/25116 |
| Mean MD (mean diffusivity) in superior corona radiata on FA (fractional anisotropy) skeleton | 25129/25128 |
| Mean MD (mean diffusivity) in superior fronto-occipital fasciculus on FA (fractional anisotropy) skeleton | 25147/25146 |
| Mean MD (mean diffusivity) in superior longitudinal fasciculus on FA (fractional anisotropy) skeleton | 25145/25144 |
| Mean MD (mean diffusivity) in tapetum on FA (fractional anisotropy) skeleton | 25151/25150 |
| Mean MD (mean diffusivity) in uncinate fasciculus on FA (fractional anisotropy) skeleton | 25149/25148 |

**Table S5.** **Baseline characteristics of participants in the baseline or imaging cohort**

| **Characteristics** | **Baseline Cohort (*N*=435,046)** | **Imaging Cohort (*N*=43,390)** |
| --- | --- | --- |
| **Baseline age, years (mean** ± **SD)** | 56.8 ± 8 | 55.3 ± 7.5 |
| **Sex, female (%)** | 236,117 (54%) | 22,373 (52%) |
| **Education (%)** |  |  |
| **None** | 75,541 (18%) | 2,984 (7%) |
| **CSEs or equivalent** | 16,231 (4%) | 1,122 (3%) |
| **O levels/GCSEs or equivalent** | 59,497 (13%) | 4,705 (11%) |
| **A/AS levels/NVQ/HND/HNC** | 79,609 (18%) | 7,778 (18%) |
| **Other professional qualifications** | 64,394 (15%) | 6,946 (16%) |
| **College or university degree** | 137,941 (32%) | 19,721 (46%) |
| **Townsend deprivation index** | -1.5 ± 3 | -1.9 ± 2.7 |
| **BMI, kg/m^2^ (mean ± SD**) | 27.4 ± 4.8 | 26.6 ± 4.3 |
| **Smoking status (%)** |  |  |
| **Never** | 234,004 (54%) | 26,022 (60%) |
| **Previous** | 154,156 (36%) | 14,596 (34%) |
| **Current** | 45,343 (10%) | 2,687 (6%) |
| **Alcohol intake frequency (%)** |  |  |
| **Never** | 29,060 (7%) | 1,847 (4%) |
| **Special occasions only** | 46,553 (11%) | 3,323 (8%) |
| **1-3 times a month** | 48,359 (11%) | 4,619 (11%) |
| **1-2 times a week** | 114,513 (26%) | 11,195 (26%) |
| **3-4 times a week** | 104,311 (24%) | 12,345 (28%) |
| **Daily or almost daily** | 91,947 (21%) | 10,053 (23%) |
| **IPAQ activity group (%)** |  |  |
| **Low** | 57,696 (14%) | 5,742 (14%) |
| **Moderate** | 177,073 (44%) | 18,504 (45%) |
| **High** | 163,435 (41%) | 16,902 (41%) |
| ***APOE* genotype (%)** |  |  |
| **e3e3** | 254,331 (58.46%) | 25,619 (59.04%) |
| **e2e3** | 53,573 (12.31%) | 5,388 (12.42%) |
| **e2e2** | 2,766 (0.64%) | 250 (0.58%) |
| **e2e4** | 10,931 (2.51%) | 1,036 (2.39%) |
| **e3e4** | 103,077 (23.69%) | 10,122 (23.33%) |
| **e4e4** | 10,350 (2.38%) | 975 (2.25%) |
| **e1e2** | 3 (<0.01%) | 0 (0.00%) |
| **Telomere length (T/S ratio), adjusting for technical parameters** | 0.83 ± 0.13 | 0.84 ± 0.13 |

1. Abbreviations: SD, standard deviation; *APOE*, apolipoprotein E; CSE, certificate of secondary education; GCSE, general certificate of secondary education; NVQ, national vocational qualification; HND, higher national diploma; HNC, higher national certificate. BMI, body mass index; IPAQ, International Physical Activity Questionnaire.

**Table S6. Participant characteristics at baseline of incident AD/ADRD cases versus normal controls**

| **Characteristics** | **AD/ADRD**  **(*N*=6,424)** | **Normal Controls**  **(*N*=428,622)** | **P-Value** |
| --- | --- | --- | --- |
| **Baseline age, years (mean** ± **SD)** | 64.0 ± 5.1 | 56.7 ± 8.0 | < 2.2×10^-16^ |
| **Sex, female (%)** | 2,973 (46%) | 233,144 (54%) | < 2.2×10^-16^ |
| **Education (%)** |  |  | < 2.2×10^-16^ |
| **None** | 2,276 (36%) | 73,265 (17%) |  |
| **CSEs or equivalent** | 119 (2%) | 16,112 (4%) |  |
| **O levels/GCSEs or equivalent** | 817 (13%) | 56,680 (13%) |  |
| **A/AS levels/NVQ/HND/HNC** | 998 (16%) | 78,611 (19%) |  |
| **Other professional qualifications** | 845 (13%) | 63,549 (15%) |  |
| **College or university degree** | 1,246 (20%) | 136,695 (32%) |  |
| **Townsend deprivation index** | -0.9 ± 3.3 | -1.5 ± 3.0 | 7.3×10^-38^ |
| **BMI, kg/m^2^ (mean ± SD**) | 27.8 ± 5.0 | 27.4 ± 4.8 | 9.3×10^-12^ |
| **Smoking status (%)** |  |  | < 2.2×10^-16^ |
| **Never** | 2,842 (45%) | 231,162 (54%) |  |
| **Previous** | 2,777 (44%) | 151,379 (35%) |  |
| **Current** | 762 (12%) | 44,581 (10%) |  |
| **Alcohol intake frequency (%)** |  |  | < 2.2×10^-16^ |
| **Never** | 826 (13%) | 28,234 (7%) |  |
| **Special occasions only** | 875 (14%) | 45,678 (11%) |  |
| **1-3 times a month** | 636 (10%) | 47,723 (11%) |  |
| **1-2 times a week** | 1,471 (23%) | 113,042 (26%) |  |
| **3-4 times a week** | 1,182 (18%) | 103,129 (24%) |  |
| **Daily or almost daily** | 1,420 (22%) | 90,527 (21%) |  |
| **IPAQ activity group (%)** |  |  | 7.6×10^-7^ |
| **Low** | 925 (16%) | 56,771 (14%) |  |
| **Moderate** | 2,556 (45%) | 174,517 (44%) |  |
| **High** | 2,144 (38%) | 161,291 (41%) |  |
| ***APOE* genotype (%)** |  |  | < 2.2×10^-16^ |
| **e3e3** | 2,651 (41.27%) | 251,680 (59.72%) |  |
| **e2e3** | 473 (7.36%) | 53,100 (12.39%) |  |
| **e2e2** | 30 (0.47%) | 2,736 (0.64%) |  |
| **e2e4** | 159 (2.48%) | 10,772 (2.51%) |  |
| **e3e4** | 2,449 (38.12%) | 100,628 (23.48%) |  |
| **e4e4** | 662 (10.31%) | 9,688 (2.26%) |  |
| **e1e2** | 0 (0.00%) | 3 (<0.01%) |  |
| **Telomere length (T/S ratio), adjusting for technical parameters** | 0.80 ± 0.12 | 0.83 ± 0.13 | < 2.2×10^-16^ |

1. Abbreviations: SD, standard deviation; *APOE*, apolipoprotein E; CSE, certificate of secondary education; GCSE, general certificate of secondary education; NVQ, national vocational qualification; HND, higher national diploma; HNC, higher national certificate. BMI, body mass index; IPAQ, International Physical Activity Questionnaire.
2. Two-sided two-sample t-test p-values for continuous variables and chi-square test p-values for categorical variables.

**Table S7. Participant characteristics at baseline of incident Alzheimer’s disease (AD) cases versus normal controls**

| **Characteristics** | **AD**  **(*N*=1,225)** | **Normal Controls**  **(*N*=428,622)** | **P-Value** |
| --- | --- | --- | --- |
| **Baseline age, years (mean** ± **SD)** | 64.6 ± 4.2 | 56.7 ± 8 | < 2.2×10^-16^ |
| **Sex, female (%)** | 666 (54%) | 233,144 (54%) | >0.999 |
| **Education (%)** |  |  | < 2.2×10^-16^ |
| **None** | 416 (35%) | 73,265 (17%) |  |
| **CSEs or equivalent** | 24 (2%) | 16,112 (4%) |  |
| **O levels/GCSEs or equivalent** | 167 (14%) | 56,680 (13%) |  |
| **A/AS levels/NVQ/HND/HNC** | 187 (16%) | 78,611 (19%) |  |
| **Other professional qualifications** | 180 (15%) | 63,549 (15%) |  |
| **College or university degree** | 228 (19%) | 136,695 (32%) |  |
| **Townsend deprivation index** | -1.4 ± 3.1 | -1.5 ± 3.0 | 0.210 |
| **BMI, kg/m^2^ (mean ± SD**) | 27.3 ± 4.8 | 27.4 ± 4.8 | 0.446 |
| **Smoking status (%)** |  |  | 6.98×10^-9^ |
| **Never** | 588 (48%) | 231,162 (54%) |  |
| **Previous** | 532 (44%) | 151,379 (35%) |  |
| **Current** | 98 (8%) | 44,581 (10%) |  |
| **Alcohol intake frequency (%)** |  |  | 8.31×10^-10^ |
| **Never** | 132 (11%) | 28,234 (7%) |  |
| **Special occasions only** | 164 (13%) | 45,678 (11%) |  |
| **1-3 times a month** | 121 (10%) | 47,723 (11%) |  |
| **1-2 times a week** | 313 (26%) | 113,042 (26%) |  |
| **3-4 times a week** | 248 (20%) | 103,129 (24%) |  |
| **Daily or almost daily** | 246 (20%) | 90,527 (21%) |  |
| **IPAQ activity group (%)** |  |  | 0.148 |
| **Low** | 138 (13%) | 56,771 (14%) |  |
| **Moderate** | 483 (44%) | 174,517 (44%) |  |
| **High** | 473 (43%) | 161,291 (41%) |  |
| ***APOE* genotype (%)** |  |  | < 2.2×10^-16^ |
| **e3e3** | 366 (29.88%) | 251,680 (58.72%) |  |
| **e2e3** | 54 (4.41%) | 53,100 (12.39%) |  |
| **e2e2** | 3 (0.24%) | 2,736 (0.64%) |  |
| **e2e4** | 25 (2.04%) | 10,772 (2.51%) |  |
| **e3e4** | 587 (47.92%) | 100,628 (23.48%) |  |
| **e4e4** | 190 (15.51%) | 9,688 (2.26%) |  |
| **e1e2** | 0 (0%) | 3 (<0.01%) |  |
| **Telomere length (T/S ratio), adjusting for technical parameters** | 0.8 ± 0.11 | 0.83 ± 0.13 | < 2.2×10^-16^ |

1. Abbreviations: SD, standard deviation; *APOE*, apolipoprotein E; CSE, certificate of secondary education; GCSE, general certificate of secondary education; NVQ, national vocational qualification; HND, higher national diploma; HNC, higher national certificate. BMI, body mass index; IPAQ, International Physical Activity Questionnaire.
2. Two-sided two-sample t-test p-values for continuous variables and chi-square test p-values for categorical variables.

**Table S8. Participant characteristics at baseline of incident vascular dementia (VD) cases versus normal controls**

| **Characteristics** | **VD**  **(*N*=602)** | **Normal Controls**  **(*N*=428,622)** | **P-Value** |
| --- | --- | --- | --- |
| **Baseline age, years (mean** ± **SD)** | 64.5 ± 4.5 | 56.7 ± 8 | < 2.2×10^-16^ |
| **Sex, female (%)** | 255 (42%) | 233,144 (54%) | 4.02×10^-9^ |
| **Education (%)** |  |  | < 2.2×10^-16^ |
| **None** | 235 (40%) | 73,265 (17%) |  |
| **CSEs or equivalent** | 9 (2%) | 16,112 (4%) |  |
| **O levels/GCSEs or equivalent** | 68 (11%) | 56,680 (13%) |  |
| **A/AS levels/NVQ/HND/HNC** | 104 (18%) | 78,611 (19%) |  |
| **Other professional qualifications** | 76 (13%) | 63,549 (15%) |  |
| **College or university degree** | 100 (17%) | 136,695 (32%) |  |
| **Townsend deprivation index** | -0.7 ± 3.5 | -1.5 ± 3 | 9.54×10^-8^ |
| **BMI, kg/m^2^ (mean ± SD**) | 28.8 ± 5.3 | 27.4 ± 4.8 | 1.37×10^-10^ |
| **Smoking status (%)** |  |  | 1.10×10^-11^ |
| **Never** | 236 (40%) | 231,162 (54%) |  |
| **Previous** | 285 (48%) | 151,379 (35%) |  |
| **Current** | 71 (12%) | 44,581 (10%) |  |
| **Alcohol intake frequency (%)** |  |  | 1.53×10^-13^ |
| **Never** | 82 (14%) | 28,234 (7%) |  |
| **Special occasions only** | 83 (14%) | 45,678 (11%) |  |
| **1-3 times a month** | 55 (9%) | 47,723 (11%) |  |
| **1-2 times a week** | 153 (26%) | 113,042 (26%) |  |
| **3-4 times a week** | 97 (16%) | 103,129 (24%) |  |
| **Daily or almost daily** | 128 (21%) | 90,527 (21%) |  |
| **IPAQ activity group (%)** |  |  | 2.90×10^-4^ |
| **Low** | 109 (21%) | 56,771 (14%) |  |
| **Moderate** | 217 (41%) | 174,517 (44%) |  |
| **High** | 202 (38%) | 161,291 (41%) |  |
| ***APOE* genotype (%)** |  |  | < 2.2×10^-16^ |
| **e3e3** | 262 (43.52%) | 251,680 (58.72%) |  |
| **e2e3** | 45 (7.48%) | 53,100 (12.39%) |  |
| **e2e2** | 3 (0.5%) | 2,736 (0.64%) |  |
| **e2e4** | 18 (2.99%) | 10,772 (2.51%) |  |
| **e3e4** | 219 (36.38%) | 100,628 (23.48%) |  |
| **e4e4** | 55 (9.14%) | 9,688 (2.26%) |  |
| **e1e2** | 0 (0%) | 3 (0%) |  |
| **Telomere length (T/S ratio), adjusting for technical parameters** | 0.79 ± 0.12 | 0.83 ± 0.13 | < 2.2×10^-16^ |

1. Abbreviations: SD, standard deviation; *APOE*, apolipoprotein E; CSE, certificate of secondary education; GCSE, general certificate of secondary education; NVQ, national vocational qualification; HND, higher national diploma; HNC, higher national certificate. BMI, body mass index; IPAQ, International Physical Activity Questionnaire.
2. Two-sided two-sample t-test p-values for continuous variables and chi-square test p-values for categorical variables.
